# Salting-Out Approach Is Worthy of Comparison with Ultracentrifugation for Extracellular Vesicle Isolation from Tumor and Healthy Models

**DOI:** 10.3390/biom11121857

**Published:** 2021-12-10

**Authors:** Simona Serratì, Antonio Palazzo, Annamaria Lapenna, Helena Mateos, Antonia Mallardi, René Massimiliano Marsano, Alessandra Quarta, Mario Del Rosso, Amalia Azzariti

**Affiliations:** 1 Nanotecnology Laboratory, IRCCS Istituto Tumori Giovanni Paolo II, Viale Orazio Flacco 65, 70124 Bari, Italy; a.azzariti@oncologico.bari.it; 2 Department of Chemistry, University of Bari and CSGI (Center for Colloid and Surface Science), Via Orabona 4, 70125 Bari, Italy; annamaria.lapenna@uniba.it (A.L.); helena.mateos@uniba.it (H.M.); 3 Istituto per i Processi Chimico Fisici, National Research Council (IPCF-CNR), c/o ChemistryDepartment, Via Orabona 4, 70125 Bari, Italy; a.mallardi@ba.ipcf.cnr.it; 4 Department of Biology, University of Bari Aldo Moro, Via Orabona 4, 70125 Bari, Italy; renemassimiliano.marsano@uniba.it; 5 CNR NANOTEC—Istituto di Nanotecnologia, National Research Council (CNR), Via Monteroni, 73100 Lecce, Italy; alessandra.quarta@nanotec.cnr.it; 6 Department of Experimental and Clinical Biomedical Sciences “Mario Serio”, University of Florence, Viale G.B. Morgagni 50, 50134 Florence, Italy; delrosso@unifi.it; 7 Laboratory of Experimental Pharmacology, IRCCS Istituto Tumori Giovanni Paolo II, Viale Orazio Flacco 65, 70124 Bari, Italy

**Keywords:** extracellular vesicles, ultracentrifugation, salting-out method

## Abstract

The role of extracellular vesicles (EVs) has been completely re-evaluated in the recent decades, and EVs are currently considered to be among the main players in intercellular communication. Beyond their functional aspects, there is strong interest in the development of faster and less expensive isolation protocols that are as reliable for post-isolation characterisations as already-established methods. Therefore, the identification of easy and accessible EV isolation techniques with a low price/performance ratio is of paramount importance. We isolated EVs from a wide spectrum of samples of biological and clinical interest by choosing two isolation techniques, based on their wide use and affordability: ultracentrifugation and salting-out. We collected EVs from human cancer and healthy cell culture media, yeast, bacteria and *Drosophila* culture media and human fluids (plasma, urine and saliva). The size distribution and concentration of EVs were measured by nanoparticle tracking analysis and dynamic light scattering, and protein depletion was measured by a colorimetric nanoplasmonic assay. Finally, the EVs were characterised by flow cytometry. Our results showed that the salting-out method had a good efficiency in EV separation and was more efficient in protein depletion than ultracentrifugation. Thus, salting-out may represent a good alternative to ultracentrifugation.

## 1. Introduction

All living organisms use different types of highly specialized intercellular communication to best finalize the transport of signals and biological materials to ensure the correct functioning of cells, tissues, organs and systems. Remote communication that is mediated by extracellular vesicles (EVs), which is known as “cell-to-cell contact-free communication” [1], has aroused great interest in the last decade because of its involvement in the most important biological processes, such as antigen presentation without contact, distant cell education, microenvironment modification, immunity [2], inflammation [3], carcinogenesis [4], cardiovascular diseases [5] and all physio-pathological processes.

The family of EVs includes three main subtypes of vesicles, based on their biogenesis and size: exosomes, microvesicles and apoptotic bodies [6,7,8,9]. Microvesicles are large vesicles with a diameter in the range of 50–2000 nm [10] that are directly generated through outward budding of the cell plasma membrane [11]. Apoptotic bodies, with a size from 50 nm to 5000 nm in diameter, are released upon programmed cell death by membrane blebbing [12]. Finally, exosomes represent the smallest EVs (40–150 nm) that originate from the fusion of multivesicular bodies with the plasma membrane [13]. Considering their lipid composition, more exosome subclasses can be distinguished. The lipid composition seems to be cell-type dependent [14], and up to 540 different lipids, divided into 36 classes and subclasses, have been identified in exosome-like vesicles derived from a single cancer cell line [15].

All living organisms, ranging from bacteria to fungi and from germ line to neurons in metazoans, release EVs [16,17,18]. In-depth studies have also been carried out on yeast, which is an excellent model system to study the trafficking of different types of EVs [19], such as post-Golgi vesicles (PGVs), and their application in the biomedical field [20]. Likewise, *C. elegans* early embryos and *Drosophila* secretory glands are tissues where the release of EVs is predominant; for this reason, they are crucial models for the comprehension of the generation, behavioral and functional roles of EVs [21].

In humans, EVs are present in abundance in many biological fluids, including blood, urine, saliva, milk, semen, bile juice, ascites, cystic, bronchoalveolar and gastrointestinal lavage fluid [22]. They are secreted by all cell types in both healthy and disease conditions [23].

EVs have received considerable attention, not only due to their role in cell-to-cell communication, but also in the development of early diagnostic tests based on cellular biomarkers that are expressed and/or transported in a response to cell pathological conditions [24]. The involvement of EVs in physio-pathological processes [6] has opened new perspectives in translational research, and much of the knowledge obtained from in vitro studies has been proposed for application in clinical research as well as in daily hospital routines [25,26]. EVs from body fluids could be a non-invasive alternative to regular biopsies. “Liquid biopsy” resolves some of the most important limitations of the surgical biopsy, which include the accessibility to the sick tissue, the possibility of evaluating the status of the disease (depending on the EV content), and the reduction of the biopsy-associated stress caused to the patient [27,28,29]. The broad application spectrum of EVs could range from diagnostics to therapy, including the monitoring of disease and finding useful predictive or prognostic biomarkers; however, a significant gap remains between basic research and the clinical setting [1].

Both the complex nature of samples and the heterogeneity of EV populations limit the progress of this branch of science. The boundary between EV classes is far from clear, which is reflected in the challenge of finding reliable and reproducible purification protocols. Along with the heterogeneity in EV populations, the presence of contaminants in samples, such as proteins, might affect the physicochemical and functional characteristics of EVs, thereby inducing a misinterpretation of the information and compromising the quality of reported data [30]. To elucidate EV functionalities and their potential pharmaceutical uses, the development of new, easy and accessible isolation protocols with a low price/performance ratio that are able to provide pure and homogeneous vesicle subtypes is of paramount importance in this field. Considerable attention has been focused on this aspect, and many studies have compared isolation techniques or described new techniques [31].

To date, there are different protocols to isolate EVs which are based on physical, immunological or chemical approaches [23].

Ultracentrifugation (UC) methods (with or without gradient), based on differential centrifugation (DC), represent the most commonly used method for purifying EVs. However, it is considered a versatile technique because any kind of fluid could be processed, with an accessible per-sample processing cost. Moreover, it could be possible to optimize the DC protocol according to the complexity of the sample, to improve the separation efficiency. In particular, the number of centrifugation steps could be increased. However, this process may lead to EV damage and thus a reduction in particle yield, as well as an increase in isolation time. In addition, UC has other important disadvantages: it is time-consuming and requires expensive instrumentation [32]. Moreover, protein coprecipitation is a strong limit of this approach [23]. In this regard, several commercial isolation kits based on charge neutralization precipitation, gel filtration, affinity purification using magnetic beads, etc., are commercially available. They allow the purification of EVs but have a high cost per sample and are specific to biological fluids.

In 2014, Brownlee et al. proposed a salting-out (S-O) approach based on exosome precipitation with sodium acetate at a low pH and their resuspension in acetate-free buffer at neutral pH [33]. In particular, the salting-out approach is a method based on the precipitation of EVs from solution by neutralizing their surface charge with acetate. EVs express negatively charged phospholipid phosphatidylserine (PS) [34], and the titration of culture supernatants with 0.1 M acetate to pH 4.75 resulted in the immediate precipitation of virtually all EVs [33]. Then, the precipitated EVs were washed to remove residual media and resuspended in acetate-free buffer at neutral pH.

S-O could represent a good alternative to UC because it has a negligible cost per sample, allows for fast isolation from large-volume samples and requires common equipment for isolation procedures. Currently, its application is limited by insufficient information about sample contamination with non-EV-associated proteins, and by the lack of exhaustive information regarding the quality of the samples obtained with this approach.

The present work specifically compared the EV isolation efficiency of these two different procedures, which were assessed via a nanoparticle tracking analysis (NTA), dynamic light scattering (DLS) and flow cytometry (FCM). Moreover, to assess the depletion of protein contaminants, a recently proposed nanoplasmonic colorimetric assay (CONAN) has been used [35]. EVs were sourced from both cancer and healthy human cell line-conditioned media and from blood, urine and saliva, the three most commonly used sample body fluids. In addition, we extended this comparative study to conditioned media obtained from cell culture models commonly used in basic research, such as insects, bacteria and yeast. This process allows us to verify their suitability, with respect to cell cultures commonly used as model systems in basic research.

## 2. Materials and Methods

### 2.1. Cell Cultures and Conditioned Medium

S2R^+^ cells (DGRC, Bloomington, IN, USA) were grown in Schneider’s insect medium supplemented with 10% FBS and 1% penicillin/streptomycin at 25 °C without CO_2_.

The human colorectal adenocarcinoma cell lines Colo 205 and Caco-2 (ATCC CCL-222 and ATCC HTB-37, LGC Standards S.r.l., Milano, Italy) were cultured in RPMI-1640 medium (Euroclone, Milano, Italy) and in Dulbecco’s modified Eagle’s medium (DMEM, Euroclone, Milano, Italy), respectively. The human breast ductal epithelium cell line HB2 and the breast cancer cell line 8701-BC (both generously provided by Proff. Pucci-Minafra-Istituto di Istologia ed Embriologia, Università di Palermo, Italy [36,37]) were grown in complete DMEM medium as described above and supplemented with 5 µg/mL hydrocortisone (Sigma-Aldrich, Merk Life Science S.r.l., Milano, Italy) and 10 µg/mL bovine insulin (Sigma-Aldrich, Merk Life Science S.r.l., Milano, Italy).

All human cell lines were cultured in complete medium, supplemented with 10% FBS, 2 mM glutamine (Euroclone, Milano, Italy), 100 UI/mL penicillin and 100 µg/mL streptomycin (Euroclone, Milano, Italy) at 37 °C in a humidified atmosphere, containing 5% CO_2_. The FBS that was used in all cell cultures was exosome-depleted (EXO-FBS-250A-1, System Biosciences, Abingdon, UK). The conditioned medium was obtained after incubation for 48 h with human and insect cell lines.

BY4742 (MATα his3Δ1 leu2Δ0 lys2Δ0 ura3Δ0) (ATCC 201389), a laboratory strain derived from S288C [38], was cultured as follows. Cells from a preculture in YPD medium were washed three times with PBS. Filtered fresh medium was inoculated with 1/100 (vol/vol) washed cells and incubated at 25 °C for 20 h. Then, the supernatant was collected and processed for EV isolation. LE392 (*hsd*R514(r_k_^−^, m_k_^+^), *gln*V(*sup*E44), *try*T (*sup*F58), *lac*Y1 or Δ*lac*IZY)6, *gal*K2, *gal*T22, *met*B1, *trp*R55) strain (ATCC 204508) was cultured in standard Luria-Bertani medium. Filtered fresh medium was inoculated with 1/100 (vol/vol) of a preculture previously washed three times. Cells grew exponentially at 37 °C until an OD of 0.6–0.8 was obtained.

### 2.2. Human Fluids

Four milliliters of plasma from one healthy individual, 2 mL of saliva diluted 1:1 in volume with sterile PBS and 50 mL of urine were provided by the institutional BioBank of the IRCCS IstitutoTumori Giovanni Paolo II in Bari, Italy. All human samples were collected in accordance with Italian guidelines and regulations, in two studies approved by the ethics committee of the IRCCS IstitutoTumori Giovanni Paolo II, Bari, Italy (Prot. 4864/2020 and Prot. 711/CE), and written informed consent was obtained from all patients enrolled in the study.

### 2.3. EVs Isolation

Cell-conditioned media and biological fluids (saliva, plasma and urine) were cleared of cells, cell debris and large vesicles by sequential centrifugation. The collected fluids were centrifuged at 300× *g* for 5 min to discard the cell pellet. The supernatant was transferred to a fresh 50 mL tube and centrifuged at 2600× *g* for 15 min to remove cell debris and apoptotic bodies as previously described [39].The supernatant was divided into two equal aliquots that were subjected to the two different isolation methods.

Ultracentrifugation was performed according to the Thery protocol [40]. The cleared medium was ultracentrifuged at 10,000× *g* for 30 min, and the supernatant was collected in a new tube and centrifuged at 100,000× *g* for 70 min. The pellet was washed with sterile PBS and then centrifuged again at 100,000× *g* for 70 min. All centrifugation steps were carried out at 4 °C. Finally, the pellets were resuspended in a final volume of 100 µL of PBS, which was supplemented with a protease inhibitor cocktail (Sigma Aldrich, St. Louis, MO, USA). The UC steps were performed with a Beckman Coulter Optima–XPN 100 ultracentrifuge, TYPE 70 Ti rotor, using 26. 3 mL polycarbonate bottles (Beckman Coulter, Milan, Italy; cat. no. 355618).

The salting-out protocol was performed according to the Brownlee isolation method [33]. A 1/10th volume of sodium acetate (1 M, pH 4.7) was mixed with the cleared medium, incubated on ice for at least 30 min and then transferred to 37 °C for an additional 5 min. Samples were centrifuged at 5000× *g* for 10 min, and the vesicles were collected at the bottom of the tube. The pellet was resuspended in 0.1 M sodium acetate buffer (pH 4.7). The suspension was again centrifuged at 5000× *g* for 10 min, and the pellet was solubilized in HBS buffer (NaCl 150 mM, HEPES 20 mM).

The purified vesicles were stored for up to 10 days at 4 °C or −20 °C for longer periods. In Table S1, the EVs yields are reported.

### 2.4. Nanoparticle Tracking Analysis (NTA)

The size distribution and concentration of EVs were measured by NTA (NS300, Malvern Instruments LTD, Rome, Italy). Before analysing our samples, as a routine practice, we carried out a verification of the instrument by measuring the 100 nm polystyrene latex microspheres (Malvern) as a control. All samples were diluted in PBS to a final volume of 1 mL (dilution conditions: 1:1000). The instrument was set according to the manufacturer’s software manual (NanoSight NS300 User Manual, MAN0516-08-EN-00, January 2016). The temperature was set to 25 °C, and the camera level was increased (between 10 and 14) until all particles were distinctly visible. The ideal detection threshold was determined to include as many particles as possible, with the restriction that 10–100 red crosses were counted and only <10% were not associated with distinct particles. The blue crosses count was limited to 5. In our measurements, the detection threshold values were between 5 and 11. Given the variability of the settings, due to the heterogeneity of the samples, we had a dedicated person in the laboratory to develop and maintain a streamlined and standardised execution of analysis, thereby obtaining acquaintance with the appropriate monitoring of the instrument. All samples were analysed in triplicate: 60 sec video images were acquired and analysed by NanoSight NTA 3.2 software.

### 2.5. Dynamic Light Scattering (DLS)

Dynamic light scattering (DLS) measurements were performed using a Zetasizer-Nano S from Malvern that was equipped with a 4 mW He-Ne laser (λ = 633 nm), operating at a fixed detector angle of 173° (non-invasive backscattering geometry NIBSTM) and with the cell holder maintained at 25 °C by means of a Peltier element. At least three measurements were acquired for each sample, and each reading was composed of 12 runs. Before measurements, EV samples were diluted (2- to 30-fold) in PBS, gently mixed and loaded in disposable microvolume cuvettes. The intensity-weighted size distribution was retrieved from the field autocorrelation function by inverse Laplace transformation, using a non-negative least squares algorithm (Multiple Narrow Modes) implemented by the manufacturer. The number-weighted size distribution was evaluated from the intensity-weighted size distribution, considering the vesicle form factor as detailed elsewhere [35].

### 2.6. Transmission Electron Microscopy (TEM) Imaging

A drop of EVs suspension was deposited onto a 300-mesh carbon coated copper TEM grid. Then, the grid was stained with 1% osmium tetroxide for 1 min prior to being rinsed with ultrapure water and then being let to dry. Low-magnification images were recorded on a JEOL Jem1011 microscope, operating at an accelerating voltage of 100 kV. The average size of the vesicles was estimated through Image J Software on TEM images.

### 2.7. Flow Cytometry: Sample Preparation and Data Acquisition 

FCM instrument preparation and setup were carried out following Gorgun’s protocol, as previously described [41,42] and which is reported in Supplementary File S1. For tetraspanin determination, each sample was preincubated with 5 μL of Super Bright Complete Staining Buffer (E-Biosciences, Invitrogen, Waltham, MA, USA), to prevent nonspecific polymer interactions, for 30 min in the dark at 2−8 °C. Then, the EVs were labelled with 5µL of CD63-PE-Cy7-conjugated and CD81-APC-conjugated anti-human mAb (e-Biosciences, Invitrogen) and stored for 30 min in a dark room at 2–8 °C. For ALIX determination, EVs were pretreated with 0.1% TritonX-100 for 10 min. After permeabilisation, the EVs were incubated with Super Bright Complete Staining Buffer, as described above, and then, were labelled with 4 µL of anti-ALIX (BioLegend, San Diego, CA, USA) for 30 min in a dark room at 2–8 °C. The secondary antibody anti-mouse Alexa Fluor 488 (Molecular Probes, Life Technologies) was added for 30 min in a dark room at 2–8 °C. After staining, EVs were collected and analysed using an AttuneNxT Acoustic Focusing Cytometer (Thermo Fisher, Life Technologies Italia, Monza, Italy) equipped with four lasers (405 nm violet, 488 nm blue, 561 nm yellow, and 637 nm red) for sample reading. The final data were analysed using AttuneNxT Analysis Software (Thermo Fisher, Life Technologies Italia, Monza, Italy) [43].

### 2.8. Colorimetric Nanoplasmonic (CONAN) Assay 

The protein depletion of EV samples was assessed by means of a colorimetric nanoplasmonic assay, based on the interaction between EVs and gold nanoparticles (AuNPs) [44].

AuNPs were synthesized in Milli-Q water following the Turkevich method [45]. In detail, 2 mL of 1% Na-citrate was rapidly added to 20 mL of a boiling solution of 1 mM HAuCl_4_ under fast stirring. After 5 min, the reaction was stopped by cooling down the AuNP solution with a water–ice bath. The UV–vis spectrum of the AuNP solution was registered between 400 and 950 nm using a JASCO V-530 spectrophotometer. To determine the concentration, the absorbance at the resonance wavelength of the surface plasmon (520 nm) was considered (Ɛ = 4 × 10^8^ M^−1^ cm^−1^).

For the assay, EVs were suitably diluted in PBS and then incubated at room temperature for 10 min with AuNPs (at a final concentration of 1 nM). The UV–vis spectra of the solutions were collected in the region between 400 and 950 nm, and AuNP aggregation was quantified through the aggregation index value (AI). This value can be extrapolated from the nanoparticle absorption spectra and is defined as the ratio of absorbance at 520 nm and the sum of absorbance at 650 nm and 900 nm.

(1) $$AI=\frac{Abs\;\left(520\;\mathrm{nm}\right)}{Abs\;\left(650\;\mathrm{nm}\right)+Abs\;\left(900\;\mathrm{nm}\right)}$$

Depending on the nanoparticle aggregation, the contribution at high wavelengths is not always negligible. Therefore, the absorbance value at 900 nm was taken into account. According to this description, a higher value of AI refers to a stable solution of AuNPs which are characterised by a pink colour, while a lower AI indicates an increase in nanoparticle aggregation.

In this paper, the AI of the samples is reported as the percentage of the ratio between the AI of the sample and the AI of monodispersed AuNPs.

(2) $$AI\;ratio=\frac{AI\;sample}{AI\;AuNPs}\;\%$$

In the presence of pure EVs, AuNPs adsorb and cluster at their lipid membrane. This results in a redshift of the plasmonic absorption, as well as in a change in the colour of the solution from pink to blue. In the case of a very concentrated EV preparation, the amount of AuNPs is not enough to come into contact, and the NPs adsorbed on the vesicle lipid membrane are so far apart that clustering is negligible. AuNP aggregation is also prevented in the presence of protein contaminants that are able to adsorb on the surface of AuNPs, forming a nanoparticle–protein corona complex. In both situations, the surface plasmon resonance peak remains unchanged with respect to that of the dispersed AuNPs, and the solution remains pink. As the colour pink of the solution can be due to either the presence of proteins or a high concentration of EVs, consecutive dilutions of the sample have to be prepared to distinguish between these two situations. In the absence of protein contamination, dilutions of EVs lower than 1 × 10^8^ particles/mL will allow the clustering of AuNPs at the EV surface, and the solution will turn blue. According to this hypothesis, if none of the dilutions turns blue, the sample is contaminated.

### 2.9. Western Blot Analysis

Fifty micrograms of fresh total lysates, measured with the Bio-Rad Protein assay Dye Reagent (BioRad Laboratories, Milano, Italy), which is a colorimetric assay based on the Bradford method, were run on a 4–20% Mini-Protean TGX Stain-Free Gel (Bio-Rad Laboratories, Milano, Italy). The protein was transferred to a nitrocellulose membrane (Trans-Blot Turbo Mini Nitrocellulose, Bio-Rad Laboratories, Milano, Italy) using the Trans-Blot Turbo Transfer System (Bio-Rad Laboratories, Milano, Italy). The membrane was blocked and then incubated overnight at 4 °C with primary antibodies against CD81 (SN206, Novusbio, Littleton, Colorado) and against Calnexin (C5C9, Cell Signaling, Danvers, MA, USA). The following day, the membranes were incubated with secondary antibodies (#170-6515, rabbit-HRP, BioRad, Milano, Italy). Blot detection was performed with ChemiDoc Imaging Systems.

### 2.10. Statistical Analyses

The data were analysed using GraphPad Prism vers 5.0 to calculate the significance between groups using the paired Students’s *t*-test method (two-tailed). Statistically significant differences were set at probabilities of * *p* < 0.05, ** *p* < 0.01 and *** *p* < 0.001.

## 3. Results

This work is a comparative study of two different procedures to separate EVs from different sources. The efficiency of UC versus S-O in the isolation of EVs has been examined in depth based on the physical and biochemical or compositional characteristics of EVs.

### 3.1. Physical Characteristics of EVs

The concentration and/or the size distribution of the EVs were determined in solution by means of two techniques: NTA and DLS. Details on the physical basis of these techniques can be found elsewhere [46,47]. Note that some samples are very dilute, and the photons collected are insufficient for DLS analysis (not suitable) but still suitable for NTA measurements.

**Figure 1:**
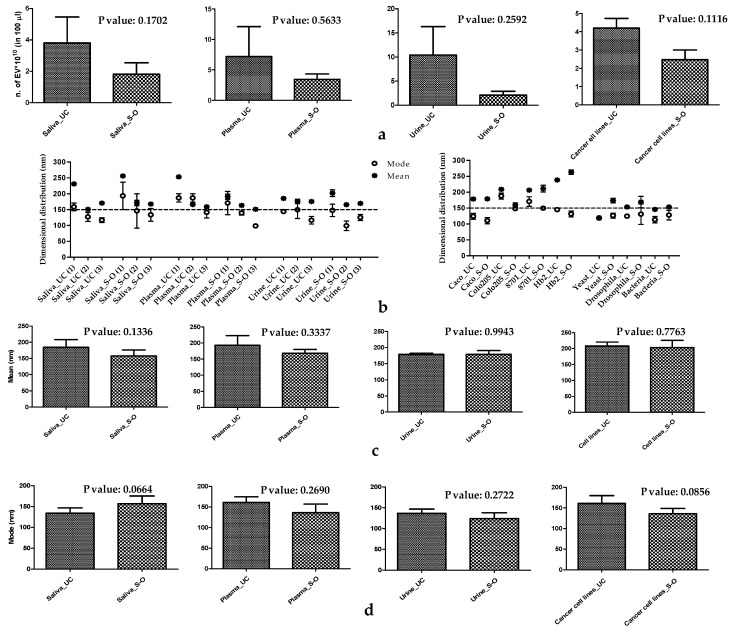
(**a**) Histogram of the number of EVs from biofluids and human cancer cell lines, comparing the S-O and UC isolation methods. (**b**) Scatter plot showing the dimensional distribution of EVs. (**c**) Histogram of the mean values comparing the S-O and UC. (**d**) Histogram of the mode values comparing the S-O and UC isolation methods.

The number of vesicles isolated with both methods, and measured with NTA, varied around 10^8^ per µL, as reported in Table 1. As reported in Figure 1a, the amount of EV derived from samples of cancer cell lines, plasma, saliva and urine shows no statistically significant differences between S-O and UC. The only differences in terms of the number of EVs were relative to the insect and the unicellular organisms, as reported in Table 1. For the EV size characterisation, we report the, as descriptors of the number weighted size distribution, the “mean” of the distribution and the “mode” (the size value that occurs most frequently). For a symmetric monomodal size distribution, the two values coincide. The complete results for each purified sample, expressed as the mean and mode size, are summarised in Table 1 and all NTA information is reported in Supplementary Figures S11–S42. Figure 1b shows, in a graphical presentation of the Table 1 data, that the mean size of the EVs is, with few exceptions, for both methods above 150–200 nm, which is conventionally used as a dimensional cut-off between exosomes and small EVs (sEV) [48,49]. If we analyse the mode values, the EVs isolated from yeast, Drosophila, bacteria, Caco-2, HB2 CM and urine had a value below 150 nm using both isolation methods. EVs isolated from plasma and saliva had mode values between 150 and 200 nm, while those isolated from 8701-BC and Colo 205 cells had the same value over 150 nm, only when they were purified by UC. As shown in Figure 1c,d, there are no significant differences for the mean and the mode between the two methods in all samples reported. DLS was used to further characterise the size values of EVs obtained by both isolation techniques. DLS commercial software provides two classes of methods to assess size distribution: the so-called “cumulant analysis”, which is a robust method that furnishes an intensity-weighted average size, called the “z-average”; and the inversion of a Laplace transformation to reconstruct the whole intensity-weighted size distribution. The number-weighted size distribution can be calculated from the intensity-weighted size distribution, normalising for the vesicle mass as previously described [35]. A representative example of these two approaches is shown in Figure S1c*,* where the number and intensity size distributions coming from DLS are compared with the NTA data in the case of the EVs isolated from HB2 CM by UC. As descriptors of the size distribution that were probed by DLS, we used the intensity-weighted z-average size and the mode of the number weighted size distribution. The values of both descriptors for each purified sample are summarised in Table 1. It should be stressed that these two parameters have different meanings: the z-average is strongly influenced by the presence of large aggregates (as confirmed by values shown in Table 1, which in most cases are well above 150 nm for the samples obtained by means of both UC and S-O), whereas the corresponding number-weighted size distribution is centered on the smaller particles. Therefore, the DLS number distribution clearly improves the agreement between the NTA and DLS data, confirming the prevalence of particles with sizes from 100–150 nm in the sample (data are summarised in Table 1).

**Figure 2:**
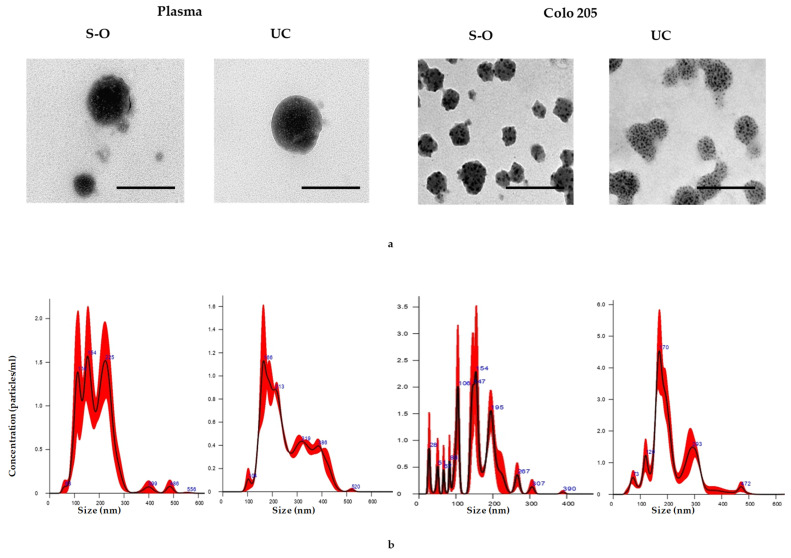
(**a**) Characterisation of the EVs isolated from plasma and Colo205 cells by TEM (Scale bar 100 nm) and (**b**) by NTA.

In order to have a further confirmation of the presence of EVs in the samples obtained at the end of both the isolation protocols, we performed their characterisation by TEM. In Figure 2a,b, the size and distribution of EVs, isolated from plasma and conditioned medium of Colo205 cells utilizing UC and S-O, and measured by TEM and NTA, are shown and, are representative of all other samples. The EVs look roughly spherical and the EVs by UC display a more regular shape than the S-O ones. The size is smaller than that measured by NTA and DLS, in particular for the Colo205-EVs, but it is worth mentioning that under the TEM the EVs are dry, and thus have shrunk and contracted. Furthermore, the characterisation of EV size by NTA, obtained from conditioned medium of yeast cells utilizing both techniques, and their corresponding bin size plots as representative of all other samples analysed, is showed in Figure S1a,b.

### 3.2. Biochemical Characteristics of EVs

The biochemical characterisation of EVs included FCM analysis, Western blot, and the nanoplasmonic colorimetric assay. The FCM allows us to obtain information regarding the positivity of three conventional exosomal markers (tetraspanins CD63 and CD81 and ALIX) as suggested in MISEV2018 guidelines [9], while the nanoplasmonic assay provides information about the protein contamination of the isolated vesicles.

**Figure 3:**
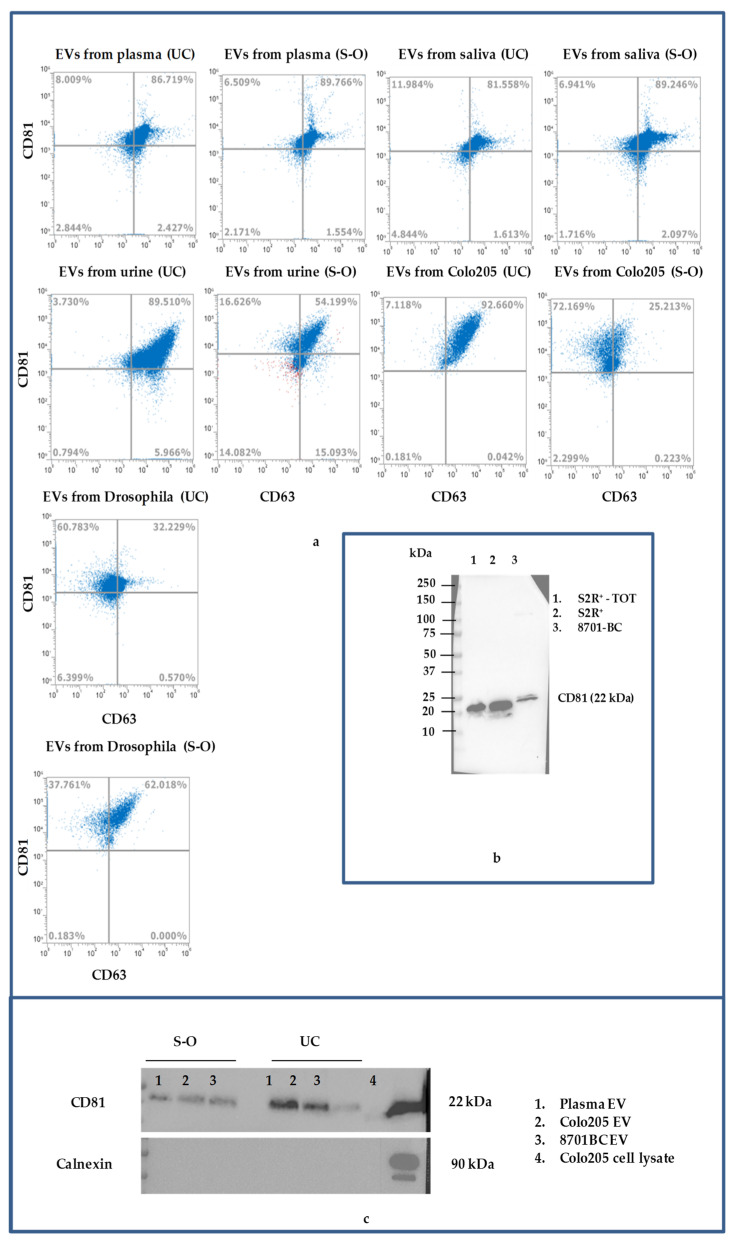
(**a**) Dotplots of CD81 +/CD63+ EVs isolated from biological fluid and cell lines by S-O and UC. (**b**) Electrophoretic bands of CD81 in lysates from Drosophila (total lysate S2R^+^-TOT or clarified to cellular membrane S2R^+^) and 8701-BC cell lines. (TOT: total lysate). (**c**) Electrophoretic bands of CD81 and calnexin in EVs from plasma, Colo205 and 8701BC and in Colo205 cell lysate.

To more precisely compare the UC and S-O methods applied to the different samples included in our study, we carried out a FCM analysis to investigate the presence of the tetraspanins CD63 and CD81, typically expressed on the surface of EVs, and ALIX, a protein of cytoplasmatic origin. Among human fluids, the results obtained for the plasma isolated with both UC and S-O showed that most of the EVs had similar expression levels to the two tetraspannins while an increase (about 20%) in ALIX expresssion was detectable in the S-O samples (Figures S2–S4). Urine and saliva samples showed no significant differences between the pairs of samples obtained with UC and S-O (Figures S5–S8). EVs obtained from human cell lines also showed no differences between the two methods, in addition. In detail, cancer cell lines of mammary origin, 8701-BC (from breast cancer), and the colon cancer cell lines Caco-2 and Colo 205 released EVs that were positive for both CD63 and CD81 (approximately 91 and 52%) and ALIX (approximately 49%), while healthy epithelium HB2 cells released approximately 88, 59 and 56% of EVs that were positive for CD81, CD63 and ALIX, respectively (Figure 3a and Figures S2–S6). Surprisingly, the EVs released by Drosophila cell lines interacted with CD63, CD81 and ALIX human antibodies (Figure 3a and Figure S2), while no positivity was found for the EVs from yeast and bacteria (Figure S9). Irrespective to the isolation method, the 89, 52 and 34% of EVs released by Drosophila cells expressed CD81, CD63 and ALIX, respectively. To the best of our knowledge, such interactions have not been previously demonstrated and would enlarge the range of applications of these antibodies. In this regard, the interaction between human antibody CD81 and Drosophila antigen was also verified and confirmed by Western blot (see Figure 3b).

In order to confirm the expression of CD81 on the EVs we performed a Western blot on the EVs derived from plasma, Colo205 and 8701B C cell lines (Figure 3c and Figure S10), utilizing calnexin as the negative control, which resulted in only the expression of CD81 in Colo205 cells lysate and not in EVs.

**Figure 4:**
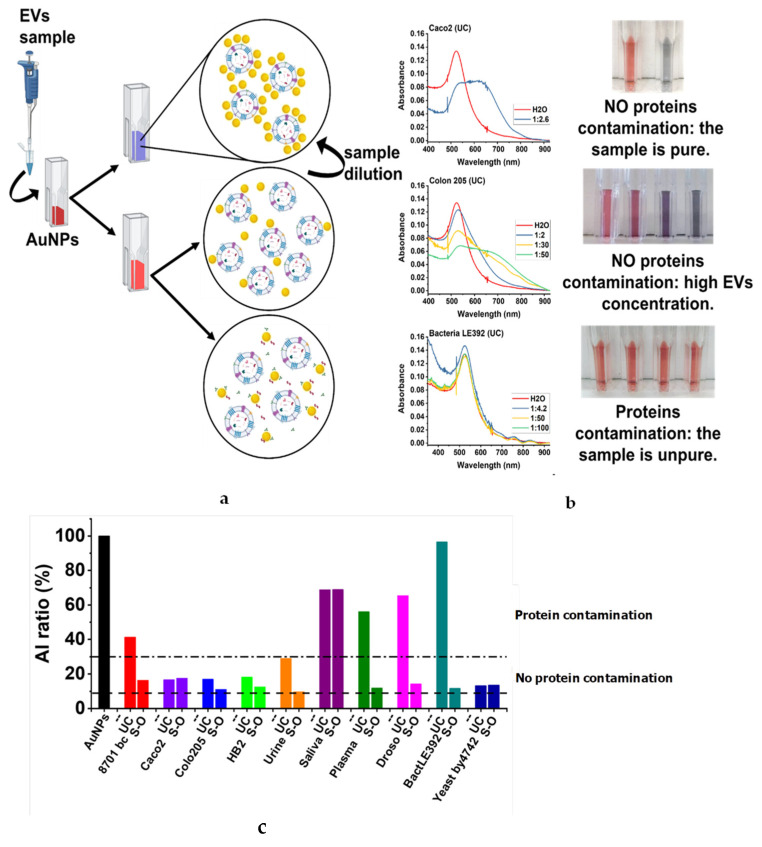
(**a**) Pictorial representation of the three possible scenarios generated by the addition of an EV preparation to an AuNP solution. (**b**) Absorbance spectra obtained for three representative samples. Sample dilutions are reported in the legend of the spectra. (**c**) Aggregation index ratio (AIratio) of pure or contaminated EV samples. For each sample, the maximum dilution prepared was reported. The dashed dotted line represents the threshold below which a properly diluted EV preparation is free of contaminating proteins. The dashed line corresponds to the AIratio obtained in the presence of PBS only.

Customarily, the concentration of proteins in EVs was used as a standard method for their characterisation. However, in the case of protein contamination, this results in an overestimation of the EV concentration [48]. Furthermore, protein contamination could adversely affect EV use and analysis. Thus, vesicle purity is crucial in EV studies and in the choice of the isolation method used. We addressed this point using the CONAN assay for probing the protein contaminants and EV purity [44,50]. When EVs are added to AuNPs, the solution colour changes from pink to blue if the EVs are pure. Conversely, if the concentration of EVs is too high or if they are contaminated with proteins, the solution does not change colour or turns pink (Figure 4a). The visible spectra and pictures of the corresponding samples obtained for the three different possible scenarios are shown in Figure 3b. In Figure 4b, qualitative results obtained with all samples are shown based on the colour of the solution. Several dilutions of the same sample were prepared, and if none of the dilutions turned blue, the sample was considered contaminated.

The degree of AuNP aggregation was quantified through the AI_ratio_ parameter (see Materials and Methods).

In Figure 4c, the AI_ratio_ of all EV samples is summarised, and in particular, the values of the last dilution are shown. A 100% AI_ratio_ corresponds to a monodispersed sample of AuNPs. The dashed-dotted line corresponds to the AI_ratio_ below which the AuNPs start to aggregate in the presence of only EVs. If the tested sample’s AI_ratio_ is above the threshold, it can be deduced that the EV preparation is contaminated by proteins. Figure 4c shows as S-O is very effective in protein depletion respect to UC. In fact, all types of EV isolated by means of S-O, with the exception of EVs from saliva, were free of proteins. Otherwise, UC was effective in protein depletion on only half of the samples: EVs from saliva, plasma, 8701-BC, Drosophila and bacteria were contaminated by proteins while preparations from urine, Caco-2, HB2, Colo 205 and yeast were pure.

## 4. Discussion

EVs are naturally generated from different subcellular compartments. Composed of a phospholipid bilayer that is decorated with polysaccharides and membrane proteins, EVs can enclose diverse cargoes, including proteins, RNAs, DNAs and lipids. In addition to their importance as mediators in intercellular communication, EVs represent a promising source of novel biomarkers in the diagnosis and prognosis of diseases [24], as well as a new emerging therapeutic tool [51].

To elucidate EV functionality and their potential for medical prognoses, diagnoses and pharmaceutical uses, suitable isolation protocols that are able to provide quick and accessible procedures for EV isolation, and possibly yield a pure sample, are needed. Most papers have compared many EV isolation approaches, but to date, identifying an adequate and universal method of isolation remains the main topic of concern [23]. Method versatility and EV purity are the two main issues discussed in the available EV isolation protocols.

In 2015 Sáenz-Cuestaet al. [1] compared UC with S-O methods and three other EV isolation methods to identify a protocol suitable for application in a hospital setting. However, they used the standard UC protocol, which was modified (without the second centrifuge step at 110,000× *g*). The study was performed on EVs derived only from blood and urine, and they reported no significant differences in the EV concentrations between those yielded by the UC and S-O methods.

The MISEV2018 guidelines [9] state that the choice of the EV isolation protocol must be made based on the applications and use of the vesicles themselves, and that there is currently no optimal isolation method. They also state that the separation of EVs from non-vesicular entities cannot be achieved by common centrifugation protocols or commercial kits. Thus for the first time, we tested the UC and S-O methods on a large and diverse sample collected with the aim of evaluating the isolation efficiency in terms of the quantity, size and purity of the isolated samples. On the basis of our results, we wished to identify the methodology that would allow us to obtain pure samples to be used in different fields of application, from the research lab to the clinic.

EVs possess extensive biomedical potential. However, what hinders their characterisation and application is the ability to obtain pure material. Towards physical and biochemical analysis, we compared the S-O protocol with UC, which is the most widely used method. The possible presence of contaminating proteins was assessed through the CONAN assay. The study was carried out both on conditioned media of human healthy cells, cancer cell lines and three bodily fluids (plasma, urine, and saliva), as well as conditioned media of insects, bacteria and yeast cell cultures.

Concerning the physical characterisation of isolated EVs, both isolation protocols which were applied allowed us to obtain EVs with a diameter that is compatible with that of exosomes and sEV. Considering both the mean value obtained by NTA and the z-average obtained by DLS, the two isolation methods, in almost all cases, led to vesicles larger than 150 nm. But if we consider that the DLS z-average is strongly influenced by the presence of large aggregates and we focus on the means obtained with the NTA, we not only observe more homogeneous measures but also no significant differences in terms of the means between the two isolation methods in all samples analysed. The comparison of z-average values (by DLS) with the mean values (by NTA) highlights that the measurements made with NTA show a homogeneity of the isolated vesicles, higher than those made with DLS. By analysing the mode values obtained with both NTA and DLS, a different scenario arises. In particular, in all samples, by means of both separation techniques, vesicles with diameters smaller than 200 nm were obtained, and the comparison between the mode values obtained with both techniques showed similar results, allowing us to reach the same conclusions. Thus, independently from the results obtained with NTA and DLS, UC and S-O allowed us to obtain samples of EVs showing superimposable physical characteristics.

Another important indicator for the efficiency of the two methods of EV isolation comes from the comparison of FCM results. All human samples show a significant and comparable positivity to CD63, CD81 and ALIX antibodies, which are recognised as exosomal markers in MISEV2018 guidelines [9,52,53]. This allows us to state how the two methods contribute in a comparable way to enriching the precipitates in exosome-like vesicles. These data are also supported by their physical characterisation. Although FCM was also conducted on non-human EV samples, for use as a negative control in our experiment, surprisingly, in *Drosophila*, we recorded positive events which were confirmed by Western blot analysis, which showed that the human antibodies CD63, CD81 and ALIX recognise proteins at the same height as that expected in a human cell lysate. This result expands the available toolbox to investigate the biogenesis and biological functionality of EVs in an excellent and evergreen model organism such as *Drosophila* [54].

Regarding protein contamination, the coprecipitation of proteins or other contaminants is one of the biggest limitations in the study of EVs and their application in the biomedical field. In our work, we were interested in determining whether the provided samples, which were purified with two different protocols, were contaminated with protein, to assess the efficacy of both methods in terms of protein depletion. We addressed this point utilizing a cost-effective and fast nanoplasmonic colorimetric assay [44]. The assay provides crucial information regarding the effectiveness and protein depletion ability of S-O with respect to UC. The S-O approach allows pure EV preparation, except in the cases of saliva samples.

The protein contamination of EVs from saliva samples could be justified, considering that saliva is an aqueous dispersion in which the presence of mucins and high molecular weight proteins makes it highly viscous [52] and therefore difficult to manipulate, as already reported when utilizing other methods of EV isolation from saliva, by which samples rich in free proteins were obtained [53].

In conclusion, the S-O protocol has been demonstrated to be a good and versatile method of isolating EVs from various samples. Indeed, it is able to provide preparations containing vesicles with a size of 40–200 nm. Moreover, it is more effective in the depletion of contaminating proteins, which may lead to significant advantages in EV characterisation and reduce mistakes in understanding EV functions and applications. Finally, taken together, our results show that even if the yield in terms of quantity of the EVs obtained is better with UC isolation in terms of the costs, isolation speed and purity of the samples obtained, the S-O method proved to be a very effective alternative to UC, which is considered the gold standard for isolation of EVs to date.

## Figures and Tables

**Table 1:** Concentration and size distribution of EVs of different origins by NTA and DLS.

Sample	NTA			DLS	
Human Cell Lines	n. of EVs ± S.D./μL	Mean ± S.D. (d. nm)	Mode ± S.D. (d. nm)	Z-Average ± S.D.	Mode by Number (FWHH *)
Caco-2_UC	(5.2 ± 1.3) × 10⁸	178.6 ± 9.5	124.2 ± 9.1	440.9 ± 27.7	122 (80)
Caco-2_S-O	(3.1 ± 0.2) × 10⁸	179 ± 5	110.5 ± 9.9	552.6 ± 28.7	100 (50)
Colo 205_UC	(4.0 ± 0.4) × 10⁸	209 ± 8	187.9 ± 9.5	374 ± 95	106 (56)
Colo 205_S-O	(1.4 ± 0.1) × 10⁸	159.8 ± 11.2	148.2 ± 3.9	944.5 ± 76.2	142 (50)
8701-BC_UC	(3.4 ± 0.2) × 10⁸	206.7 ± 4.8	170.6 ± 15.1	663.4 ± 35.6	122 (66)
8701-BC_S-O	(2.9 ± 0.6) × 10⁸	211.4 ± 18.3	149.7 ± 3.8	267.1 ± 26.8	113 (58)
HB2_UC	(6.8 ± 0.4) × 10⁸	238.5 ± 2.2	144.9 ± 1.3	547.3 ± 32.5	130 (40)
HB2_S-O	(3.3 ± 0.1) × 10⁸	263.1 ± 12.1	131.3 ± 9.3	381.1 ± 3.7	143 (64)
Body Fluids					
Saliva_UC (1)	(6.7 ± 0.3) × 10⁸	231.1 ± 3.1	158.7 ± 12.2	195 ± 16	143 (66)
Saliva_UC (2)	(9.80 ± 0.9) × 10^7^	150.9 ± 6.2	127.0 ± 14.1	212 ± 32	142 (62)
Saliva_UC (3)	(3.74 ± 0.2) × 10^8^	170.5 ± 3.0	116.9 ± 7.4	237 ± 40	105 (61)
Saliva_S-O (1)	(2.9 ± 0.4) × 10⁸	256.1 ± 3.9	193.4 ± 43.2	255 ± 14	121 (54)
Saliva_S-O (2)	(4.10 ± 0.2) × 10^7^	170.8 ± 14.8	146.0 ± 54.1	574 ± 44	134 (47)
Saliva_S-O (3)	(2.13 ± 0.2) × 10^8^	167.6 ± 3.3	133.7 ± 20.1	1022 ± 200	165 (45)
Plasma_UC (1)	(1.7 ± 0.1) × 10⁹	253.4 ± 7.3	186.9 ± 13.1	96.7 ± 8.3	68 (35)
Plasma_UC (2)	(2.71 ± 0.2) × 10^8^	166.0 ± 2.6	141.3 ± 17.5	132 ± 9	93 (47)
Plasma_UC (3)	(1.93 ± 0.1) × 10^8^	158.8 ± 11.1	155.8 ± 35.6	147 ± 12	81 (43)
Plasma_S-O (1)	(2.3 ± 0.3) × 10⁸	190.4 ± 14.2	170.8 ± 36.6	not suitable	not suitable
Plasma_S-O(2)	(5.24 ± 1.1) × 10^8^	163.3 ± 3.7	139.3 ± 6.6	268 ± 70	106 (43)
Plasma_S-O (3)	(2.82 ± 0.3) × 10^8^	151.2 ± 2.2	98.5 ± 3.0	850 ± 350	61 (35)
Urine_UC (1)	(2.2 ± 0.1) × 10⁹	185.4 ± 2.8	143.8 ± 4.0	144.4 ± 0.7	60 (22)
Urine_UC (2)	(6.65 ± 0.1) × 10^8^	175.7 ± 1.1	116.7 ± 12.0	247 ± 9	105 (45)
Urine_UC (3)	(2.59 ± 0.2) × 10^8^	176.0 ± 12.2	149.7 ± 27.8	275 ± 14	108 (42)
Urine_S-O (1)	(3.5 ± 0.1) × 10⁸	202.0 ± 19	147.6 ± 19.5	332.5 ± 48.9	152 (60)
Urine_S-O (2)	(6.49 ± 0.6) × 10^7^	165.6 ± 7.5	99.0 ± 15.1	756 ± 110	106 (36)
Urine_S-O (3)	(2.13 ± 0.2) × 10^8^	169.7 ± 2.9	125.3 ± 9.5	1650 ± 215	174 (40)
Cell Cultures Models					
Yeast_UC	(8.8 ± 0.5) × 10⁸	119.9 ± 1.1	118.2 ± 4.4	187 ± 1	98 (50)
Yeast_S-O	(2.8 ± 0.5) × 10⁸	173.8 ± 12.6	126.4 ± 7.7	not suitable	not suitable
Drosophila_UC	(3.8 ± 0.6) × 10⁸	153.7 ± 0.8	124.4 ± 5.4	188.5 ± 88.5	54 (22)
Drosophila_S-O	(1.3 ± 0.1) × 10⁸	169.1 ± 31.1	130.7 ± 32.2	not suitable	not suitable
Bacteria_UC	(8.3 ± 5.9) × 10⁸	145.8 ± 9.3	113.4 ± 10.1	379.5 ± 70.1	84 (40)
Bacteria_S-O	(2.9 ± 1.5) × 10⁸	153.5 ± 5	127.9 ± 15.3	not suitable	not suitable

* FWHH (full width at half height).

## Supplementary Materials

The following are available online at https://www.mdpi.com/article/10.3390/biom11121857/s1, Figure S1: (a) NTA histograms with confidence interval in red (S.D.) reporting the concentration and specific particle size of EVs from yeast isolated by S-O and UC. (b) Bin size-plots of EVs from yeast isolated by S-O and UC and analysed by NTA. The bin width of NTA distributions is 1. (c) Representative histogram comparing the DLS and NTA analysis results for EVs isolated by UC from HB2-conditioned medium. Figure S2: (a) Dot-plots of unstained EVs isolated from plasma 1, Colo205 and Drosophila by S-O and UC. Figure S3: Dot-plots of CD81+/CD63+/ALIX+ EVs isolated from plasma 2/3, Caco-2 and 8701-BC by S-O and UC. Figure S4: Dot-plots of unstained EVs isolated from plasma 2/3, Caco-2 and 8701-BC by S-O and UC. Figure S5: Dot-plots of CD81+/CD63+/ALIX+ EVs isolated from urine 1–3 and HB2 cells by S-O and UC. Figure S6: Dot-plots of unstained EVs isolated from urine 1–3 and HB2 cells by S-O and UC. Figure S7: Dot-plots of CD81+/CD63+/ALIX+ EVs isolated from saliva 1–3 by S-O and UC. Figure S8: Dot-plots of unstained EVs isolated from saliva 1–3 by S-O and UC. Figure S9: Dot-plots of CD81+/CD63+ and unstained EVs isolated from Yeast and Bacteria by S-O and UC. Figure S10: Electrophoretic bands of CD81 and Calnexin in lysates from EVs (derived by plasma, Colo205 and 8701BC) and Colo205 cell line, and relative stain free gel. Figures S11–S42: EVs NTA informations. Table S1: EVs isolation characteristics. Supplementary File S1: EVs analysis by Flow Cytometry.
