# Supplementary material for: Salting-Out Approach Is Worthy of Comparison with Ultracentrifugation for Extracellular Vesicle Isolation from Tumor and Healthy Models
Source: Biomolecules. 2021 Dec 10;11(12):1857. doi: 10.3390/biom11121857 (PMC8699204; doi:10.3390/biom11121857)
Supplement: Supplementary file 1 [file biomolecules-11-01857-s001.zip › Figure S11.pdf]

NANOSIGHT

EV_plasma_S-O_pz2 2021-09-02 16-20-28

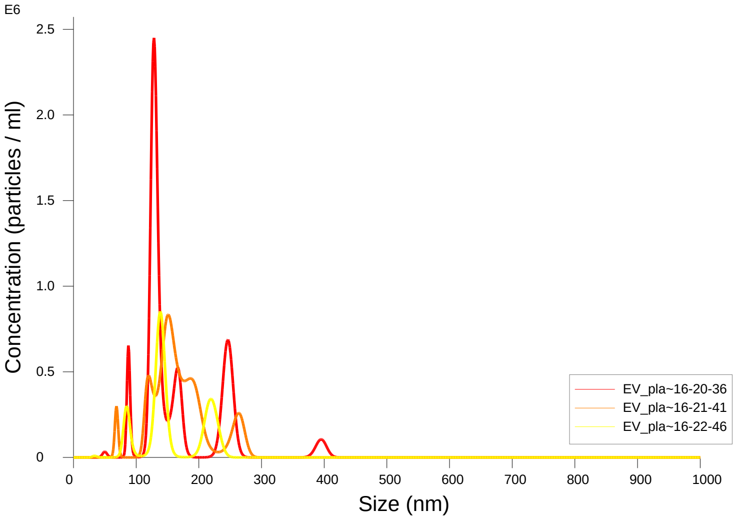

FTLA Concentration / Size graph for Experiment:
EV_plasma_S-O_pz2 2021-09-02 16-20-28

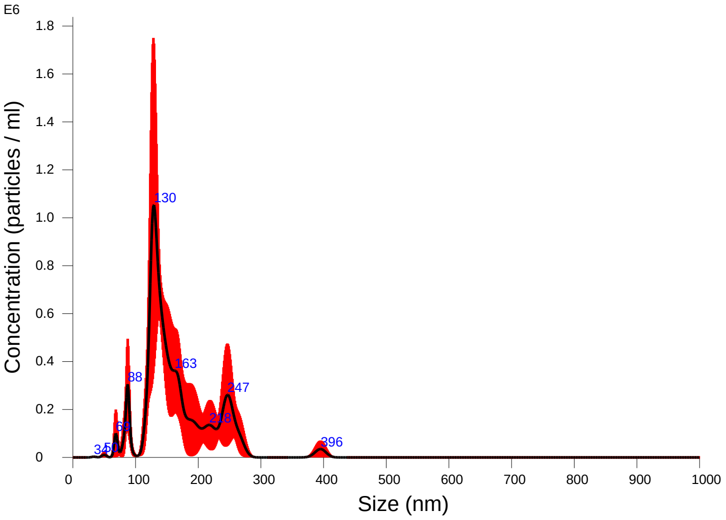

Averaged FTLA Concentration / Size for Experiment:
EV_plasma_S-O_pz2 2021-09-02 16-20-28
Error bars indicate + / -1 standard error of the mean

Included Files

EV_plasma_S-O_pz2 2021-09-02 16-20-36
EV_plasma_S-O_pz2 2021-09-02 16-21-41
EV_plasma_S-O_pz2 2021-09-02 16-22-46

Details

NTA Version:	NTA 3.2 Dev Build 3.2.16
Script Used:	SOP Standard Measurement 04-20-28PM 02~
Time Captured:	16:20:28 02/09/2021
Operator:	simo
Pre-treatment:	
Sample Name:	EV_plasma_S-O_pz2
Diluent:	
Remarks:	1:1000

Capture Settings

Camera Type:	sCMOS
Laser Type:	Green
Camera Level:	14
Slider Shutter:	1259
Slider Gain:	366
FPS	25.0
Number of Frames:	1498
Temperature:	26.3 ^{o}C
Viscosity:	(Water) 0.861 - 0.863 cP
Dilution factor:	Dilution not recorded
Syringe Pump Speed:	50

Analysis Settings

Detect Threshold:	5
Blur Size:	Auto
Max Jump Distance:	Auto: 14.0 - 16.9 pix

Results

Stats: Merged Data

Mean:	164.8 nm
Mode:	129.1 nm
SD:	55.6 nm
D10:	118.2 nm
D50:	146.2 nm
D90:	245.4 nm

Stats: Mean +/- Standard Error

Mean:	163.3 +/- 3.7 nm
Mode:	139.3 +/- 6.6 nm
SD:	52.6 +/- 6.5 nm
D10:	108.8 +/- 10.6 nm
D50:	144.7 +/- 7.5 nm
D90:	240.5 +/- 8.5 nm
Concentration:	5.24e+007 +/- 1.18e+007 particles/ml
	2.7 +/- 0.6 particles/frame
	3.3 +/- 0.8 centres/frame

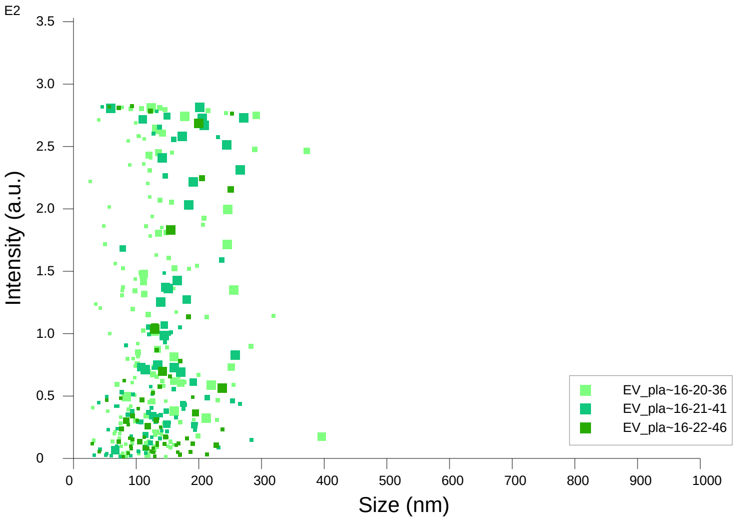

Intensity / Size graph for Experiment:
EV_plasma_S-O_pz2 2021-09-02 16-20-28

Script Used: (Full Text):

SOP Standard Measurement 04-20-28PM 02Sep2021.txt
