# Supplementary material for: Salting-Out Approach Is Worthy of Comparison with Ultracentrifugation for Extracellular Vesicle Isolation from Tumor and Healthy Models
Source: Biomolecules. 2021 Dec 10;11(12):1857. doi: 10.3390/biom11121857 (PMC8699204; doi:10.3390/biom11121857)
Supplement: Supplementary file 1 [file biomolecules-11-01857-s001.zip › Figure S12.pdf]

NANOSIGHT

EV_plasma_UC_pz2 2021-09-02 13-10-09

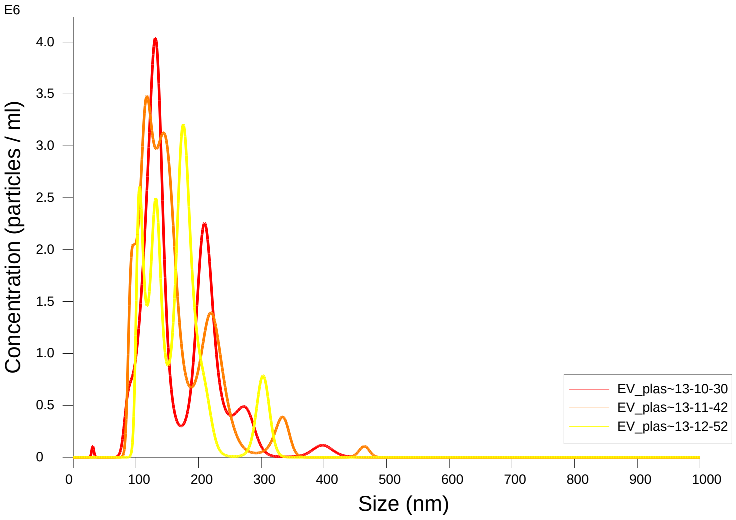

FTLA Concentration / Size graph for Experiment:
EV_plasma_UC_pz2 2021-09-02 13-10-09

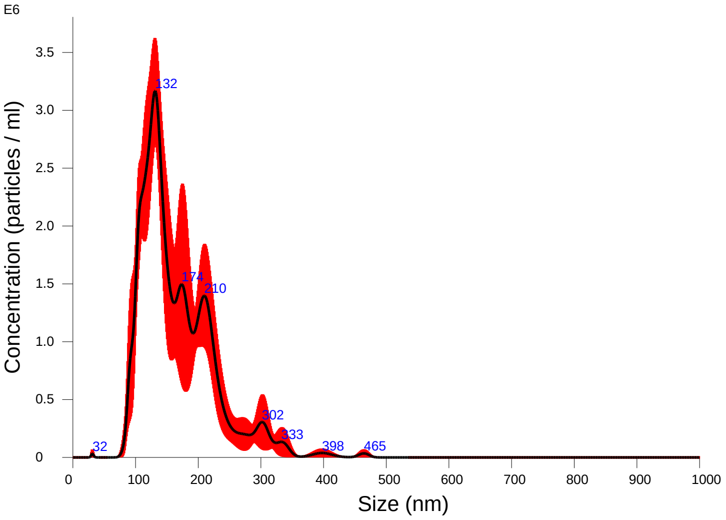

Averaged FTLA Concentration / Size for Experiment:
EV_plasma_UC_pz2 2021-09-02 13-10-09
Error bars indicate + / -1 standard error of the mean

Included Files

EV_plasma_UC_pz2 2021-09-02 13-10-30
EV_plasma_UC_pz2 2021-09-02 13-11-42
EV_plasma_UC_pz2 2021-09-02 13-12-52

Details

NTA Version:	NTA 3.2 Dev Build 3.2.16
Script Used:	SOP Standard Measurement 01-10-09PM 02~
Time Captured:	13:10:09 02/09/2021
Operator:	simo
Pre-treatment:	
Sample Name:	EV_plasma_UC_pz2
Diluent:	
Remarks:	1:1000

Capture Settings

Camera Type:	sCMOS
Laser Type:	Green
Camera Level:	13
Slider Shutter:	1232
Slider Gain:	219
FPS	25.0
Number of Frames:	1498
Temperature:	24.2 - 24.2 ^{o}C
Viscosity:	(Water) 0.904 - 0.906 cP
Dilution factor:	Dilution not recorded
Syringe Pump Speed:	50

Analysis Settings

Detect Threshold:	5
Blur Size:	Auto
Max Jump Distance:	Auto: 11.9 - 12.6 pix

Results

Stats: Merged Data

Mean:	165.6 nm
Mode:	131.2 nm
SD:	58.8 nm
D10:	105.7 nm
D50:	147.5 nm
D90:	235.1 nm

Stats: Mean +/- Standard Error

Mean:	166.0 +/- 2.6 nm
Mode:	141.3 +/- 17.5 nm
SD:	58.2 +/- 2.7 nm
D10:	106.0 +/- 2.1 nm
D50:	150.3 +/- 7.3 nm
D90:	232.4 +/- 7.0 nm
Concentration:	2.71e+008 +/- 2.39e+007 particles/ml
	13.8 +/- 1.2 particles/frame
	15.7 +/- 1.5 centres/frame

EV_plasma_UC_pz2 2021-09-02 13-10-09

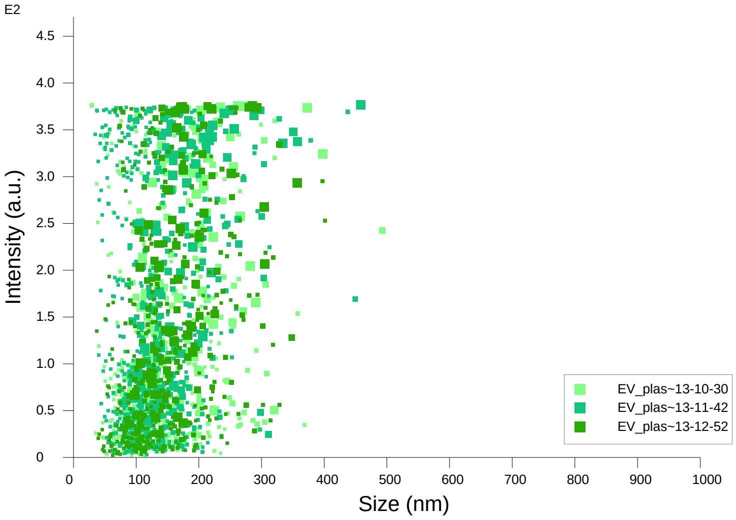

Intensity / Size graph for Experiment:
EV_plasma_UC_pz2 2021-09-02 13-10-09

Script Used: (Full Text):

SOP Standard Measurement 01-10-09PM 02Sep2021.txt
