# Supplementary material for: Salting-Out Approach Is Worthy of Comparison with Ultracentrifugation for Extracellular Vesicle Isolation from Tumor and Healthy Models
Source: Biomolecules. 2021 Dec 10;11(12):1857. doi: 10.3390/biom11121857 (PMC8699204; doi:10.3390/biom11121857)
Supplement: Supplementary file 1 [file biomolecules-11-01857-s001.zip › Figure S13.pdf]

NANOSIGHT

EV_PLASMA_S-O_PZ3 2021-09-03 11-53-29

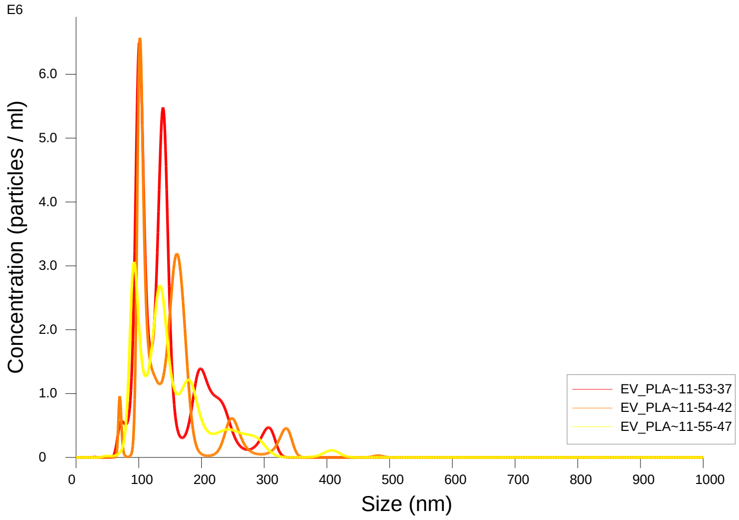

FTLA Concentration / Size graph for Experiment:
EV_PLASMA_S-O_PZ3 2021-09-03 11-53-29

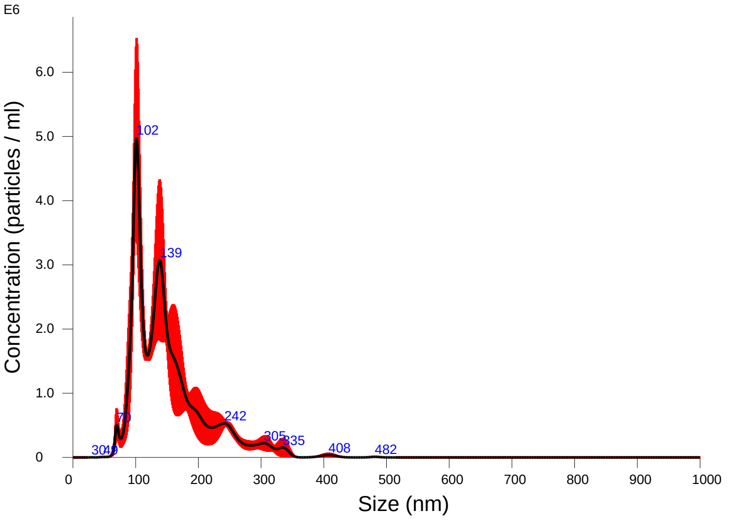

Averaged FTLA Concentration / Size for Experiment:
EV_PLASMA_S-O_PZ3 2021-09-03 11-53-29
Error bars indicate + / -1 standard error of the mean

Included Files

EV_PLASMA_S-O_PZ3 2021-09-03 11-53-37
EV_PLASMA_S-O_PZ3 2021-09-03 11-54-42
EV_PLASMA_S-O_PZ3 2021-09-03 11-55-47

Details

NTA Version:	NTA 3.2 Dev Build 3.2.16
Script Used:	SOP Standard Measurement 11-53-29AM 03~
Time Captured:	11:53:29 03/09/2021
Operator:	simo
Pre-treatment:	
Sample Name:	EV_PLASMA_S-O_PZ3
Diluent:	
Remarks:	1:1000

Capture Settings

Camera Type:	sCMOS
Laser Type:	Green
Camera Level:	14
Slider Shutter:	1259
Slider Gain:	366
FPS	25.0
Number of Frames:	1498
Temperature:	24.5 - 24.6 ^{o}C
Viscosity:	(Water) 0.897 - 0.899 cP
Dilution factor:	Dilution not recorded
Syringe Pump Speed:	50

Analysis Settings

Detect Threshold:	5
Blur Size:	Auto
Max Jump Distance:	Auto: 12.8 - 13.3 pix

Results

Stats: Merged Data

Mean:	150.7 nm
Mode:	101.3 nm
SD:	59.2 nm
D10:	95.0 nm
D50:	135.9 nm
D90:	236.7 nm

Stats: Mean +/- Standard Error

Mean:	151.2 +/- 2.2 nm
Mode:	98.5 +/- 3.0 nm
SD:	59.6 +/- 2.5 nm
D10:	94.6 +/- 2.3 nm
D50:	137.6 +/- 2.5 nm
D90:	239.4 +/- 6.3 nm
Concentration:	2.82e+008 +/- 3.31e+007 particles/ml
	14.3 +/- 1.7 particles/frame
	18.4 +/- 3.7 centres/frame

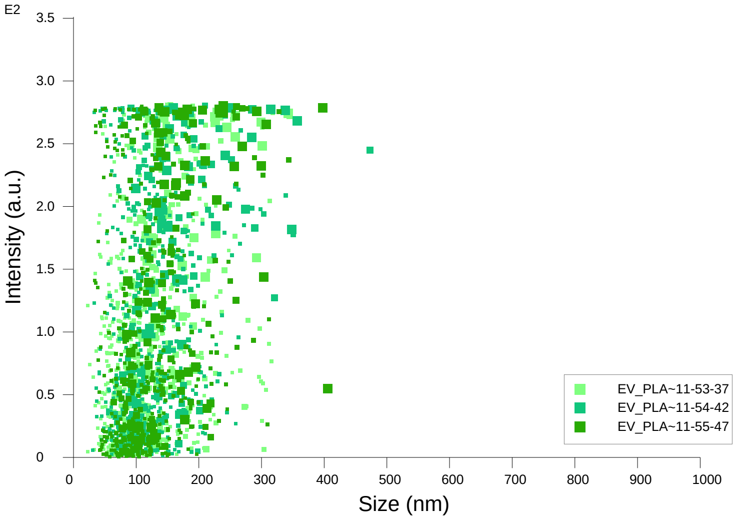

Intensity / Size graph for Experiment:
EV_PLASMA_S-O_PZ3 2021-09-03 11-53-29

Script Used: (Full Text):

SOP Standard Measurement 11-53-29AM 03Sep2021.txt
