# Supplementary material for: Salting-Out Approach Is Worthy of Comparison with Ultracentrifugation for Extracellular Vesicle Isolation from Tumor and Healthy Models
Source: Biomolecules. 2021 Dec 10;11(12):1857. doi: 10.3390/biom11121857 (PMC8699204; doi:10.3390/biom11121857)
Supplement: Supplementary file 1 [file biomolecules-11-01857-s001.zip › Figure S14.pdf]

NANOSIGHT

EV_PLASMA_ULTRA_PZ3 2021-09-03 12-01-47

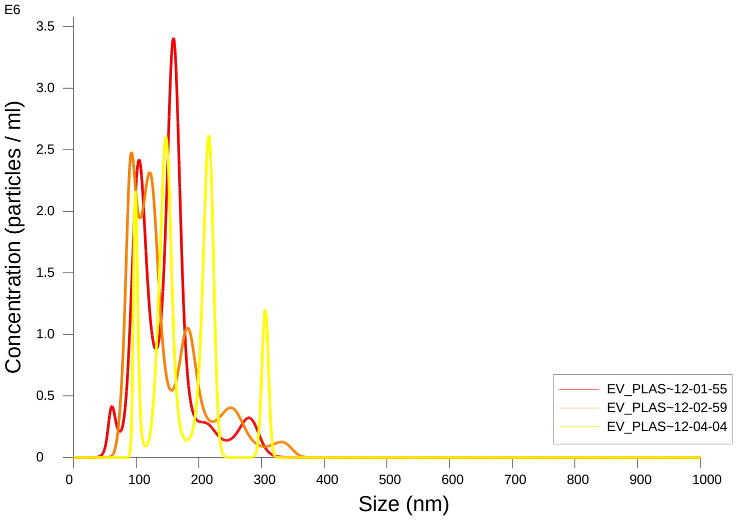

FTLA Concentration / Size graph for Experiment:
EV_PLASMA_ULTRA_PZ3 2021-09-03 12-01-47

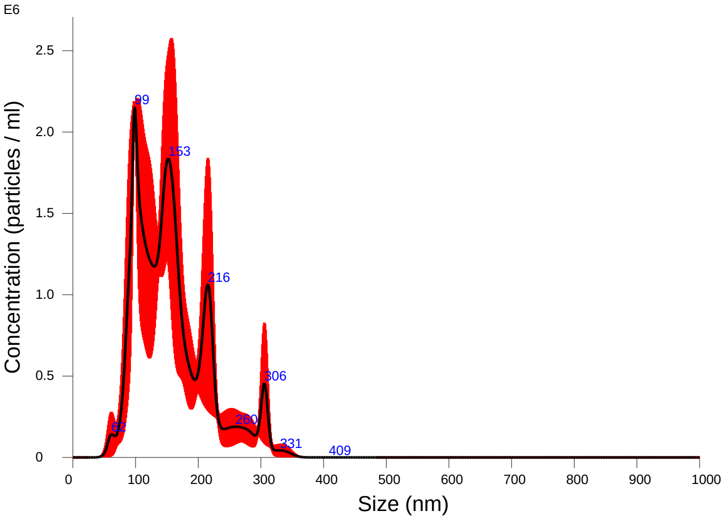

Averaged FTLA Concentration / Size for Experiment:
EV_PLASMA_ULTRA_PZ3 2021-09-03 12-01-47
Error bars indicate + / -1 standard error of the mean

Included Files

EV_PLASMA_ULTRA_PZ3 2021-09-03 12-01-55
EV_PLASMA_ULTRA_PZ3 2021-09-03 12-02-59
EV_PLASMA_ULTRA_PZ3 2021-09-03 12-04-04

Details

NTA Version:	NTA 3.2 Dev Build 3.2.16
Script Used:	SOP Standard Measurement 12-01-47PM 03~
Time Captured:	12:01:47 03/09/2021
Operator:	simo
Pre-treatment:	
Sample Name:	EV_PLASMA_ULTRA_PZ3
Diluent:	
Remarks:	1:1000

Capture Settings

Camera Type:	sCMOS
Laser Type:	Green
Camera Level:	14
Slider Shutter:	1259
Slider Gain:	366
FPS	25.0
Number of Frames:	1498
Temperature:	24.7 - 24.9 ^{o}C
Viscosity:	(Water) 0.892 - 0.894 cP
Dilution factor:	Dilution not recorded
Syringe Pump Speed:	50

Analysis Settings

Detect Threshold:	5
Blur Size:	Auto
Max Jump Distance:	Auto: 11.5 - 16.5 pix

Results

Stats: Merged Data

Mean:	156.8 nm
Mode:	98.7 nm
SD:	57.6 nm
D10:	93.9 nm
D50:	146.7 nm
D90:	229.4 nm

Stats: Mean +/- Standard Error

Mean:	158.8 +/- 11.1 nm
Mode:	155.8 +/- 35.6 nm
SD:	55.7 +/- 3.1 nm
D10:	94.6 +/- 4.0 nm
D50:	145.0 +/- 10.1 nm
D90:	246.1 +/- 23.5 nm
Concentration:	1.93e+008 +/- 1.77e+007 particles/ml
	9.8 +/- 0.9 particles/frame
	11.5 +/- 1.1 centres/frame

NANOSIGHT

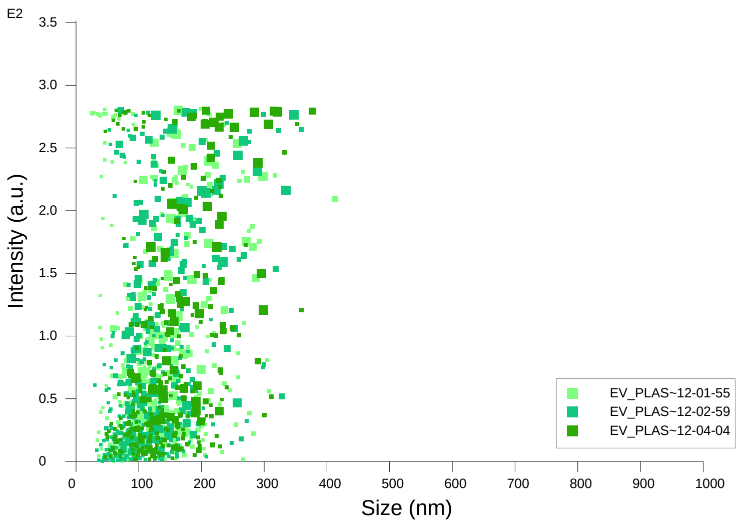

Intensity / Size graph for Experiment:
EV_PLASMA_ULTRA_PZ3 2021-09-03 12-01-47

Script Used: (Full Text):

SOP Standard Measurement 12-01-47PM 03Sep2021.txt
