# Supplementary material for: Salting-Out Approach Is Worthy of Comparison with Ultracentrifugation for Extracellular Vesicle Isolation from Tumor and Healthy Models
Source: Biomolecules. 2021 Dec 10;11(12):1857. doi: 10.3390/biom11121857 (PMC8699204; doi:10.3390/biom11121857)
Supplement: Supplementary file 1 [file biomolecules-11-01857-s001.zip › Figure S15.pdf]

NANOSIGHT

Plasma_NaAc 2019-11-20 11-19-01

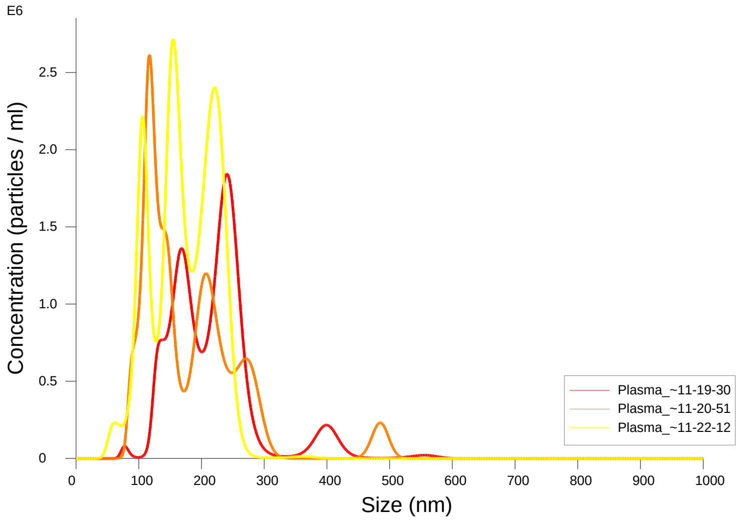

FTLA Concentration / Size graph for Experiment:
Plasma_NaAc 2019-11-20 11-19-01

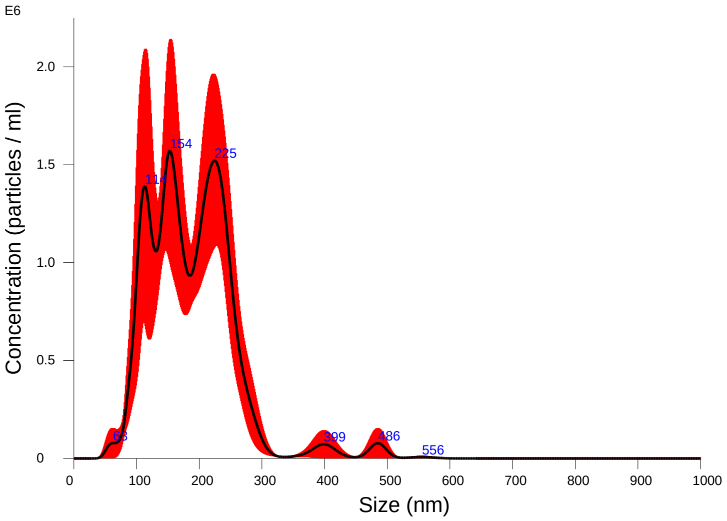

Averaged FTLA Concentration / Size for Experiment:
Plasma_NaAc 2019-11-20 11-19-01
Error bars indicate + / -1 standard error of the mean

Included Files

Plasma_NaAc 2019-11-20 11-19-30
Plasma_NaAc 2019-11-20 11-20-51
Plasma_NaAc 2019-11-20 11-22-12

Details

NTA Version:	NTA 3.2 Dev Build 3.2.16
Script Used:	SOP Standard Measurement 11-19-01AM 20~
Time Captured:	11:19:01 20/11/2019
Operator:	Antonio
Pre-treatment:	
Sample Name:	Plasma_NaAc
Diluent:	
Remarks:	

Capture Settings

Camera Type:	sCMOS
Laser Type:	Green
Camera Level:	13
Slider Shutter:	1232
Slider Gain:	219
FPS	25.0
Number of Frames:	1498
Temperature:	23.0 ^{o}C
Viscosity:	(Water) 0.9 cP
Dilution factor:	Dilution not recorded

Analysis Settings

Detect Threshold:	5
Blur Size:	Auto
Max Jump Distance:	Auto: 11.3 - 13.1 pix

Results

Stats: Merged Data

Mean:	187.6 nm
Mode:	153.0 nm
SD:	69.6 nm
D10:	108.0 nm
D50:	180.3 nm
D90:	255.9 nm

Stats: Mean +/- Standard Error

Mean:	190.9 +/- 14.2 nm
Mode:	170.8 +/- 36.6 nm
SD:	67.2 +/- 9.4 nm
D10:	116.9 +/- 13.0 nm
D50:	180.6 +/- 19.0 nm
D90:	259.5 +/- 13.5 nm
Concentration:	2.30e+008 +/- 3.05e+007 particles/ml
	11.7 +/- 1.5 particles/frame
	20.1 +/- 5.0 centres/frame

Plasma_NaAc 2019-11-20 11-19-01

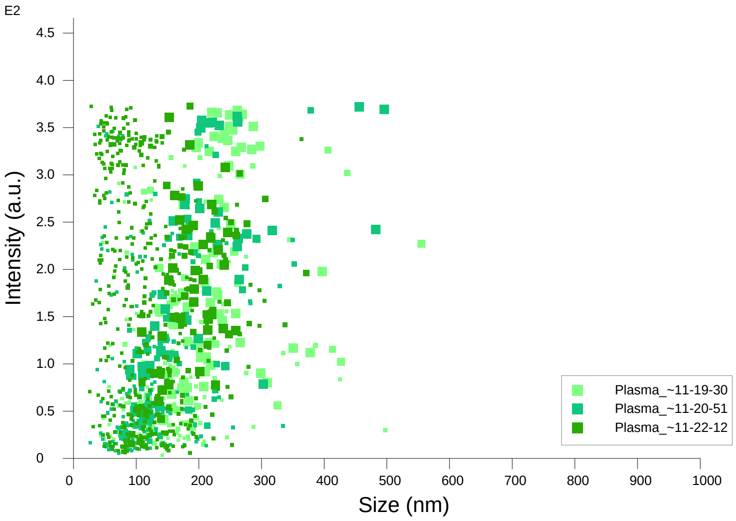

Intensity / Size graph for Experiment:
Plasma_NaAc 2019-11-20 11-19-01

Script Used: (Full Text):

SOP Standard Measurement 11-19-01AM 20Nov2019.txt
