# Supplementary material for: Salting-Out Approach Is Worthy of Comparison with Ultracentrifugation for Extracellular Vesicle Isolation from Tumor and Healthy Models
Source: Biomolecules. 2021 Dec 10;11(12):1857. doi: 10.3390/biom11121857 (PMC8699204; doi:10.3390/biom11121857)
Supplement: Supplementary file 1 [file biomolecules-11-01857-s001.zip › Figure S16.pdf]

NANOSIGHT

Plasma_U 2019-11-20 12-51-11

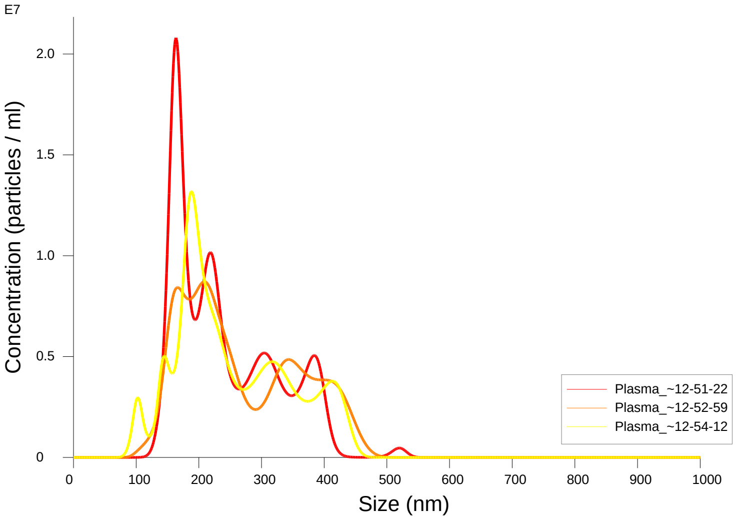

FTLA Concentration / Size graph for Experiment:
Plasma_U 2019-11-20 12-51-11

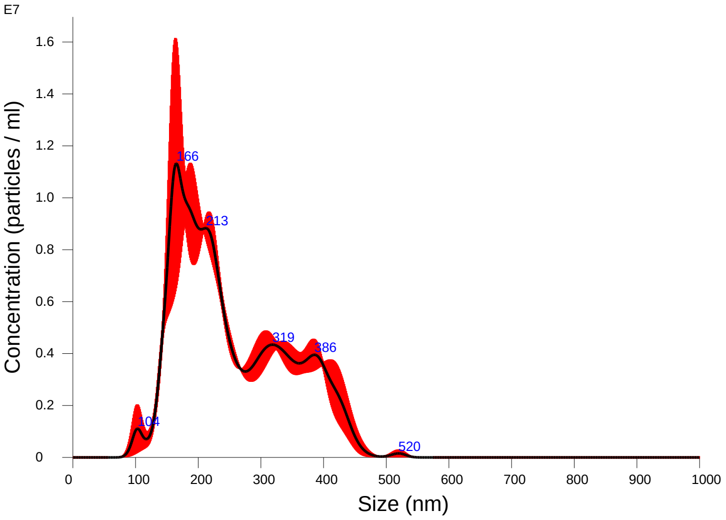

Averaged FTLA Concentration / Size for Experiment:
Plasma_U 2019-11-20 12-51-11
Error bars indicate + / -1 standard error of the mean

Included Files

Plasma_U 2019-11-20 12-51-22
Plasma_U 2019-11-20 12-52-59
Plasma_U 2019-11-20 12-54-12

Details

NTA Version:	NTA 3.2 Dev Build 3.2.16
Script Used:	SOP Standard Measurement 12-51-11PM 20~
Time Captured:	12:51:11 20/11/2019
Operator:	Antonio
Pre-treatment:	
Sample Name:	Plasma_U
Diluent:	
Remarks:	

Capture Settings

Camera Type:	sCMOS
Laser Type:	Green
Camera Level:	9 - 10
Slider Shutter:	607 - 696
Slider Gain:	15 - 73
FPS	25.0
Number of Frames:	1498
Temperature:	23.0 ^{o}C
Viscosity:	(Water) 0.9 cP
Dilution factor:	Dilution not recorded

Analysis Settings

Detect Threshold:	9
Blur Size:	Auto
Max Jump Distance:	Auto: 9.9 - 11.2 pix

Results

Stats: Merged Data

Mean:	252.9 nm
Mode:	165.1 nm
SD:	88.8 nm
D10:	155.2 nm
D50:	226.0 nm
D90:	388.1 nm

Stats: Mean +/- Standard Error

Mean:	253.4 +/- 7.3 nm
Mode:	186.9 +/- 13.1 nm
SD:	88.3 +/- 3.3 nm
D10:	154.4 +/- 2.2 nm
D50:	227.4 +/- 6.3 nm
D90:	389.9 +/- 10.1 nm
Concentration:	1.69e+009 +/- 5.66e+007 particles/ml
	85.6 +/- 2.9 particles/frame
	109.7 +/- 2.8 centres/frame

Plasma_U 2019-11-20 12-51-11

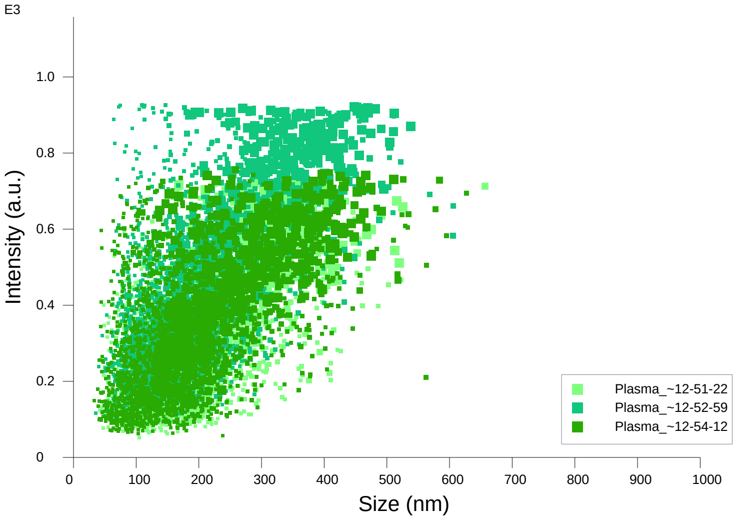

Intensity / Size graph for Experiment:
Plasma_U 2019-11-20 12-51-11

Script Used: (Full Text):

SOP Standard Measurement 12-51-11PM 20Nov2019.txt
