# Supplementary material for: Salting-Out Approach Is Worthy of Comparison with Ultracentrifugation for Extracellular Vesicle Isolation from Tumor and Healthy Models
Source: Biomolecules. 2021 Dec 10;11(12):1857. doi: 10.3390/biom11121857 (PMC8699204; doi:10.3390/biom11121857)
Supplement: Supplementary file 1 [file biomolecules-11-01857-s001.zip › Figure S17.pdf]

# NANOSIGHT

# EV_saliva_S-O_pz2 2021-09-02 16-37-47

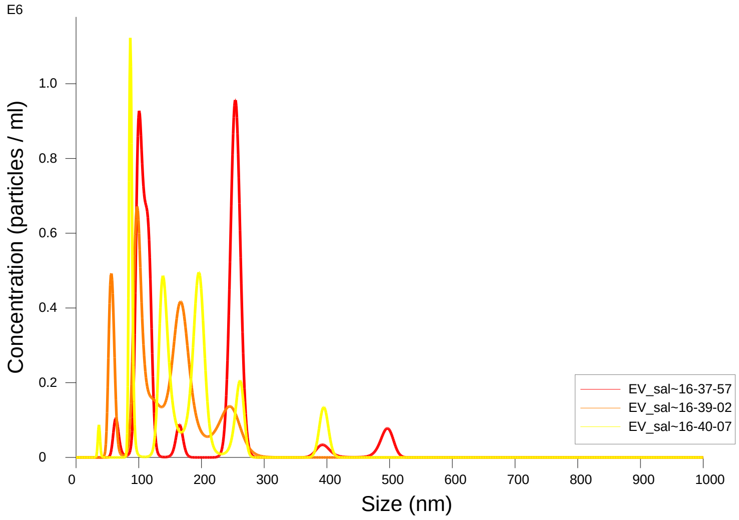


FTLA Concentration / Size graph for Experiment:
EV_saliva_S-O_pz2 2021-09-02 16-37-47

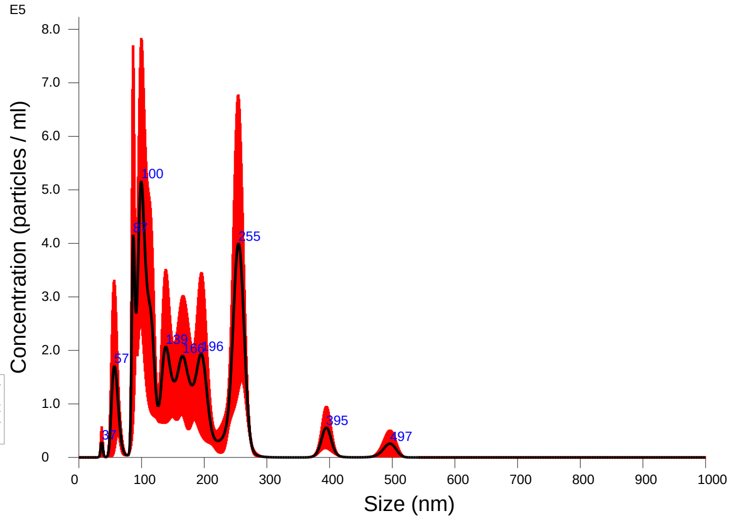


Averaged FTLA Concentration / Size for Experiment:
EV_saliva_S-O_pz2 2021-09-02 16-37-47
Error bars indicate + / -1 standard error of the mean

## Included Files

EV_saliva_S-O_pz2 2021-09-02 16-37-57
EV_saliva_S-O_pz2 2021-09-02 16-39-02
EV_saliva_S-O_pz2 2021-09-02 16-40-07

## Details

| | |
|---|---|
| NTA Version: | NTA 3.2 Dev Build 3.2.16 |
| Script Used: | SOP Standard Measurement 04-37-47PM 02~ |
| Time Captured: | 16:37:47 02/09/2021 |
| Operator: | simo |
| Pre-treatment: | |
| Sample Name: | EV_saliva_S-O_pz2 |
| Diluent: | |
| Remarks: | 1:1000 |

## Capture Settings

| | |
|---|---|
| Camera Type: | sCMOS |
| Laser Type: | Green |
| Camera Level: | 14 |
| Slider Shutter: | 1259 |
| Slider Gain: | 366 |
| FPS | 25.0 |
| Number of Frames: | 1498 |
| Temperature: | 26.5 $^{o}$C |
| Viscosity: | (Water) 0.858 - 0.859 cP |
| Dilution factor: | Dilution not recorded |
| Syringe Pump Speed: | 50 |

## Analysis Settings

| | |
|---|---|
| Detect Threshold: | 5 |
| Blur Size: | Auto |
| Max Jump Distance: | Auto: 14.0 - 32.3 pix |

## Results

Stats: Merged Data

| | |
|---|---|
| Mean: | 170.9 nm |
| Mode: | 99.5 nm |
| SD: | 85.2 nm |
| D10: | 87.0 nm |
| D50: | 152.5 nm |
| D90: | 259.5 nm |

Stats: Mean +/- Standard Error

| | |
|---|---|
| Mean: | 170.8 +/- 14.8 nm |
| Mode: | 146.0 +/- 54.1 nm |
| SD: | 79.9 +/- 12.7 nm |
| D10: | 80.5 +/- 11.2 nm |
| D50: | 158.6 +/- 9.1 nm |
| D90: | 253.7 +/- 9.9 nm |
| Concentration: | 4.10e+007 +/- 2.75e+006 particles/ml |
| | 2.1 +/- 0.1 particles/frame |
| | 2.5 +/- 0.2 centres/frame |

NANOSIGHT

# EV_saliva_S-O_pz2 2021-09-02 16-37-47

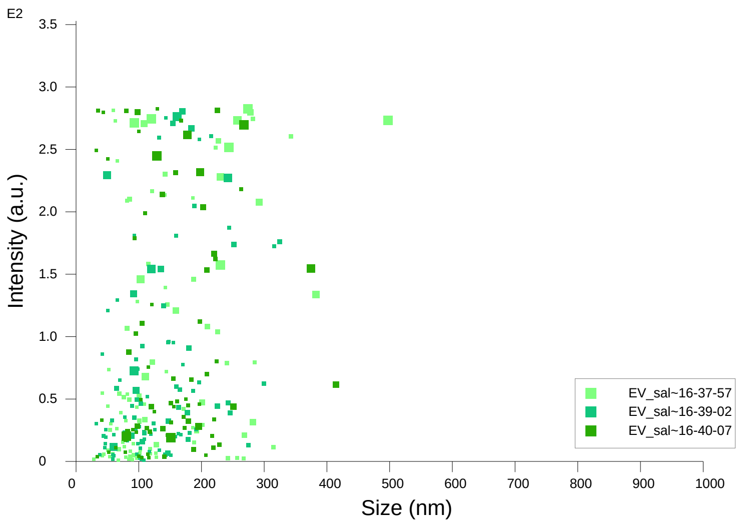


Intensity / Size graph for Experiment:
EV_saliva_S-O_pz2 2021-09-02 16-37-47

**Script Used: (Full Text):**

SOP Standard Measurement 04-37-47PM 02Sep2021.txt
