# Supplementary material for: Salting-Out Approach Is Worthy of Comparison with Ultracentrifugation for Extracellular Vesicle Isolation from Tumor and Healthy Models
Source: Biomolecules. 2021 Dec 10;11(12):1857. doi: 10.3390/biom11121857 (PMC8699204; doi:10.3390/biom11121857)
Supplement: Supplementary file 1 [file biomolecules-11-01857-s001.zip › Figure S18.pdf]

NANOSIGHT

EV_saliva_UC_pz2 OK 2021-09-02 16-45-16

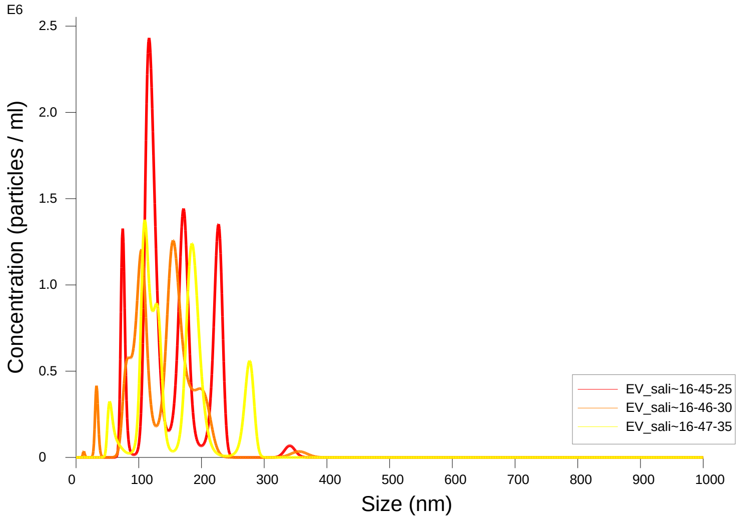

FTLA Concentration / Size graph for Experiment:
EV_saliva_UC_pz2 OK 2021-09-02 16-45-16

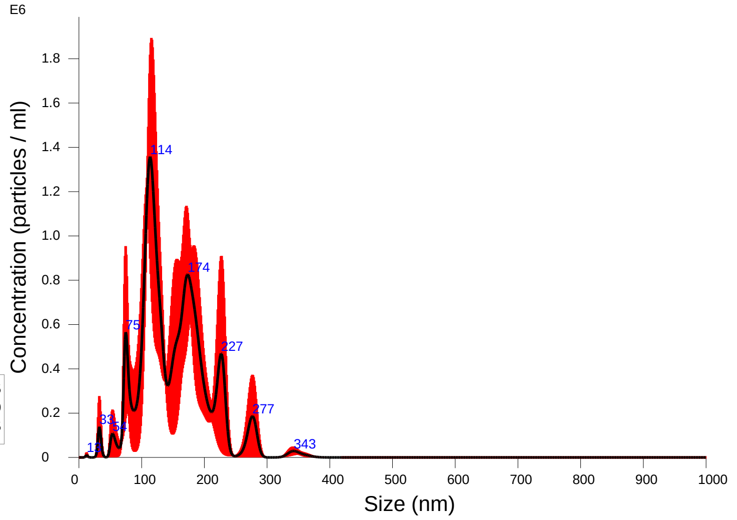

Averaged FTLA Concentration / Size for Experiment:
EV_saliva_UC_pz2 OK 2021-09-02 16-45-16
Error bars indicate + / -1 standard error of the mean

Included Files

EV_saliva_UC_pz2 OK 2021-09-02 16-45-25
EV_saliva_UC_pz2 OK 2021-09-02 16-46-30
EV_saliva_UC_pz2 OK 2021-09-02 16-47-35

Details

NTA Version:	NTA 3.2 Dev Build 3.2.16
Script Used:	SOP Standard Measurement 04-45-16PM 02~
Time Captured:	16:45:16 02/09/2021
Operator:	simo
Pre-treatment:	
Sample Name:	EV_saliva_UC_pz2 OK
Diluent:	
Remarks:	1:1000

Capture Settings

Camera Type:	sCMOS
Laser Type:	Green
Camera Level:	14
Slider Shutter:	1259
Slider Gain:	366
FPS	25.0
Number of Frames:	1498
Temperature:	26.5 ^{o}C
Viscosity:	(Water) 0.858 - 0.859 cP
Dilution factor:	Dilution not recorded
Syringe Pump Speed:	50

Analysis Settings

Detect Threshold:	5
Blur Size:	Auto
Max Jump Distance:	Auto: 14.9 - 22.8 pix

Results

Stats: Merged Data

Mean:	150.9 nm
Mode:	113.5 nm
SD:	53.6 nm
D10:	90.8 nm
D50:	142.8 nm
D90:	222.2 nm

Stats: Mean +/- Standard Error

Mean:	150.9 +/- 6.2 nm
Mode:	127.0 +/- 14.1 nm
SD:	53.0 +/- 2.7 nm
D10:	95.9 +/- 6.1 nm
D50:	142.8 +/- 4.2 nm
D90:	229.6 +/- 21.0 nm
Concentration:	9.80e+007 +/- 9.14e+006 particles/ml
	5.0 +/- 0.5 particles/frame
	6.4 +/- 0.5 centres/frame

NANOSIGHT

EV_saliva_UC_pz2 OK 2021-09-02 16-45-16

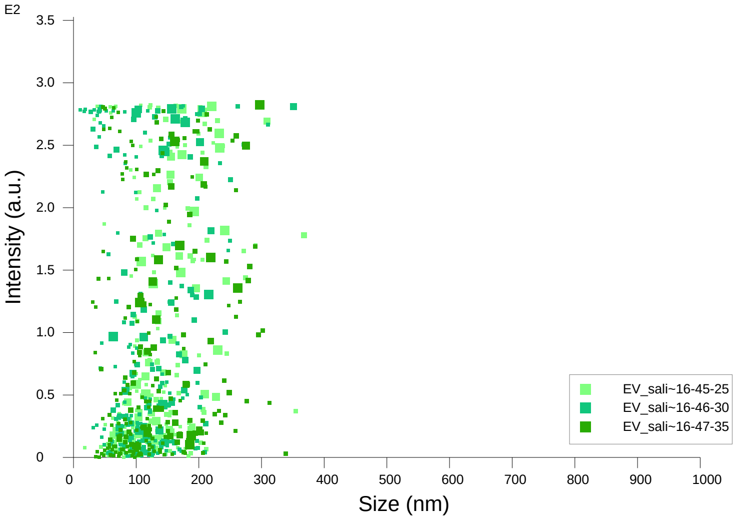

Intensity / Size graph for Experiment:
EV_saliva_UC_pz2 OK 2021-09-02 16-45-16

Script Used: (Full Text):

SOP Standard Measurement 04-45-16PM 02Sep2021.txt
