# Supplementary material for: Salting-Out Approach Is Worthy of Comparison with Ultracentrifugation for Extracellular Vesicle Isolation from Tumor and Healthy Models
Source: Biomolecules. 2021 Dec 10;11(12):1857. doi: 10.3390/biom11121857 (PMC8699204; doi:10.3390/biom11121857)
Supplement: Supplementary file 1 [file biomolecules-11-01857-s001.zip › Figure S19.pdf]

NANOSIGHT

EV_SALIVA_S-O_PZ3 2021-09-03 12-12-00

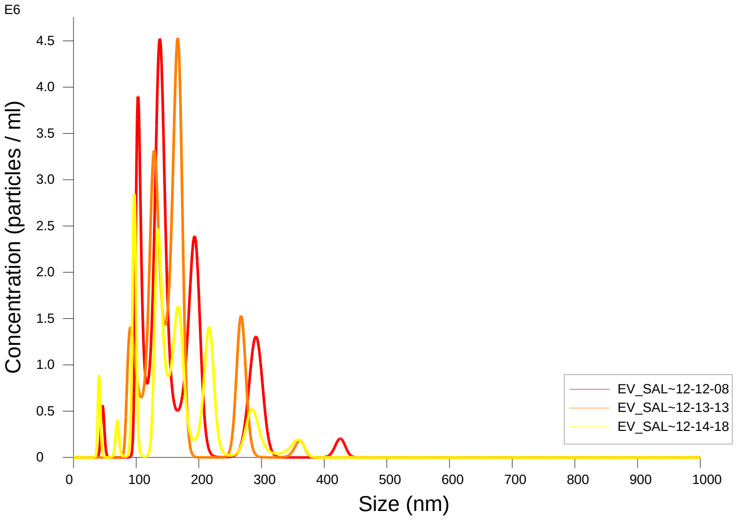

FTLA Concentration / Size graph for Experiment:
EV_SALIVA_S-O_PZ3 2021-09-03 12-12-00

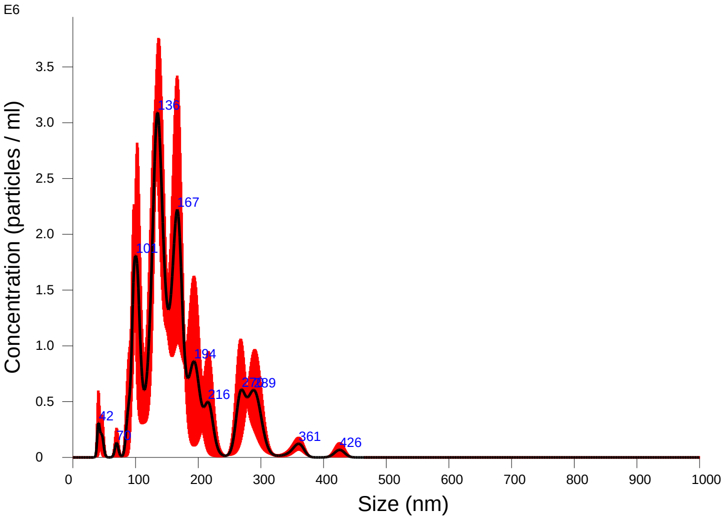

Averaged FTLA Concentration / Size for Experiment:
EV_SALIVA_S-O_PZ3 2021-09-03 12-12-00
Error bars indicate + / -1 standard error of the mean

Included Files

EV_SALIVA_S-O_PZ3 2021-09-03 12-12-08
EV_SALIVA_S-O_PZ3 2021-09-03 12-13-13
EV_SALIVA_S-O_PZ3 2021-09-03 12-14-18

Details

NTA Version:	NTA 3.2 Dev Build 3.2.16
Script Used:	SOP Standard Measurement 12-12-00PM 03~
Time Captured:	12:12:00 03/09/2021
Operator:	simo
Pre-treatment:	
Sample Name:	EV_SALIVA_S-O_PZ3
Diluent:	
Remarks:	1:1000

Capture Settings

Camera Type:	sCMOS
Laser Type:	Green
Camera Level:	14
Slider Shutter:	1259
Slider Gain:	366
FPS	25.0
Number of Frames:	1498
Temperature:	25.0 ^{o}C
Viscosity:	(Water) 0.887 - 0.888 cP
Dilution factor:	Dilution not recorded
Syringe Pump Speed:	50

Analysis Settings

Detect Threshold:	5
Blur Size:	Auto
Max Jump Distance:	Auto: 10.8 - 17.7 pix

Results

Stats: Merged Data

Mean:	167.2 nm
Mode:	135.1 nm
SD:	63.8 nm
D10:	100.5 nm
D50:	150.6 nm
D90:	273.7 nm

Stats: Mean +/- Standard Error

Mean:	167.6 +/- 3.3 nm
Mode:	133.7 +/- 20.1 nm
SD:	63.6 +/- 4.6 nm
D10:	100.4 +/- 2.6 nm
D50:	152.3 +/- 4.6 nm
D90:	276.8 +/- 7.0 nm
Concentration:	2.13e+008 +/- 2.78e+007 particles/ml
	10.8 +/- 1.4 particles/frame
	12.9 +/- 1.0 centres/frame

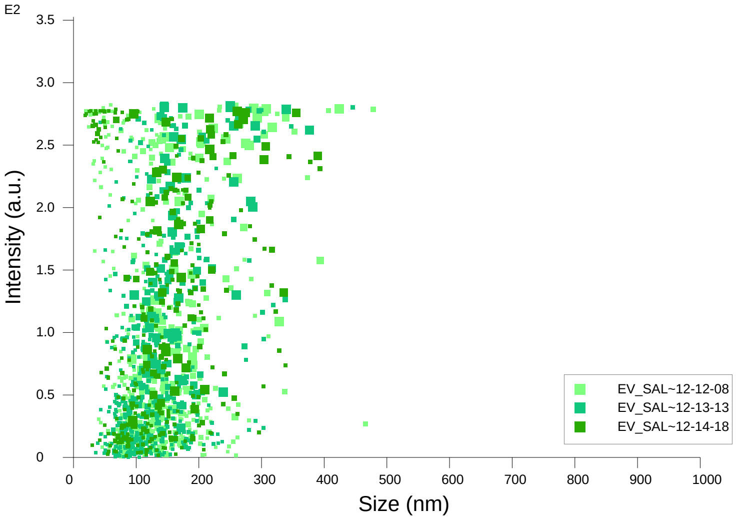

Intensity / Size graph for Experiment:
EV_SALIVA_S-O_PZ3 2021-09-03 12-12-00

Script Used: (Full Text):

SOP Standard Measurement 12-12-00PM 03Sep2021.txt
