# Supplementary material for: Salting-Out Approach Is Worthy of Comparison with Ultracentrifugation for Extracellular Vesicle Isolation from Tumor and Healthy Models
Source: Biomolecules. 2021 Dec 10;11(12):1857. doi: 10.3390/biom11121857 (PMC8699204; doi:10.3390/biom11121857)
Supplement: Supplementary file 1 [file biomolecules-11-01857-s001.zip › Figure S2.pdf]

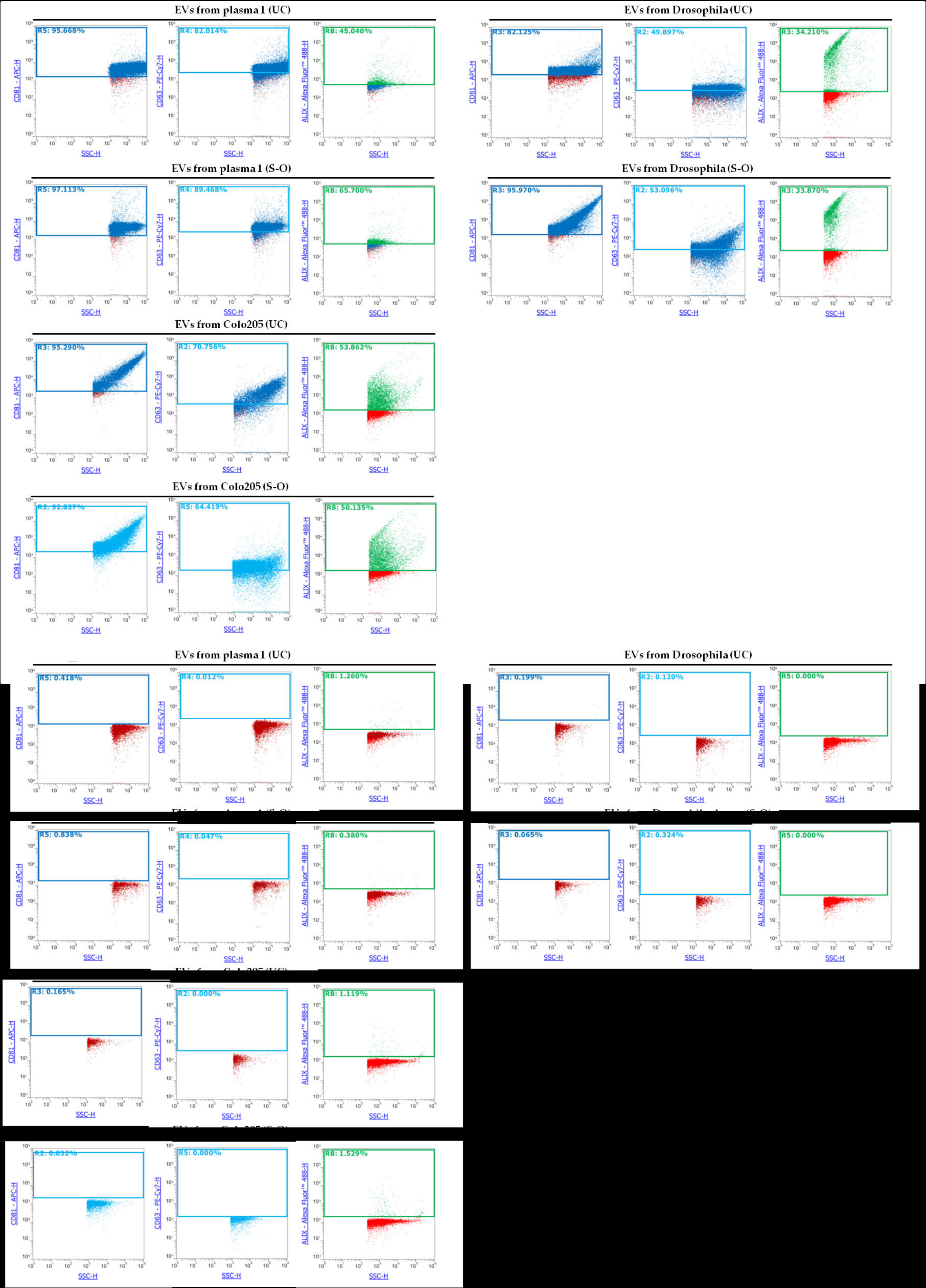

EVs from plasma 1 (UC)
R5: 95.668%
R4: 82.014%
R8: 45.040%
EVs from Drosophila (UC)
R3: 82.125%
R2: 49.897%
R3: 34.210%
EVs from plasma 1 (S-O)
R5: 97.113%
R4: 89.468%
R8: 65.700%
EVs from Drosophila (S-O)
R3: 95.970%
R2: 53.096%
R3: 33.870%
EVs from Colo205 (UC)
R3: 95.290%
R2: 70.756%
R8: 53.862%
EVs from Colo205 (S-O)
R2: 92.827%
R5: 64.419%
R8: 50.135%
EVs from plasma 1 (UC)
R5: 0.418%
R4: 0.012%
R8: 1.260%
EVs from Drosophila (UC)
R3: 0.199%
R2: 0.120%
R5: 0.000%
R5: 0.838%
R4: 0.047%
R8: 0.380%
R3: 0.065%
R2: 0.324%
R5: 0.000%
R3: 0.165%
R2: 0.000%
R8: 1.119%
R2: 0.032%
R5: 0.000%
R8: 1.529%
CD81 - APC-H
CD63 - PE-Cy7-H
ALIX - Alexa Fluor™ 488-H
SSC-H
