# Supplementary material for: Salting-Out Approach Is Worthy of Comparison with Ultracentrifugation for Extracellular Vesicle Isolation from Tumor and Healthy Models
Source: Biomolecules. 2021 Dec 10;11(12):1857. doi: 10.3390/biom11121857 (PMC8699204; doi:10.3390/biom11121857)
Supplement: Supplementary file 1 [file biomolecules-11-01857-s001.zip › Figure S20.pdf]

NANOSIGHT

EV_SALIVA_ULTRA_PZ3 2021-09-03 12-20-45

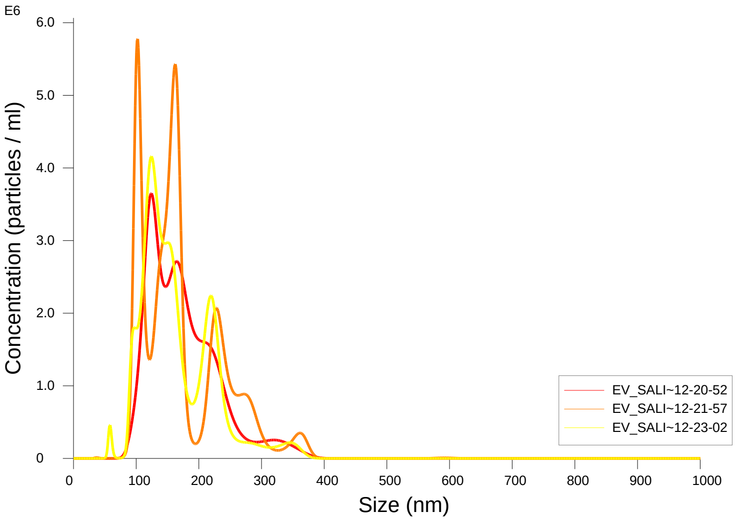

FTLA Concentration / Size graph for Experiment:
EV_SALIVA_ULTRA_PZ3 2021-09-03 12-20-45

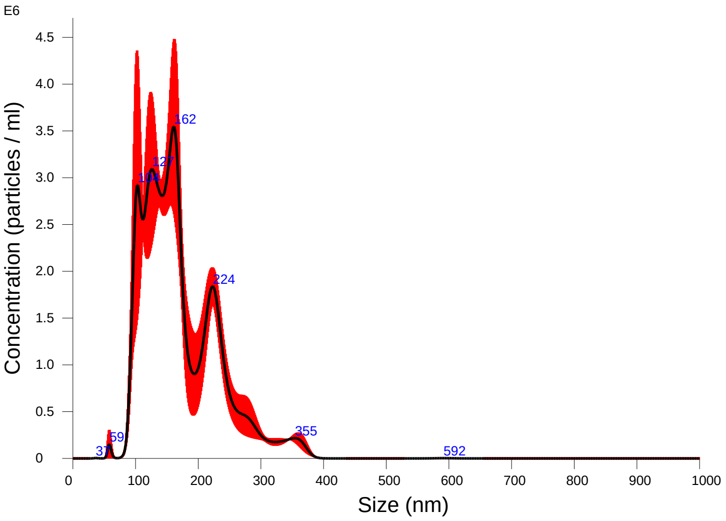

Averaged FTLA Concentration / Size for Experiment:
EV_SALIVA_ULTRA_PZ3 2021-09-03 12-20-45
Error bars indicate + / -1 standard error of the mean

Included Files

EV_SALIVA_ULTRA_PZ3 2021-09-03 12-20-52
EV_SALIVA_ULTRA_PZ3 2021-09-03 12-21-57
EV_SALIVA_ULTRA_PZ3 2021-09-03 12-23-02

Details

NTA Version:	NTA 3.2 Dev Build 3.2.16
Script Used:	SOP Standard Measurement 12-20-45PM 03~
Time Captured:	12:20:45 03/09/2021
Operator:	simo
Pre-treatment:	
Sample Name:	EV_SALIVA_ULTRA_PZ3
Diluent:	
Remarks:	1:1000

Capture Settings

Camera Type:	sCMOS
Laser Type:	Green
Camera Level:	14
Slider Shutter:	1259
Slider Gain:	366
FPS	25.0
Number of Frames:	1498
Temperature:	25.2 ^{o}C
Viscosity:	(Water) 0.883 - 0.885 cP
Dilution factor:	Dilution not recorded
Syringe Pump Speed:	50

Analysis Settings

Detect Threshold:	5
Blur Size:	Auto
Max Jump Distance:	Auto: 11.1 - 13.2 pix

Results

Stats: Merged Data

Mean:	170.6 nm
Mode:	161.0 nm
SD:	60.1 nm
D10:	104.6 nm
D50:	156.1 nm
D90:	248.8 nm

Stats: Mean +/- Standard Error

Mean:	170.5 +/- 3.0 nm
Mode:	116.9 +/- 7.4 nm
SD:	59.5 +/- 3.0 nm
D10:	106.7 +/- 3.9 nm
D50:	156.0 +/- 4.2 nm
D90:	247.5 +/- 9.4 nm
Concentration:	3.74e+008 +/- 2.11e+007 particles/ml
	19.0 +/- 1.1 particles/frame
	22.5 +/- 1.2 centres/frame

NANOSIGHT

EV_SALIVA_ULTRA_PZ3 2021-09-03 12-20-45

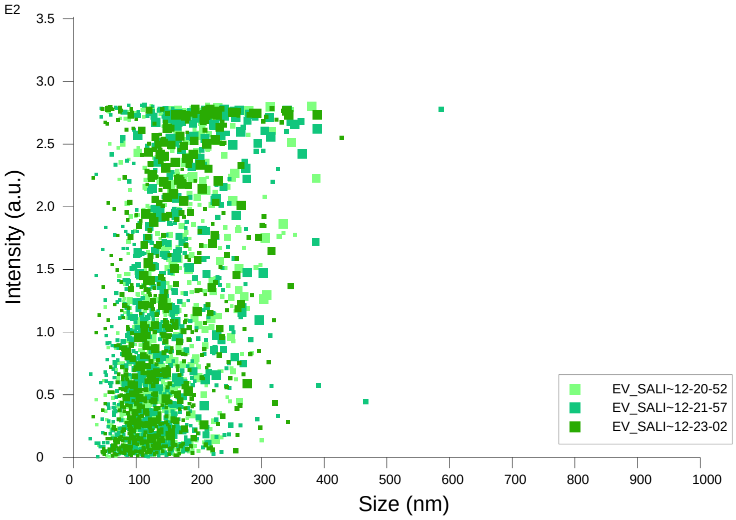

Intensity / Size graph for Experiment:
EV_SALIVA_ULTRA_PZ3 2021-09-03 12-20-45

Script Used: (Full Text):

SOP Standard Measurement 12-20-45PM 03Sep2021.txt
