# Supplementary material for: Salting-Out Approach Is Worthy of Comparison with Ultracentrifugation for Extracellular Vesicle Isolation from Tumor and Healthy Models
Source: Biomolecules. 2021 Dec 10;11(12):1857. doi: 10.3390/biom11121857 (PMC8699204; doi:10.3390/biom11121857)
Supplement: Supplementary file 1 [file biomolecules-11-01857-s001.zip › Figure S21.pdf]

NANOSIGHT

Saliva_NaAc 2019-11-20 11-39-26

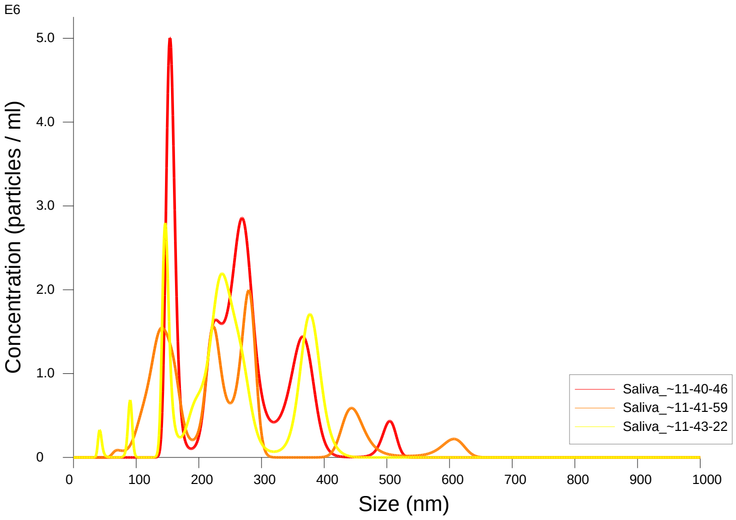

FTLA Concentration / Size graph for Experiment:
Saliva_NaAc 2019-11-20 11-39-26

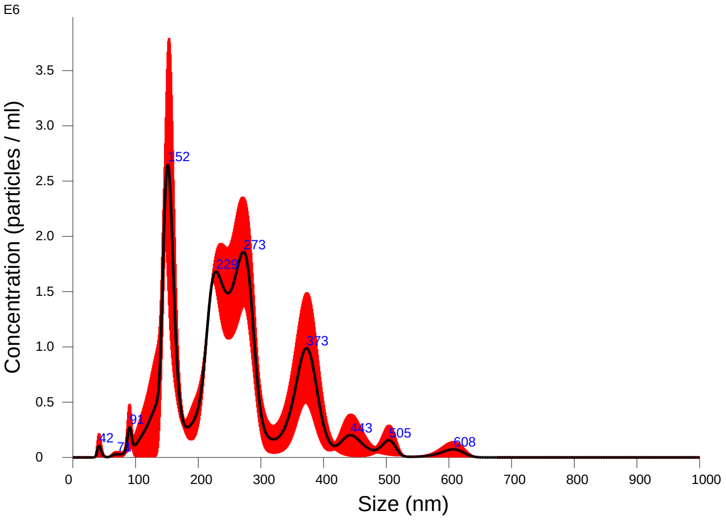

Averaged FTLA Concentration / Size for Experiment:
Saliva_NaAc 2019-11-20 11-39-26
Error bars indicate + / -1 standard error of the mean

Included Files

Saliva_NaAc 2019-11-20 11-40-46
Saliva_NaAc 2019-11-20 11-41-59
Saliva_NaAc 2019-11-20 11-43-22

Details

NTA Version:	NTA 3.2 Dev Build 3.2.16
Script Used:	SOP Standard Measurement 11-39-26AM 20~
Time Captured:	11:39:26 20/11/2019
Operator:	Antonio
Pre-treatment:	
Sample Name:	Saliva_NaAc
Diluent:	
Remarks:	

Capture Settings

Camera Type:	sCMOS
Laser Type:	Green
Camera Level:	11
Slider Shutter:	890
Slider Gain:	146
FPS	25.0
Number of Frames:	1498
Temperature:	23.0 ^{o}C
Viscosity:	(Water) 0.9 cP
Dilution factor:	Dilution not recorded

Analysis Settings

Detect Threshold:	5
Blur Size:	Auto
Max Jump Distance:	Auto: 8.5 - 14.0 pix

Results

Stats: Merged Data

Mean:	256.9 nm
Mode:	151.5 nm
SD:	97.6 nm
D10:	145.6 nm
D50:	246.9 nm
D90:	382.4 nm

Stats: Mean +/- Standard Error

Mean:	256.1 +/- 3.9 nm
Mode:	193.4 +/- 43.2 nm
SD:	98.1 +/- 12.3 nm
D10:	141.5 +/- 8.0 nm
D50:	243.2 +/- 9.0 nm
D90:	399.9 +/- 23.7 nm
Concentration:	2.88e+008 +/- 3.48e+007 particles/ml
	14.6 +/- 1.8 particles/frame
	22.8 +/- 2.9 centres/frame

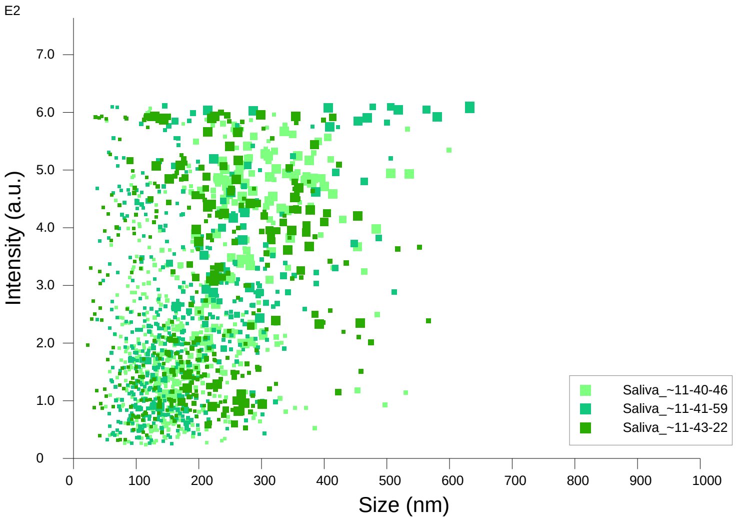

Intensity / Size graph for Experiment:
Saliva_NaAc 2019-11-20 11-39-26

Script Used: (Full Text):

SOP Standard Measurement 11-39-26AM 20Nov2019.txt
