# Supplementary material for: Salting-Out Approach Is Worthy of Comparison with Ultracentrifugation for Extracellular Vesicle Isolation from Tumor and Healthy Models
Source: Biomolecules. 2021 Dec 10;11(12):1857. doi: 10.3390/biom11121857 (PMC8699204; doi:10.3390/biom11121857)
Supplement: Supplementary file 1 [file biomolecules-11-01857-s001.zip › Figure S22.pdf]

NANOSIGHT

Saliva_U 2019-11-20 13-16-39

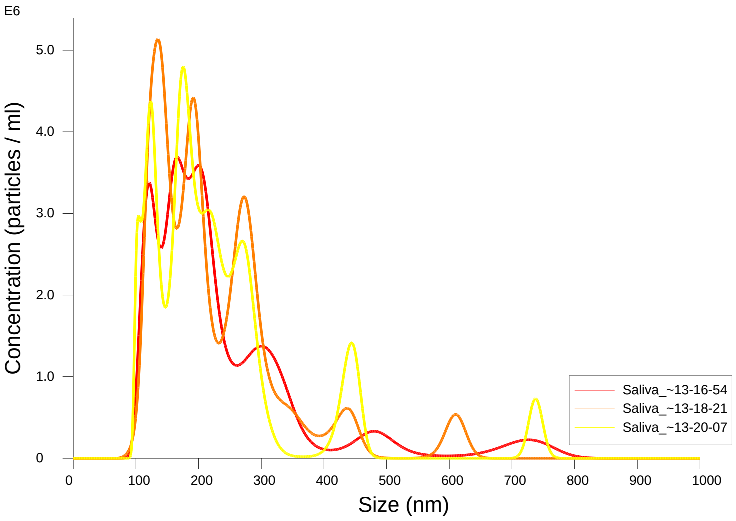

FTLA Concentration / Size graph for Experiment:
Saliva_U 2019-11-20 13-16-39

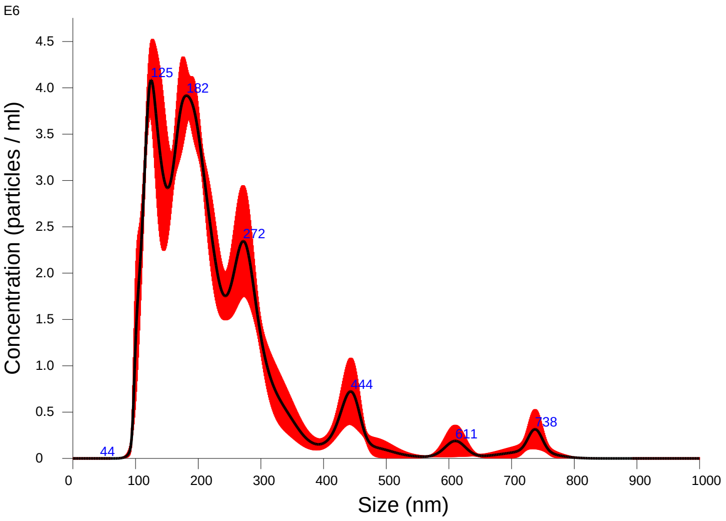

Averaged FTLA Concentration / Size for Experiment:
Saliva_U 2019-11-20 13-16-39
Error bars indicate + / -1 standard error of the mean

Included Files

Saliva_U 2019-11-20 13-16-54
Saliva_U 2019-11-20 13-18-21
Saliva_U 2019-11-20 13-20-07

Details

NTA Version:	NTA 3.2 Dev Build 3.2.16
Script Used:	SOP Standard Measurement 01-16-39PM 20~
Time Captured:	13:16:39 20/11/2019
Operator:	Antonio
Pre-treatment:	
Sample Name:	Saliva_U
Diluent:	
Remarks:	

Capture Settings

Camera Type:	sCMOS
Laser Type:	Green
Camera Level:	12
Slider Shutter:	1200
Slider Gain:	146
FPS	25.0
Number of Frames:	1498
Temperature:	23.0 ^{o}C
Viscosity:	(Water) 0.9 cP
Dilution factor:	Dilution not recorded

Analysis Settings

Detect Threshold:	5
Blur Size:	Auto
Max Jump Distance:	Auto: 9.5 - 10.9 pix

Results

Stats: Merged Data

Mean:	231.0 nm
Mode:	124.9 nm
SD:	120.9 nm
D10:	121.9 nm
D50:	197.5 nm
D90:	363.9 nm

Stats: Mean +/- Standard Error

Mean:	231.2 +/- 3.1 nm
Mode:	158.7 +/- 12.2 nm
SD:	120.9 +/- 7.7 nm
D10:	121.9 +/- 2.0 nm
D50:	197.6 +/- 0.4 nm
D90:	378.2 +/- 21.5 nm
Concentration:	6.69e+008 +/- 2.81e+007 particles/ml
	34.0 +/- 1.4 particles/frame
	48.2 +/- 4.8 centres/frame

NANOSIGHT

Saliva_U 2019-11-20 13-16-39

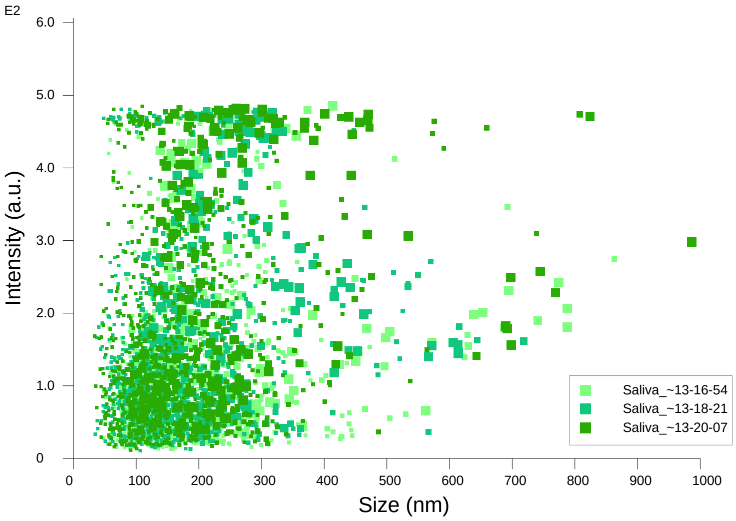

Intensity / Size graph for Experiment:
Saliva_U 2019-11-20 13-16-39

Script Used: (Full Text):

SOP Standard Measurement 01-16-39PM 20Nov2019.txt
