# Supplementary material for: Salting-Out Approach Is Worthy of Comparison with Ultracentrifugation for Extracellular Vesicle Isolation from Tumor and Healthy Models
Source: Biomolecules. 2021 Dec 10;11(12):1857. doi: 10.3390/biom11121857 (PMC8699204; doi:10.3390/biom11121857)
Supplement: Supplementary file 1 [file biomolecules-11-01857-s001.zip › Figure S23.pdf]

NANOSIGHT

EV_URINE_S-O_PZ2 2021-09-03 11-04-53

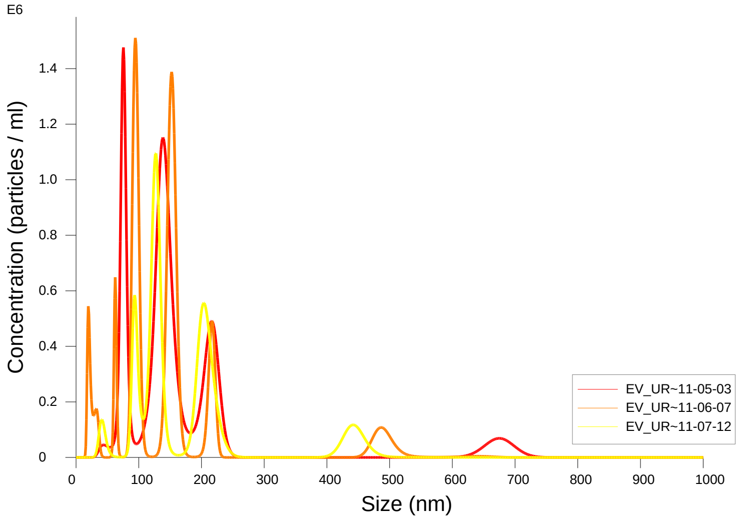

FTLA Concentration / Size graph for Experiment:
EV_URINE_S-O_PZ2 2021-09-03 11-04-53

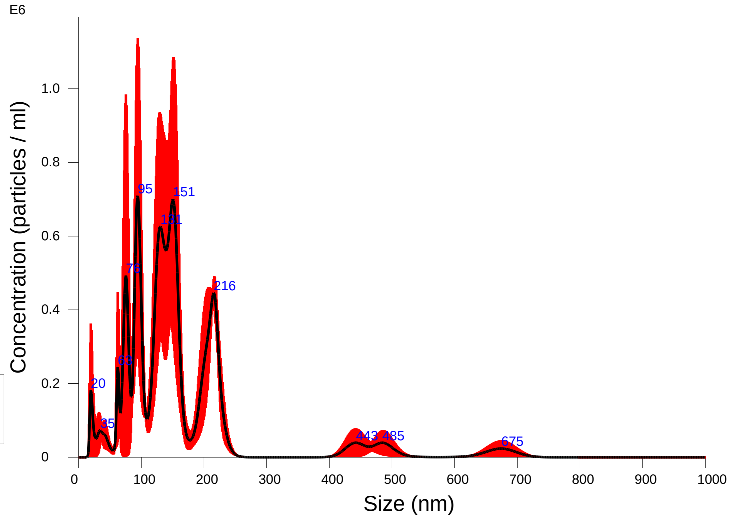

Averaged FTLA Concentration / Size for Experiment:
EV_URINE_S-O_PZ2 2021-09-03 11-04-53
Error bars indicate + / -1 standard error of the mean

Included Files

EV_URINE_S-O_PZ2 2021-09-03 11-05-03
EV_URINE_S-O_PZ2 2021-09-03 11-06-07
EV_URINE_S-O_PZ2 2021-09-03 11-07-12

Details

NTA Version:	NTA 3.2 Dev Build 3.2.16
Script Used:	SOP Standard Measurement 11-04-53AM 03~
Time Captured:	11:04:53 03/09/2021
Operator:	simo
Pre-treatment:	
Sample Name:	EV_URINE_S-O_PZ2
Diluent:	
Remarks:	1:1000

Capture Settings

Camera Type:	sCMOS
Laser Type:	Green
Camera Level:	15
Slider Shutter:	1206
Slider Gain:	366
FPS	25.0
Number of Frames:	1498
Temperature:	23.2 - 23.3 ^{o}C
Viscosity:	(Water) 0.924 - 0.926 cP
Dilution factor:	Dilution not recorded
Syringe Pump Speed:	50

Analysis Settings

Detect Threshold:	5
Blur Size:	Auto
Max Jump Distance:	Auto: 14.0 - 26.9 pix

Results

Stats: Merged Data

Mean:	165.1 nm
Mode:	94.2 nm
SD:	116.1 nm
D10:	74.0 nm
D50:	139.4 nm
D90:	224.2 nm

Stats: Mean +/- Standard Error

Mean:	165.6 +/- 7.5 nm
Mode:	99.0 +/- 15.1 nm
SD:	113.1 +/- 8.8 nm
D10:	75.6 +/- 9.4 nm
D50:	139.0 +/- 3.1 nm
D90:	229.0 +/- 7.9 nm
Concentration:	6.49e+007 +/- 6.82e+006 particles/ml
	3.3 +/- 0.3 particles/frame
	6.6 +/- 1.4 centres/frame

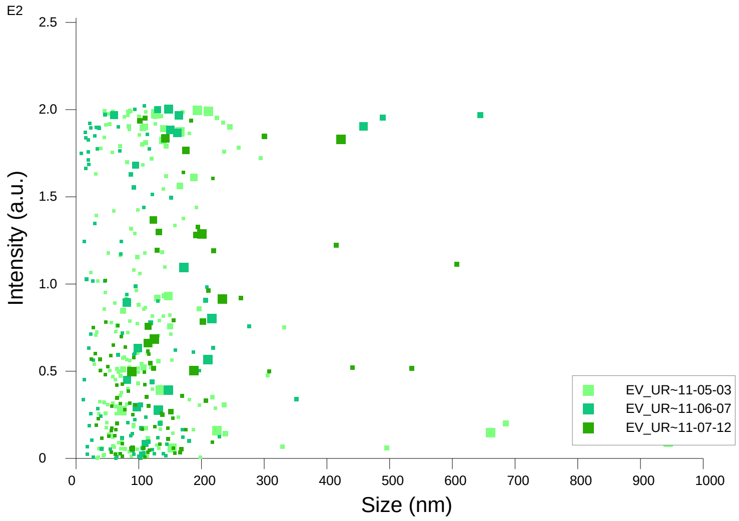

Intensity / Size graph for Experiment:
EV_URINE_S-O_PZ2 2021-09-03 11-04-53

Script Used: (Full Text):

SOP Standard Measurement 11-04-53AM 03Sep2021.txt
