# Supplementary material for: Salting-Out Approach Is Worthy of Comparison with Ultracentrifugation for Extracellular Vesicle Isolation from Tumor and Healthy Models
Source: Biomolecules. 2021 Dec 10;11(12):1857. doi: 10.3390/biom11121857 (PMC8699204; doi:10.3390/biom11121857)
Supplement: Supplementary file 1 [file biomolecules-11-01857-s001.zip › Figure S24.pdf]

NANOSIGHT

EV_URINE_ULTRA_PZ2 2021-09-03 10-55-05

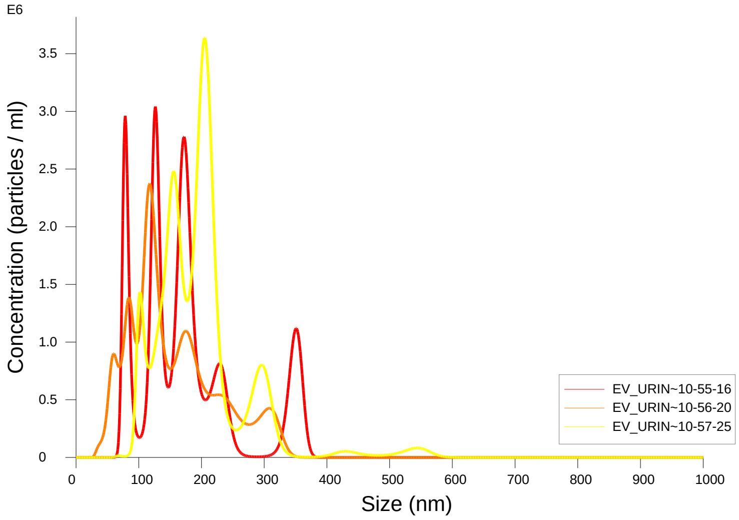

FTLA Concentration / Size graph for Experiment:
EV_URINE_ULTRA_PZ2 2021-09-03 10-55-05

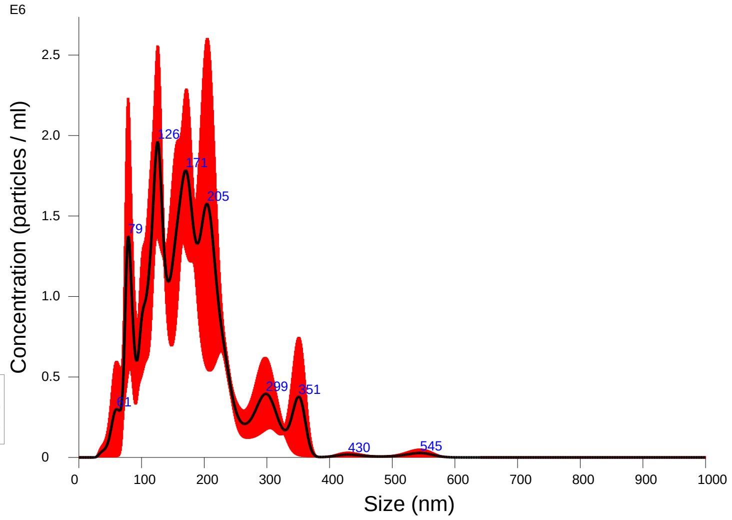

Averaged FTLA Concentration / Size for Experiment:
EV_URINE_ULTRA_PZ2 2021-09-03 10-55-05
Error bars indicate + / -1 standard error of the mean

Included Files

EV_URINE_ULTRA_PZ2 2021-09-03 10-55-16
EV_URINE_ULTRA_PZ2 2021-09-03 10-56-20
EV_URINE_ULTRA_PZ2 2021-09-03 10-57-25

Details

NTA Version:	NTA 3.2 Dev Build 3.2.16
Script Used:	SOP Standard Measurement 10-55-05AM 03~
Time Captured:	10:55:05 03/09/2021
Operator:	simo
Pre-treatment:	
Sample Name:	EV_URINE_ULTRA_PZ2
Diluent:	
Remarks:	1:1000

Capture Settings

Camera Type:	sCMOS
Laser Type:	Green
Camera Level:	13
Slider Shutter:	1232
Slider Gain:	219
FPS	25.0
Number of Frames:	1498
Temperature:	23.0 ^{o}C
Viscosity:	(Water) 0.930 - 0.931 cP
Dilution factor:	Dilution not recorded
Syringe Pump Speed:	50

Analysis Settings

Detect Threshold:	5
Blur Size:	Auto
Max Jump Distance:	Auto: 10.9 - 14.6 pix

Results

Stats: Merged Data

Mean:	177.9 nm
Mode:	125.7 nm
SD:	76.2 nm
D10:	88.1 nm
D50:	168.0 nm
D90:	286.9 nm

Stats: Mean +/- Standard Error

Mean:	176.0 +/- 12.2 nm
Mode:	149.7 +/- 27.8 nm
SD:	74.2 +/- 2.6 nm
D10:	91.5 +/- 14.7 nm
D50:	163.4 +/- 16.8 nm
D90:	296.9 +/- 22.1 nm
Concentration:	2.59e+008 +/- 2.20e+007 particles/ml
	13.1 +/- 1.1 particles/frame
	17.1 +/- 1.6 centres/frame

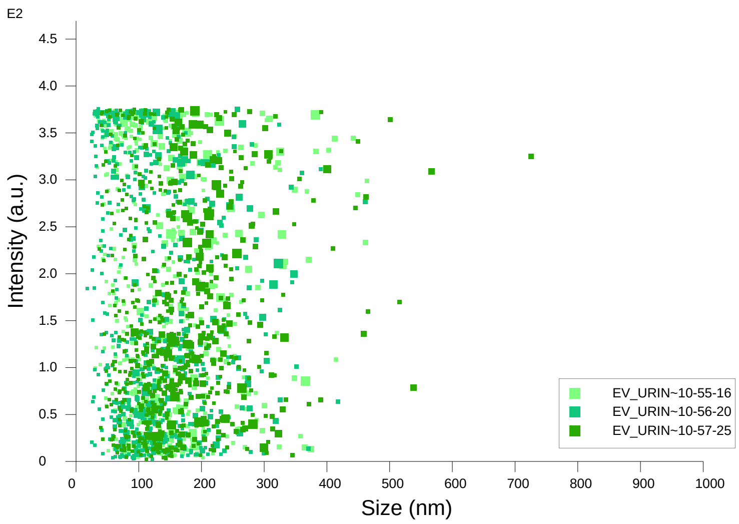

Intensity / Size graph for Experiment:
EV_URINE_ULTRA_PZ2 2021-09-03 10-55-05

Script Used: (Full Text):

SOP Standard Measurement 10-55-05AM 03Sep2021.txt
