# Supplementary material for: Salting-Out Approach Is Worthy of Comparison with Ultracentrifugation for Extracellular Vesicle Isolation from Tumor and Healthy Models
Source: Biomolecules. 2021 Dec 10;11(12):1857. doi: 10.3390/biom11121857 (PMC8699204; doi:10.3390/biom11121857)
Supplement: Supplementary file 1 [file biomolecules-11-01857-s001.zip › Figure S25.pdf]

NANOSIGHT

EV_URINE_S-O_PZ3 2021-09-03 11-34-22

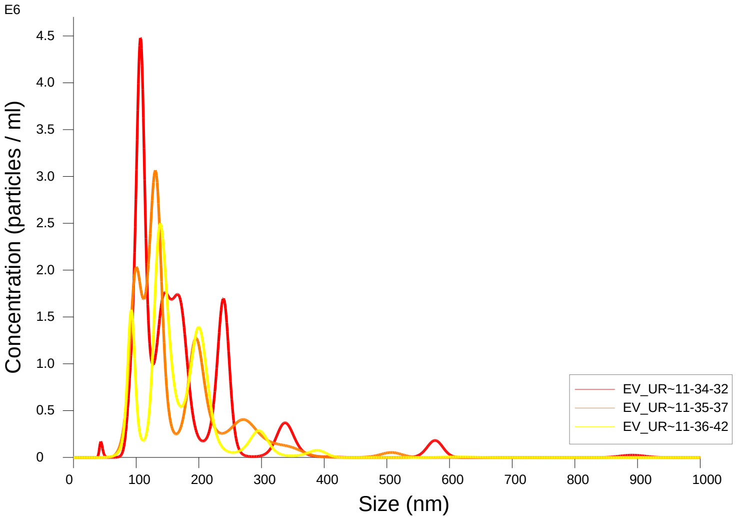

FTLA Concentration / Size graph for Experiment:
EV_URINE_S-O_PZ3 2021-09-03 11-34-22

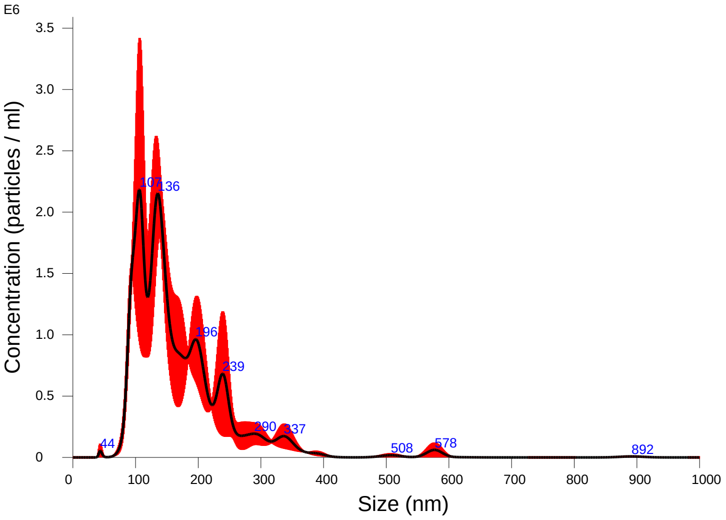

Averaged FTLA Concentration / Size for Experiment:
EV_URINE_S-O_PZ3 2021-09-03 11-34-22
Error bars indicate + / -1 standard error of the mean

Included Files

EV_URINE_S-O_PZ3 2021-09-03 11-34-32
EV_URINE_S-O_PZ3 2021-09-03 11-35-37
EV_URINE_S-O_PZ3 2021-09-03 11-36-42

Details

NTA Version:	NTA 3.2 Dev Build 3.2.16
Script Used:	SOP Standard Measurement 11-34-22AM 03~
Time Captured:	11:34:22 03/09/2021
Operator:	simo
Pre-treatment:	
Sample Name:	EV_URINE_S-O_PZ3
Diluent:	
Remarks:	1:1000

Capture Settings

Camera Type:	sCMOS
Laser Type:	Green
Camera Level:	14
Slider Shutter:	1259
Slider Gain:	366
FPS	25.0
Number of Frames:	1498
Temperature:	23.9 - 24.0 ^{o}C
Viscosity:	(Water) 0.909 - 0.911 cP
Dilution factor:	Dilution not recorded
Syringe Pump Speed:	50

Analysis Settings

Detect Threshold:	5
Blur Size:	Auto
Max Jump Distance:	Auto: 11.7 - 11.9 pix

Results

Stats: Merged Data

Mean:	170.2 nm
Mode:	106.1 nm
SD:	84.1 nm
D10:	97.6 nm
D50:	144.3 nm
D90:	257.7 nm

Stats: Mean +/- Standard Error

Mean:	169.7 +/- 2.9 nm
Mode:	125.3 +/- 9.5 nm
SD:	79.9 +/- 11.1 nm
D10:	96.8 +/- 1.8 nm
D50:	144.5 +/- 4.8 nm
D90:	251.4 +/- 10.9 nm
Concentration:	2.13e+008 +/- 2.54e+007 particles/ml
	10.8 +/- 1.3 particles/frame
	14.8 +/- 1.1 centres/frame

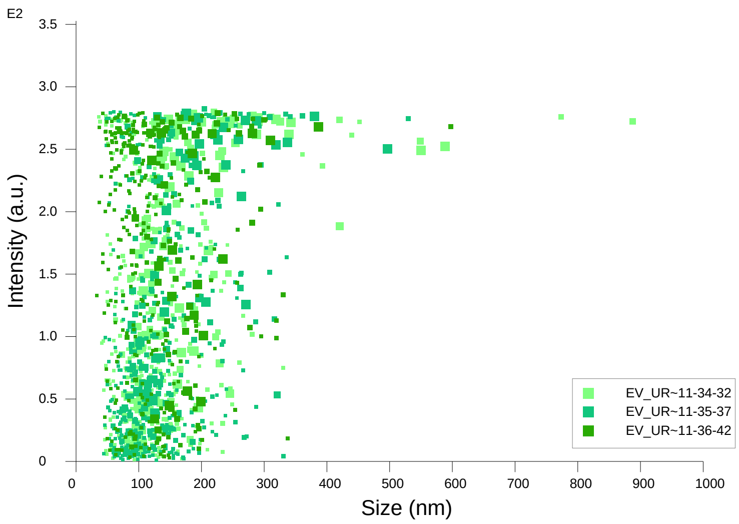

Intensity / Size graph for Experiment:
EV_URINE_S-O_PZ3 2021-09-03 11-34-22

Script Used: (Full Text):

SOP Standard Measurement 11-34-22AM 03Sep2021.txt
