# Supplementary material for: Salting-Out Approach Is Worthy of Comparison with Ultracentrifugation for Extracellular Vesicle Isolation from Tumor and Healthy Models
Source: Biomolecules. 2021 Dec 10;11(12):1857. doi: 10.3390/biom11121857 (PMC8699204; doi:10.3390/biom11121857)
Supplement: Supplementary file 1 [file biomolecules-11-01857-s001.zip › Figure S26.pdf]

NANOSIGHT

EV_URINE_ULTRA_PZ3 2021-09-03 11-44-07

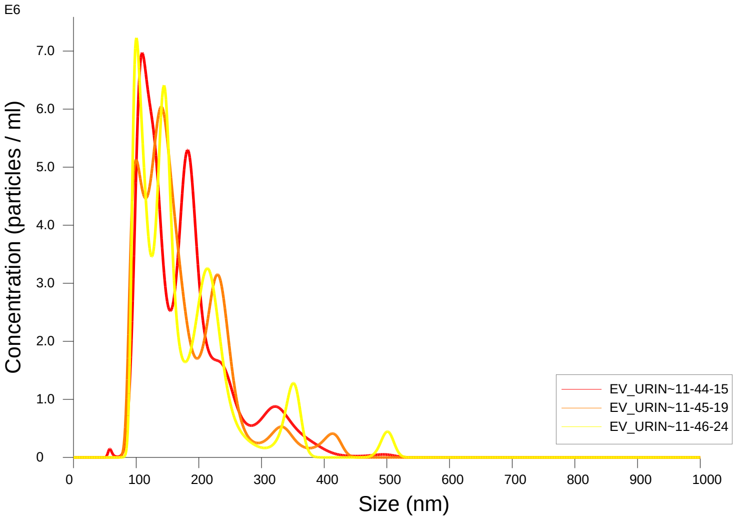

FTLA Concentration / Size graph for Experiment:
EV_URINE_ULTRA_PZ3 2021-09-03 11-44-07

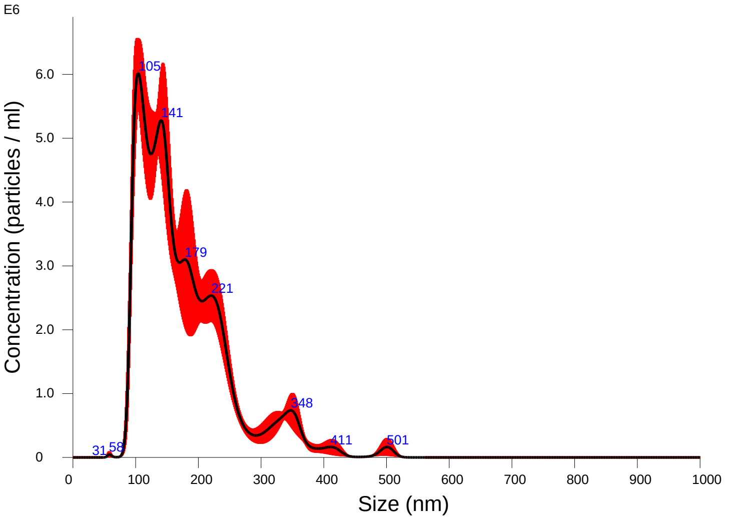

Averaged FTLA Concentration / Size for Experiment:
EV_URINE_ULTRA_PZ3 2021-09-03 11-44-07
Error bars indicate + / -1 standard error of the mean

Included Files

EV_URINE_ULTRA_PZ3 2021-09-03 11-44-15
EV_URINE_ULTRA_PZ3 2021-09-03 11-45-19
EV_URINE_ULTRA_PZ3 2021-09-03 11-46-24

Details

NTA Version:	NTA 3.2 Dev Build 3.2.16
Script Used:	SOP Standard Measurement 11-44-07AM 03~
Time Captured:	11:44:07 03/09/2021
Operator:	simo
Pre-treatment:	
Sample Name:	EV_URINE_ULTRA_PZ3
Diluent:	
Remarks:	1:1000

Capture Settings

Camera Type:	sCMOS
Laser Type:	Green
Camera Level:	14
Slider Shutter:	1259
Slider Gain:	366
FPS	25.0
Number of Frames:	1498
Temperature:	24.1 - 24.2 ^{o}C
Viscosity:	(Water) 0.904 - 0.907 cP
Dilution factor:	Dilution not recorded
Syringe Pump Speed:	50

Analysis Settings

Detect Threshold:	5
Blur Size:	Auto
Max Jump Distance:	Auto: 11.2 - 12.1 pix

Results

Stats: Merged Data

Mean:	175.7 nm
Mode:	104.0 nm
SD:	74.0 nm
D10:	101.7 nm
D50:	153.9 nm
D90:	265.9 nm

Stats: Mean +/- Standard Error

Mean:	175.7 +/- 1.1 nm
Mode:	116.7 +/- 12.0 nm
SD:	74.0 +/- 3.0 nm
D10:	101.7 +/- 1.4 nm
D50:	155.3 +/- 4.3 nm
D90:	268.8 +/- 9.1 nm
Concentration:	6.65e+008 +/- 1.54e+007 particles/ml
	33.7 +/- 0.8 particles/frame
	37.6 +/- 1.0 centres/frame

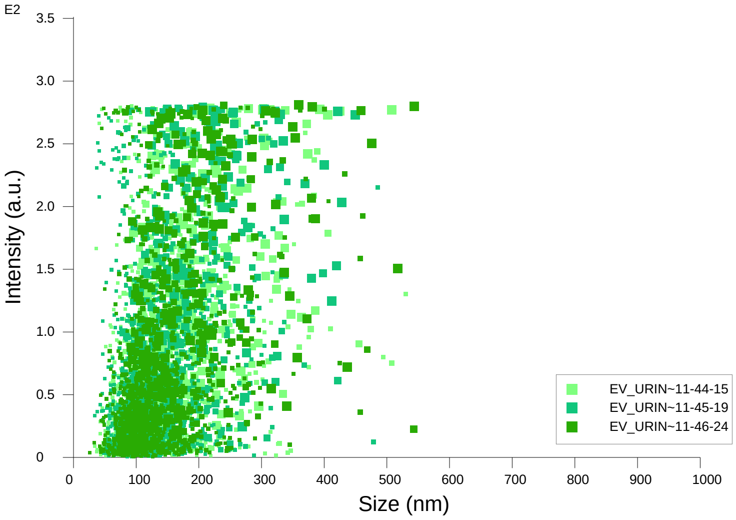

Intensity / Size graph for Experiment:
EV_URINE_ULTRA_PZ3 2021-09-03 11-44-07

Script Used: (Full Text):

SOP Standard Measurement 11-44-07AM 03Sep2021.txt
