# Supplementary material for: Salting-Out Approach Is Worthy of Comparison with Ultracentrifugation for Extracellular Vesicle Isolation from Tumor and Healthy Models
Source: Biomolecules. 2021 Dec 10;11(12):1857. doi: 10.3390/biom11121857 (PMC8699204; doi:10.3390/biom11121857)
Supplement: Supplementary file 1 [file biomolecules-11-01857-s001.zip › Figure S27.pdf]

NANOSIGHT

Urine_NaAC_Centr 2019-11-19 15-33-56

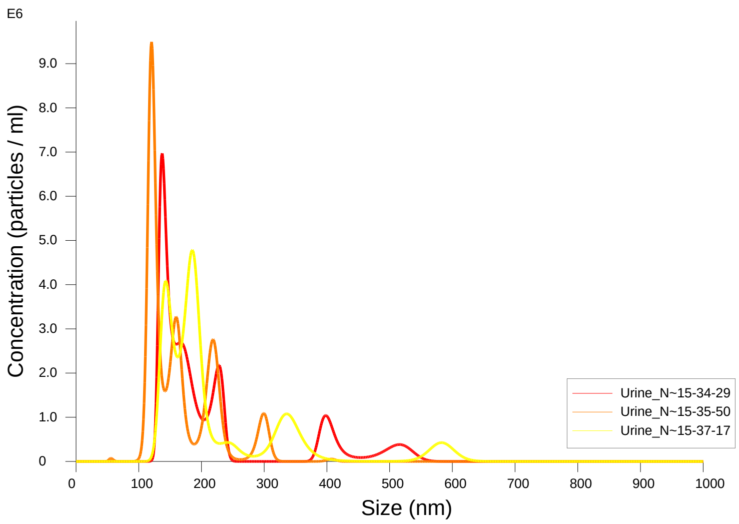

FTLA Concentration / Size graph for Experiment:
Urine_NaAC_Centr 2019-11-19 15-33-56

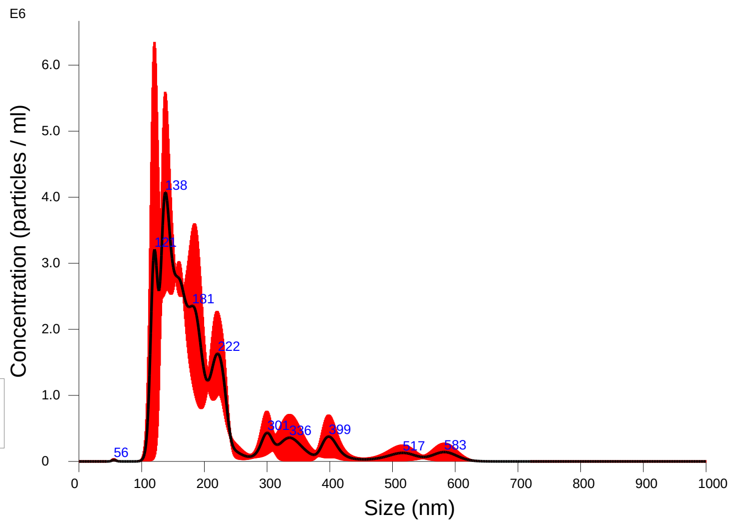

Averaged FTLA Concentration / Size for Experiment:
Urine_NaAC_Centr 2019-11-19 15-33-56
Error bars indicate + / -1 standard error of the mean

Included Files

Urine_NaAC_Centr 2019-11-19 15-34-29
Urine_NaAC_Centr 2019-11-19 15-35-50
Urine_NaAC_Centr 2019-11-19 15-37-17

Details

NTA Version:	NTA 3.2 Dev Build 3.2.16
Script Used:	SOP Standard Measurement 03-33-56PM 19~
Time Captured:	15:33:56 19/11/2019
Operator:	Antonio
Pre-treatment:	
Sample Name:	Urine_NaAC_Centr
Diluent:	
Remarks:	

Capture Settings

Camera Type:	sCMOS
Laser Type:	Green
Camera Level:	14
Slider Shutter:	1259
Slider Gain:	366
FPS	25.0
Number of Frames:	1498
Temperature:	28.0 - 28.0 ^{o}C
Viscosity:	(Water) 0.831 - 0.832 cP
Dilution factor:	Dilution not recorded

Analysis Settings

Detect Threshold:	9
Blur Size:	Auto
Max Jump Distance:	Auto: 9.4 - 10.8 pix

Results

Stats: Merged Data

Mean:	202.0 nm
Mode:	138.0 nm
SD:	99.7 nm
D10:	122.9 nm
D50:	168.5 nm
D90:	339.8 nm

Stats: Mean +/- Standard Error

Mean:	202.0 +/- 18.6 nm
Mode:	147.6 +/- 19.5 nm
SD:	92.4 +/- 18.9 nm
D10:	129.7 +/- 7.4 nm
D50:	166.4 +/- 10.7 nm
D90:	330.2 +/- 52.5 nm
Concentration:	3.52e+008 +/- 6.90e+006 particles/ml
	17.9 +/- 0.4 particles/frame
	23.7 +/- 0.5 centres/frame

NANOSIGHT

Urine_NaAC_Centr 2019-11-19 15-33-56

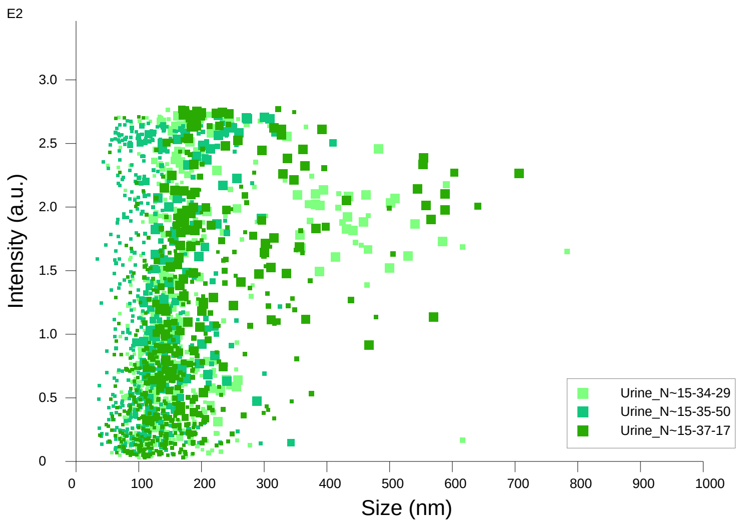

Intensity / Size graph for Experiment:
Urine_NaAC_Centr 2019-11-19 15-33-56

Script Used: (Full Text):

SOP Standard Measurement 03-33-56PM 19Nov2019.txt
