# Supplementary material for: Salting-Out Approach Is Worthy of Comparison with Ultracentrifugation for Extracellular Vesicle Isolation from Tumor and Healthy Models
Source: Biomolecules. 2021 Dec 10;11(12):1857. doi: 10.3390/biom11121857 (PMC8699204; doi:10.3390/biom11121857)
Supplement: Supplementary file 1 [file biomolecules-11-01857-s001.zip › Figure S28.pdf]

NANOSIGHT

Urine_NaAC_Centr 2019-11-19 15-14-26

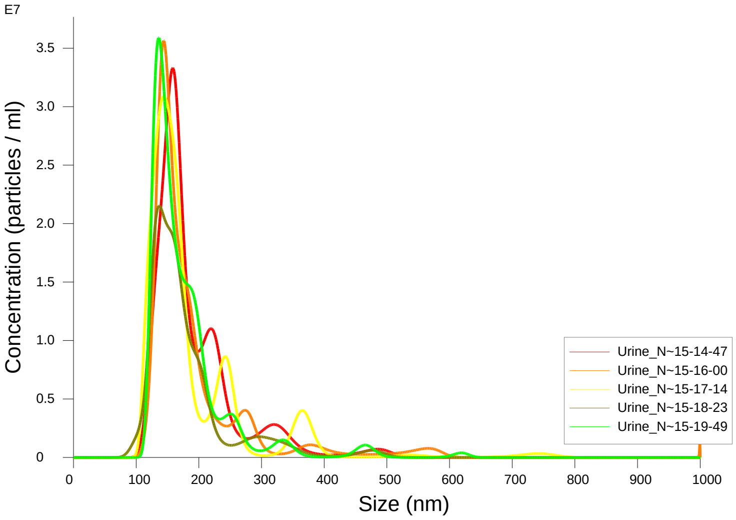

FTLA Concentration / Size graph for Experiment:
Urine_NaAC_Centr 2019-11-19 15-14-26

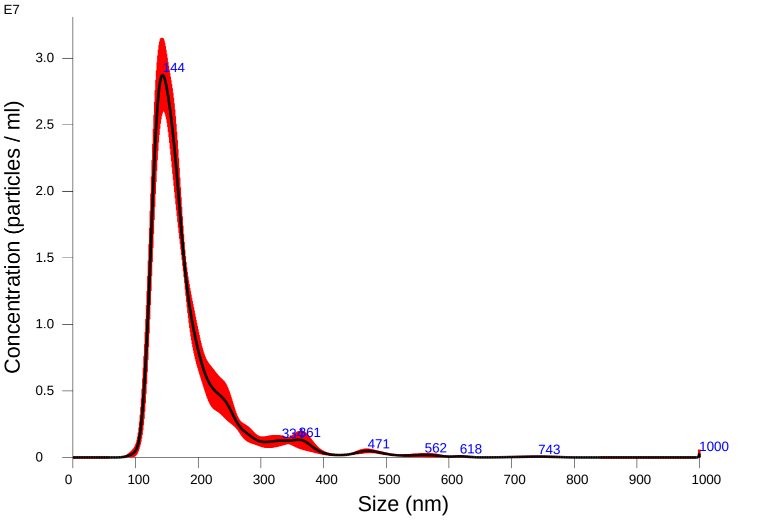

Averaged FTLA Concentration / Size for Experiment:
Urine_NaAC_Centr 2019-11-19 15-14-26
Error bars indicate + / -1 standard error of the mean

Included Files

Urine_NaAC_Centr 2019-11-19 15-14-47
Urine_NaAC_Centr 2019-11-19 15-16-00
Urine_NaAC_Centr 2019-11-19 15-17-14
Urine_NaAC_Centr 2019-11-19 15-18-23
Urine_NaAC_Centr 2019-11-19 15-19-49

Details

NTA Version:	NTA 3.2 Dev Build 3.2.16
Script Used:	SOP Standard Measurement 03-14-26PM 19~
Time Captured:	15:14:26 19/11/2019
Operator:	Antonio
Pre-treatment:	
Sample Name:	Urine_NaAC_Centr
Diluent:	
Remarks:	

Capture Settings

Camera Type:	sCMOS
Laser Type:	Green
Camera Level:	12
Slider Shutter:	1200
Slider Gain:	146
FPS	25.0
Number of Frames:	1498
Temperature:	29.3 - 29.6 ^{o}C
Viscosity:	(Water) 0.803 - 0.809 cP
Dilution factor:	Dilution not recorded

Analysis Settings

Detect Threshold:	8
Blur Size:	Auto
Max Jump Distance:	Auto: 11.2 - 11.8 pix

Results

Stats: Merged Data

Mean:	185.8 nm
Mode:	143.1 nm
SD:	77.9 nm
D10:	127.6 nm
D50:	160.5 nm
D90:	266.5 nm

Stats: Mean +/- Standard Error

Mean:	185.4 +/- 2.8 nm
Mode:	143.8 +/- 4.0 nm
SD:	76.7 +/- 5.2 nm
D10:	127.8 +/- 2.1 nm
D50:	160.0 +/- 2.0 nm
D90:	267.6 +/- 6.0 nm
Concentration:	2.18e+009 +/- 1.16e+008 particles/ml
	110.7 +/- 5.9 particles/frame
	113.6 +/- 5.1 centres/frame

NANOSIGHT

Urine_NaAC_Centr 2019-11-19 15-14-26

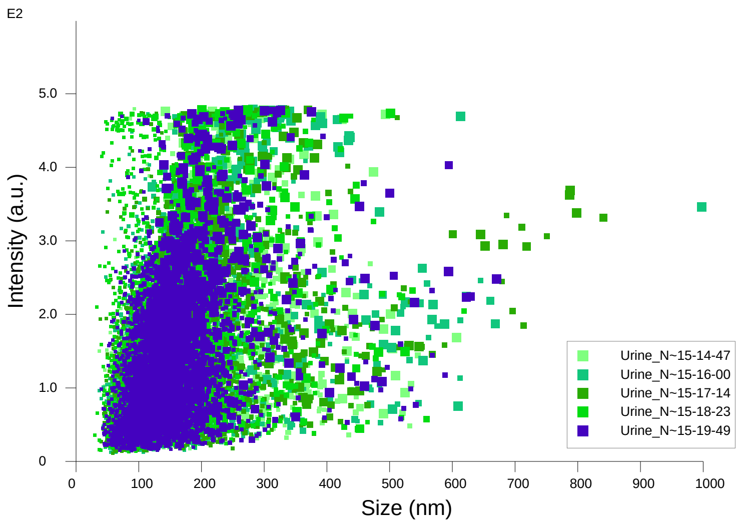

Intensity / Size graph for Experiment:
Urine_NaAC_Centr 2019-11-19 15-14-26

Script Used: (Full Text):

SOP Standard Measurement 03-14-26PM 19Nov2019.txt
