# Supplementary material for: Salting-Out Approach Is Worthy of Comparison with Ultracentrifugation for Extracellular Vesicle Isolation from Tumor and Healthy Models
Source: Biomolecules. 2021 Dec 10;11(12):1857. doi: 10.3390/biom11121857 (PMC8699204; doi:10.3390/biom11121857)
Supplement: Supplementary file 1 [file biomolecules-11-01857-s001.zip › Figure S29.pdf]

NANOSIGHT

8701BC_NaAC 2019-10-23 10-32-17

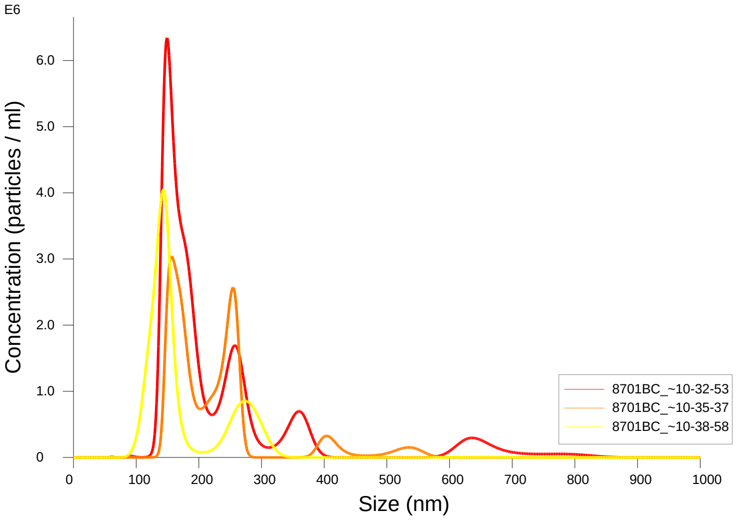

FTLA Concentration / Size graph for Experiment:
8701BC_NaAC 2019-10-23 10-32-17

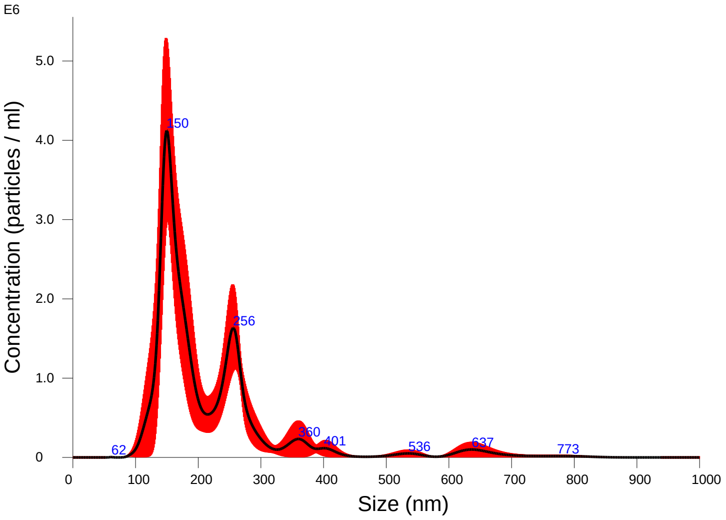

Averaged FTLA Concentration / Size for Experiment:
8701BC_NaAC 2019-10-23 10-32-17
Error bars indicate + / -1 standard error of the mean

Included Files

8701BC_NaAC 2019-10-23 10-32-53
8701BC_NaAC 2019-10-23 10-35-37
8701BC_NaAC 2019-10-23 10-38-58

Details

NTA Version:	NTA 3.2 Dev Build 3.2.16
Script Used:	SOP Standard Measurement 10-32-17AM 23O~
Time Captured:	10:32:17 23/10/2019
Operator:	Antonio
Pre-treatment:	
Sample Name:	8701BC_NaAC
Diluent:	
Remarks:	

Capture Settings

Camera Type:	sCMOS
Laser Type:	Green
Camera Level:	12 - 14
Slider Shutter:	1200 - 1259
Slider Gain:	146 - 366
FPS	25.0
Number of Frames:	1498
Temperature:	22.3 - 22.7 ^{o}C
Viscosity:	(Water) 0.938 - 0.947 cP
Dilution factor:	Dilution not recorded

Analysis Settings

Detect Threshold:	14
Blur Size:	Auto
Max Jump Distance:	Auto: 9.0 - 10.4 pix

Results

Stats: Merged Data

Mean:	216.6 nm
Mode:	149.9 nm
SD:	111.4 nm
D10:	137.6 nm
D50:	174.5 nm
D90:	312.6 nm

Stats: Mean +/- Standard Error

Mean:	211.4 +/- 18.3 nm
Mode:	149.7 +/- 3.8 nm
SD:	96.0 +/- 20.5 nm
D10:	138.4 +/- 9.8 nm
D50:	176.7 +/- 16.7 nm
D90:	304.2 +/- 28.7 nm
Concentration:	2.86e+008 +/- 6.05e+007 particles/ml
	14.5 +/- 3.1 particles/frame
	16.5 +/- 3.8 centres/frame

8701BC_NaAC 2019-10-23 10-32-17

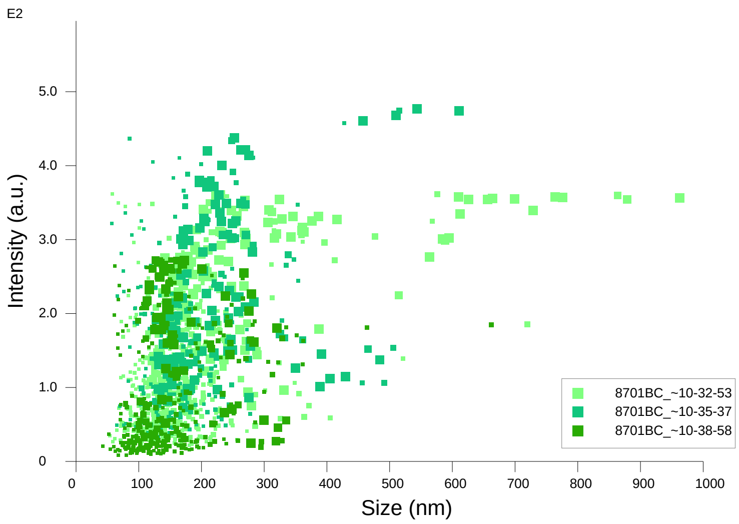

Intensity / Size graph for Experiment:
8701BC_NaAC 2019-10-23 10-32-17

Script Used: (Full Text):

SOP Standard Measurement 10-32-17AM 23Oct2019.txt
