# Supplementary material for: Salting-Out Approach Is Worthy of Comparison with Ultracentrifugation for Extracellular Vesicle Isolation from Tumor and Healthy Models
Source: Biomolecules. 2021 Dec 10;11(12):1857. doi: 10.3390/biom11121857 (PMC8699204; doi:10.3390/biom11121857)
Supplement: Supplementary file 1 [file biomolecules-11-01857-s001.zip › Figure S30.pdf]

NANOSIGHT

8701 bc_U 2019-11-21 11-22-08

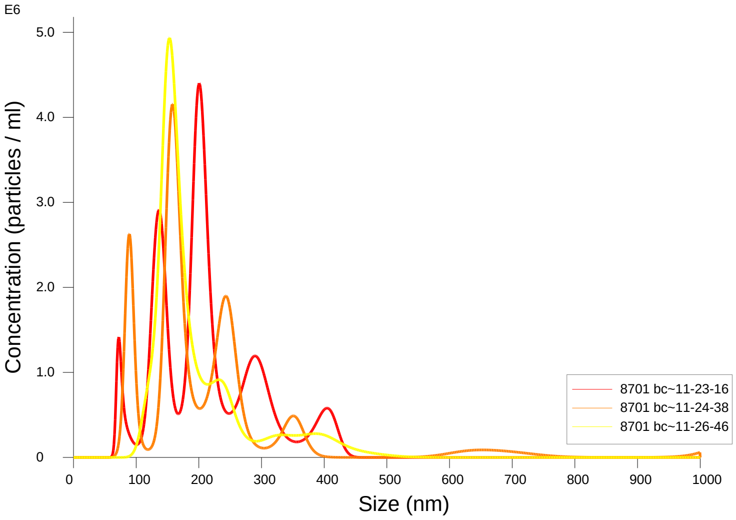

FTLA Concentration / Size graph for Experiment:
8701 bc_U 2019-11-21 11-22-08

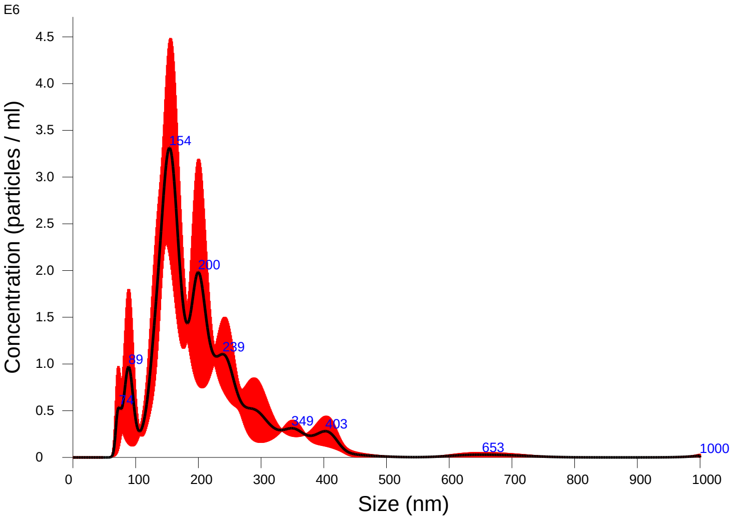

Averaged FTLA Concentration / Size for Experiment:
8701 bc_U 2019-11-21 11-22-08
Error bars indicate + / -1 standard error of the mean

Included Files

8701 bc_U 2019-11-21 11-23-16
8701 bc_U 2019-11-21 11-24-38
8701 bc_U 2019-11-21 11-26-46

Details

NTA Version:	NTA 3.2 Dev Build 3.2.16
Script Used:	SOP Standard Measurement 11-22-08AM 21~
Time Captured:	11:22:08 21/11/2019
Operator:	Antonio
Pre-treatment:	
Sample Name:	8701 bc_U
Diluent:	
Remarks:	

Capture Settings

Camera Type:	sCMOS
Laser Type:	Green
Camera Level:	12
Slider Shutter:	1200
Slider Gain:	146
FPS	25.0
Number of Frames:	1498
Temperature:	21.2 - 21.4 ^{o}C
Viscosity:	(Water) 0.966 - 0.971 cP
Dilution factor:	Dilution not recorded

Analysis Settings

Detect Threshold:	11
Blur Size:	Auto
Max Jump Distance:	Auto: 9.3 - 10.9 pix

Results

Stats: Merged Data

Mean:	207.0 nm
Mode:	154.0 nm
SD:	97.3 nm
D10:	123.5 nm
D50:	182.2 nm
D90:	325.9 nm

Stats: Mean +/- Standard Error

Mean:	206.7 +/- 4.8 nm
Mode:	170.6 +/- 15.1 nm
SD:	95.5 +/- 15.4 nm
D10:	116.6 +/- 13.0 nm
D50:	179.0 +/- 10.8 nm
D90:	326.5 +/- 2.6 nm
Concentration:	3.36e+008 +/- 1.68e+007 particles/ml
	17.0 +/- 0.9 particles/frame
	23.5 +/- 1.3 centres/frame

NANOSIGHT

8701 bc_U 2019-11-21 11-22-08

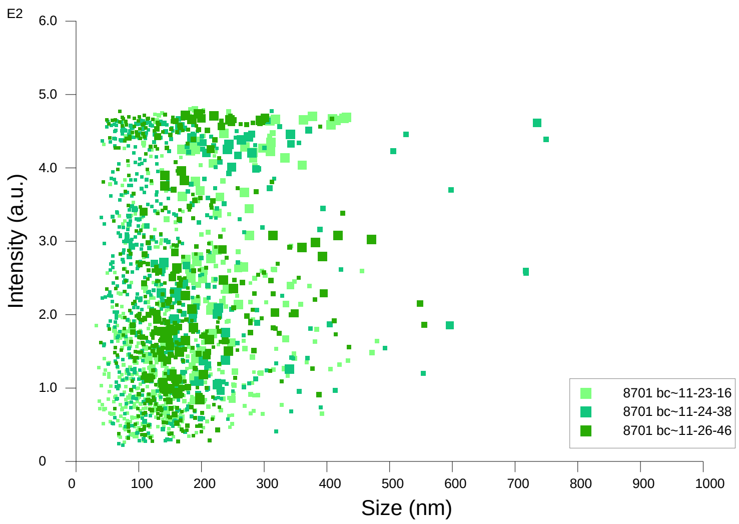

Intensity / Size graph for Experiment:
8701 bc_U 2019-11-21 11-22-08

Script Used: (Full Text):

SOP Standard Measurement 11-22-08AM 21Nov2019.txt
