# Supplementary material for: Salting-Out Approach Is Worthy of Comparison with Ultracentrifugation for Extracellular Vesicle Isolation from Tumor and Healthy Models
Source: Biomolecules. 2021 Dec 10;11(12):1857. doi: 10.3390/biom11121857 (PMC8699204; doi:10.3390/biom11121857)
Supplement: Supplementary file 1 [file biomolecules-11-01857-s001.zip › Figure S31.pdf]

Batteri LE392 Ultra 2020-02-17 14-43-44

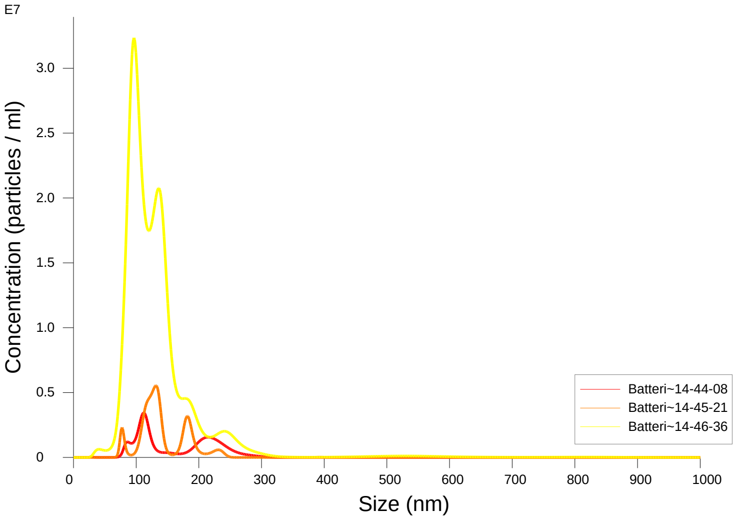

FTLA Concentration / Size graph for Experiment:
Batteri LE392 Ultra 2020-02-17 14-43-44

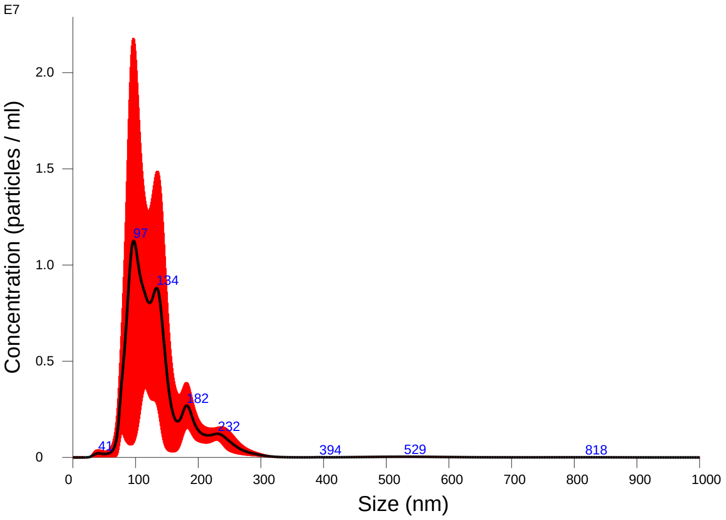

Averaged FTLA Concentration / Size for Experiment:
Batteri LE392 Ultra 2020-02-17 14-43-44
Error bars indicate + / -1 standard error of the mean

Included Files

Batteri LE392 Ultra 2020-02-17 14-44-08
Batteri LE392 Ultra 2020-02-17 14-45-21
Batteri LE392 Ultra 2020-02-17 14-46-36

Details

NTA Version:	NTA 3.2 Dev Build 3.2.16
Script Used:	SOP Standard Measurement 02-43-44PM 17~
Time Captured:	14:43:44 17/02/2020
Operator:	Antonio
Pre-treatment:	
Sample Name:	Batteri LE392 Ultra
Diluent:	
Remarks:	

Capture Settings

Camera Type:	sCMOS
Laser Type:	Green
Camera Level:	13 - 14
Slider Shutter:	1232 - 1259
Slider Gain:	219 - 366
FPS	25.0
Number of Frames:	1498
Temperature:	22.0 ^{o}C
Viscosity:	(Water) 1.0 cP
Dilution factor:	Dilution not recorded

Analysis Settings

Detect Threshold:	5
Blur Size:	Auto
Max Jump Distance:	Auto: 11.9 - 14.4 pix

Results

Stats: Merged Data

Mean:	135.8 nm
Mode:	97.0 nm
SD:	63.2 nm
D10:	86.2 nm
D50:	121.0 nm
D90:	201.5 nm

Stats: Mean +/- Standard Error

Mean:	145.8 +/- 9.3 nm
Mode:	113.4 +/- 10.1 nm
SD:	54.6 +/- 8.1 nm
D10:	94.6 +/- 5.1 nm
D50:	131.3 +/- 8.3 nm
D90:	206.1 +/- 17.4 nm
Concentration:	8.32e+008 +/- 5.93e+008 particles/ml
	42.2 +/- 30.1 particles/frame
	60.7 +/- 47.3 centres/frame

NANOSIGHT

Batteri LE392 Ultra 2020-02-17 14-43-44

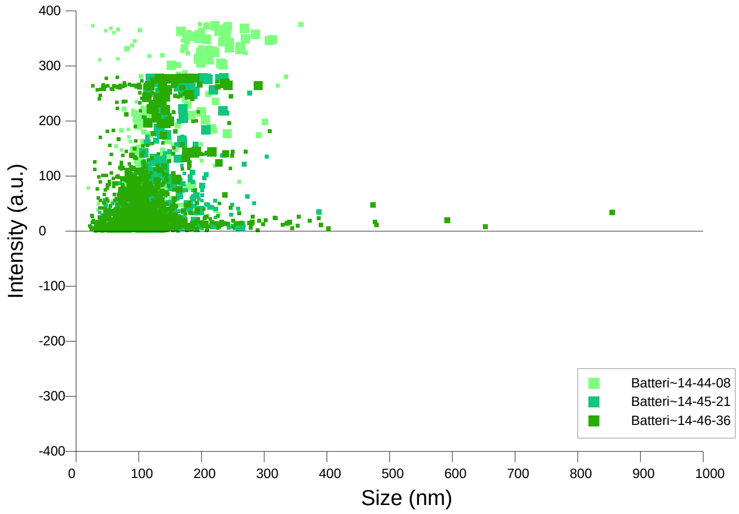

Intensity / Size graph for Experiment:
Batteri LE392 Ultra 2020-02-17 14-43-44

Script Used: (Full Text):

SOP Standard Measurement 02-43-44PM 17Feb2020.txt
