# Supplementary material for: Salting-Out Approach Is Worthy of Comparison with Ultracentrifugation for Extracellular Vesicle Isolation from Tumor and Healthy Models
Source: Biomolecules. 2021 Dec 10;11(12):1857. doi: 10.3390/biom11121857 (PMC8699204; doi:10.3390/biom11121857)
Supplement: Supplementary file 1 [file biomolecules-11-01857-s001.zip › Figure S32.pdf]

NANOSIGHT

Hb2_2 NaAc 2020-02-17 14-32-09

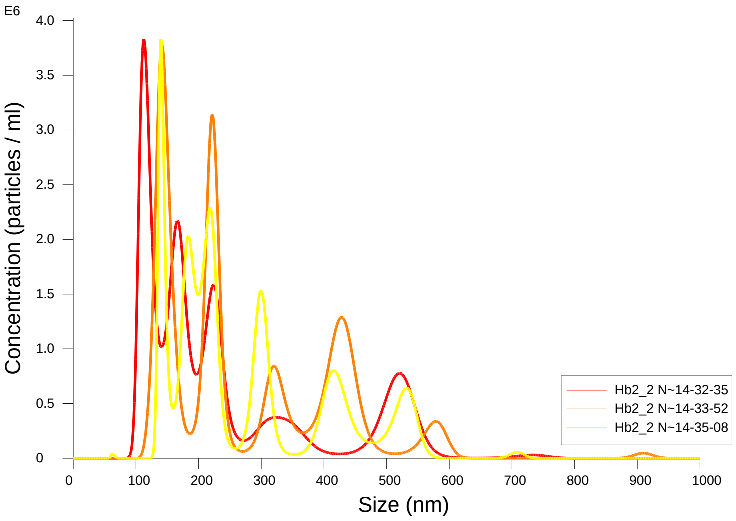

FTLA Concentration / Size graph for Experiment:
Hb2_2 NaAc 2020-02-17 14-32-09

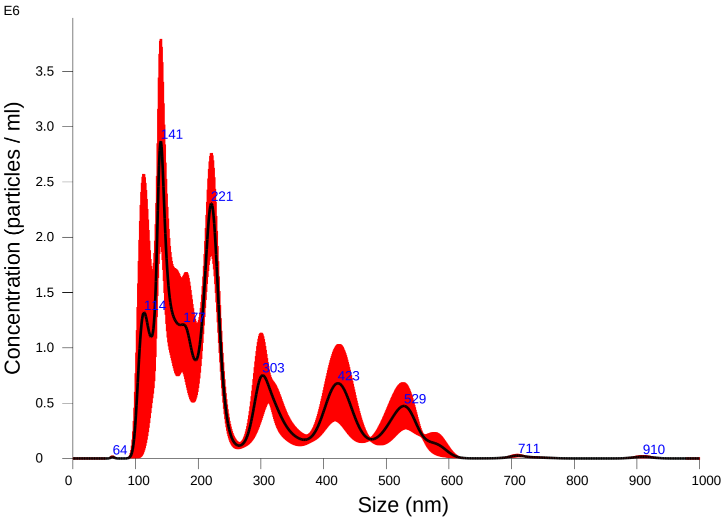

Averaged FTLA Concentration / Size for Experiment:
Hb2_2 NaAc 2020-02-17 14-32-09
Error bars indicate + / -1 standard error of the mean

Included Files

Hb2_2 NaAc 2020-02-17 14-32-35
Hb2_2 NaAc 2020-02-17 14-33-52
Hb2_2 NaAc 2020-02-17 14-35-08

Details

NTA Version:	NTA 3.2 Dev Build 3.2.16
Script Used:	SOP Standard Measurement 02-32-09PM 17~
Time Captured:	14:32:09 17/02/2020
Operator:	Antonio
Pre-treatment:	
Sample Name:	Hb2_2 NaAc
Diluent:	
Remarks:	

Capture Settings

Camera Type:	sCMOS
Laser Type:	Green
Camera Level:	12
Slider Shutter:	1200
Slider Gain:	146
FPS	25.0
Number of Frames:	1498
Temperature:	22.0 ^{o}C
Viscosity:	(Water) 1.0 cP
Dilution factor:	Dilution not recorded

Analysis Settings

Detect Threshold:	5
Blur Size:	Auto
Max Jump Distance:	Auto: 9.6 - 11.1 pix

Results

Stats: Merged Data

Mean:	262.9 nm
Mode:	140.2 nm
SD:	137.6 nm
D10:	128.2 nm
D50:	216.1 nm
D90:	487.0 nm

Stats: Mean +/- Standard Error

Mean:	263.1 +/- 12.2 nm
Mode:	131.3 +/- 9.3 nm
SD:	136.1 +/- 4.8 nm
D10:	129.3 +/- 9.6 nm
D50:	208.4 +/- 14.1 nm
D90:	483.5 +/- 19.2 nm
Concentration:	3.26e+008 +/- 1.18e+007 particles/ml
	16.5 +/- 0.6 particles/frame
	20.5 +/- 0.3 centres/frame

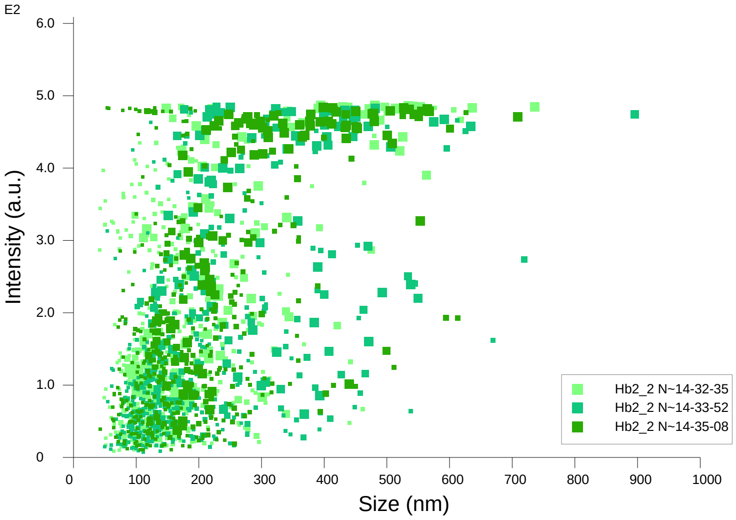

Intensity / Size graph for Experiment:
Hb2_2 NaAc 2020-02-17 14-32-09

Script Used: (Full Text):

SOP Standard Measurement 02-32-09PM 17Feb2020.txt
