# Supplementary material for: Salting-Out Approach Is Worthy of Comparison with Ultracentrifugation for Extracellular Vesicle Isolation from Tumor and Healthy Models
Source: Biomolecules. 2021 Dec 10;11(12):1857. doi: 10.3390/biom11121857 (PMC8699204; doi:10.3390/biom11121857)
Supplement: Supplementary file 1 [file biomolecules-11-01857-s001.zip › Figure S33.pdf]

NANOSIGHT

Hb2_2 Ultra 2020-02-17 14-22-40

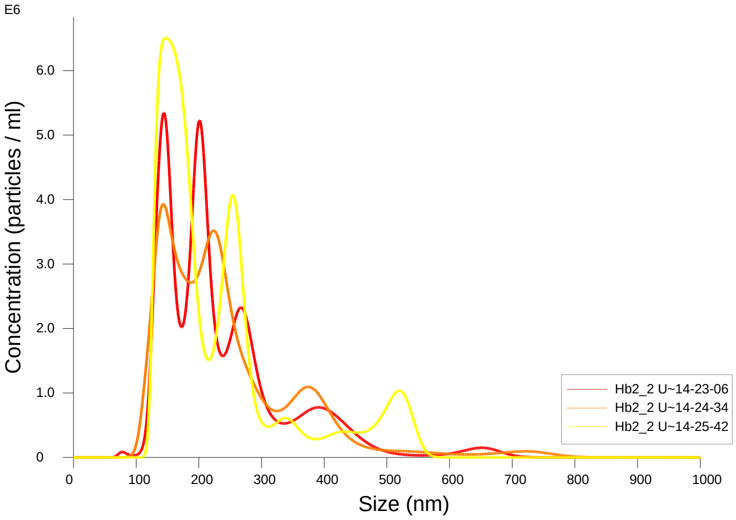

FTLA Concentration / Size graph for Experiment:
Hb2_2 Ultra 2020-02-17 14-22-40

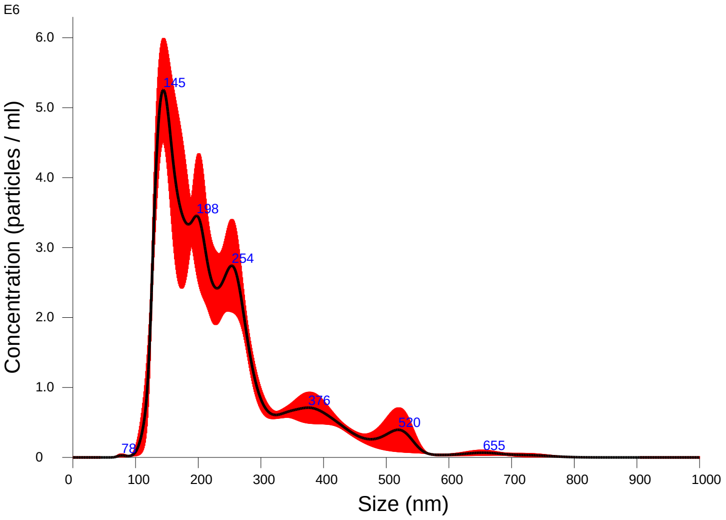

Averaged FTLA Concentration / Size for Experiment:
Hb2_2 Ultra 2020-02-17 14-22-40
Error bars indicate + / -1 standard error of the mean

Included Files

Hb2_2 Ultra 2020-02-17 14-23-06
Hb2_2 Ultra 2020-02-17 14-24-34
Hb2_2 Ultra 2020-02-17 14-25-42

Details

NTA Version:	NTA 3.2 Dev Build 3.2.16
Script Used:	SOP Standard Measurement 02-22-40PM 17~
Time Captured:	14:22:40 17/02/2020
Operator:	Antonio
Pre-treatment:	
Sample Name:	Hb2_2 Ultra
Diluent:	
Remarks:	

Capture Settings

Camera Type:	sCMOS
Laser Type:	Green
Camera Level:	12
Slider Shutter:	1200
Slider Gain:	146
FPS	25.0
Number of Frames:	1498
Temperature:	22.0 ^{o}C
Viscosity:	(Water) 1.0 cP
Dilution factor:	Dilution not recorded

Analysis Settings

Detect Threshold:	5
Blur Size:	Auto
Max Jump Distance:	Auto: 9.8 - 10.5 pix

Results

Stats: Merged Data

Mean:	238.3 nm
Mode:	144.3 nm
SD:	109.0 nm
D10:	137.2 nm
D50:	205.9 nm
D90:	395.3 nm

Stats: Mean +/- Standard Error

Mean:	238.5 +/- 2.2 nm
Mode:	144.9 +/- 1.3 nm
SD:	108.8 +/- 2.4 nm
D10:	137.0 +/- 1.0 nm
D50:	204.6 +/- 7.0 nm
D90:	403.1 +/- 16.1 nm
Concentration:	6.80e+008 +/- 3.82e+007 particles/ml
	34.5 +/- 1.9 particles/frame
	40.8 +/- 1.1 centres/frame

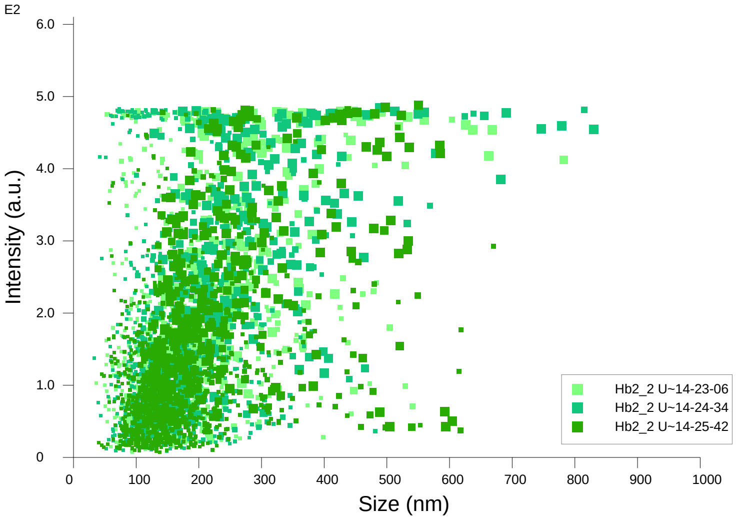

Intensity / Size graph for Experiment:
Hb2_2 Ultra 2020-02-17 14-22-40

Script Used: (Full Text):

SOP Standard Measurement 02-22-40PM 17Feb2020.txt
