# Supplementary material for: Salting-Out Approach Is Worthy of Comparison with Ultracentrifugation for Extracellular Vesicle Isolation from Tumor and Healthy Models
Source: Biomolecules. 2021 Dec 10;11(12):1857. doi: 10.3390/biom11121857 (PMC8699204; doi:10.3390/biom11121857)
Supplement: Supplementary file 1 [file biomolecules-11-01857-s001.zip › Figure S34.pdf]

NANOSIGHT

Caco2_NaAc 2019-11-19 12-38-11

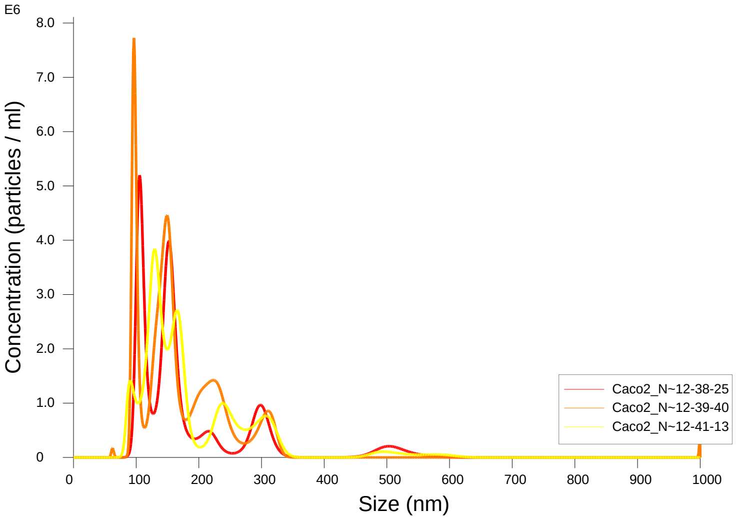

FTLA Concentration / Size graph for Experiment:
Caco2_NaAc 2019-11-19 12-38-11

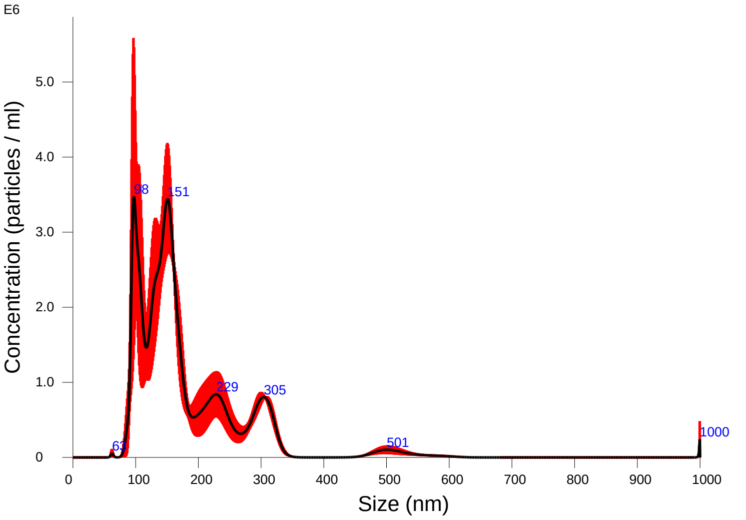

Averaged FTLA Concentration / Size for Experiment:
Caco2_NaAc 2019-11-19 12-38-11
Error bars indicate + / -1 standard error of the mean

Included Files

Caco2_NaAc 2019-11-19 12-38-25
Caco2_NaAc 2019-11-19 12-39-40
Caco2_NaAc 2019-11-19 12-41-13

Details

NTA Version:	NTA 3.2 Dev Build 3.2.16
Script Used:	SOP Standard Measurement 12-38-11PM 19~
Time Captured:	12:38:11 19/11/2019
Operator:	Antonio
Pre-treatment:	
Sample Name:	Caco2_NaAc
Diluent:	
Remarks:	

Capture Settings

Camera Type:	sCMOS
Laser Type:	Green
Camera Level:	14
Slider Shutter:	1259
Slider Gain:	366
FPS	25.0
Number of Frames:	1498
Temperature:	23.0 ^{o}C
Viscosity:	(Water) 0.9 cP
Dilution factor:	Dilution not recorded

Analysis Settings

Detect Threshold:	5
Blur Size:	Auto
Max Jump Distance:	Auto: 11.2 - 11.9 pix

Results

Stats: Merged Data

Mean:	178.6 nm
Mode:	97.3 nm
SD:	88.8 nm
D10:	99.1 nm
D50:	151.1 nm
D90:	295.0 nm

Stats: Mean +/- Standard Error

Mean:	179.0 +/- 4.7 nm
Mode:	110.5 +/- 9.9 nm
SD:	89.0 +/- 6.0 nm
D10:	102.0 +/- 3.5 nm
D50:	151.9 +/- 2.1 nm
D90:	288.7 +/- 12.4 nm
Concentration:	3.10e+008 +/- 2.28e+007 particles/ml
	15.7 +/- 1.2 particles/frame
	18.9 +/- 1.5 centres/frame

Caco2_NaAc 2019-11-19 12-38-11

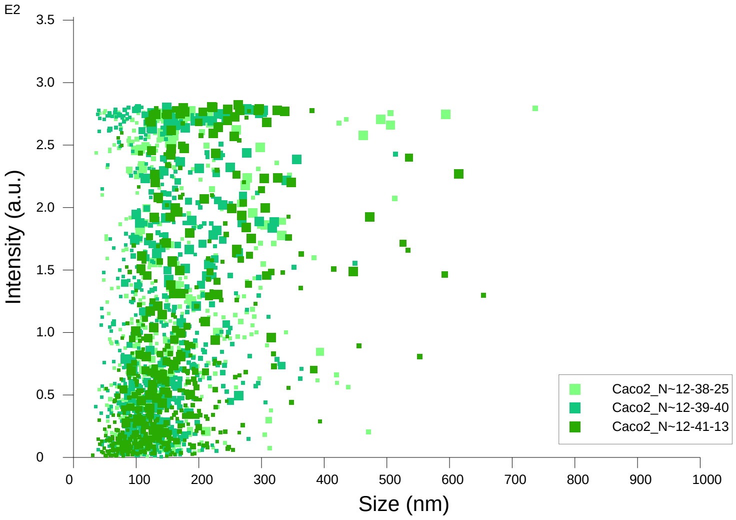

Intensity / Size graph for Experiment:
Caco2_NaAc 2019-11-19 12-38-11

Script Used: (Full Text):

SOP Standard Measurement 12-38-11PM 19Nov2019.txt
