# Supplementary material for: Salting-Out Approach Is Worthy of Comparison with Ultracentrifugation for Extracellular Vesicle Isolation from Tumor and Healthy Models
Source: Biomolecules. 2021 Dec 10;11(12):1857. doi: 10.3390/biom11121857 (PMC8699204; doi:10.3390/biom11121857)
Supplement: Supplementary file 1 [file biomolecules-11-01857-s001.zip › Figure S35.pdf]

NANOSIGHT

Caco2_U 2019-11-19 13-07-30

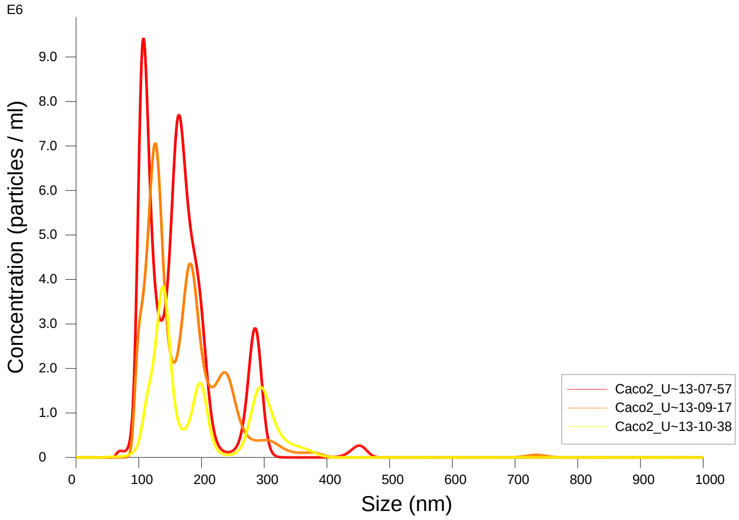

FTLA Concentration / Size graph for Experiment:
Caco2_U 2019-11-19 13-07-30

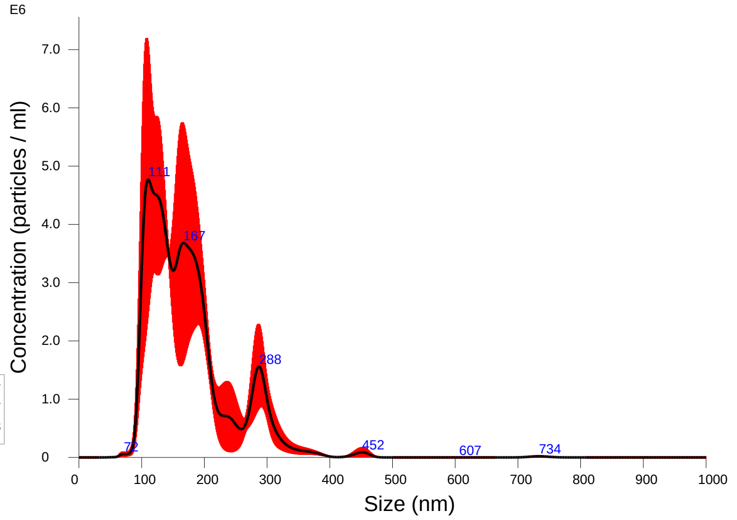

Averaged FTLA Concentration / Size for Experiment:
Caco2_U 2019-11-19 13-07-30
Error bars indicate + / -1 standard error of the mean

Included Files

Caco2_U 2019-11-19 13-07-57
Caco2_U 2019-11-19 13-09-17
Caco2_U 2019-11-19 13-10-38

Details

NTA Version:	NTA 3.2 Dev Build 3.2.16
Script Used:	SOP Standard Measurement 01-07-30PM 19~
Time Captured:	13:07:30 19/11/2019
Operator:	Antonio
Pre-treatment:	
Sample Name:	Caco2_U
Diluent:	
Remarks:	

Capture Settings

Camera Type:	sCMOS
Laser Type:	Green
Camera Level:	14
Slider Shutter:	1259
Slider Gain:	366
FPS	25.0
Number of Frames:	1498
Temperature:	23.0 ^{o}C
Viscosity:	(Water) 0.9 cP
Dilution factor:	Dilution not recorded

Analysis Settings

Detect Threshold:	5
Blur Size:	Auto
Max Jump Distance:	Auto: 9.9 - 10.6 pix

Results

Stats: Merged Data

Mean:	173.9 nm
Mode:	110.3 nm
SD:	67.3 nm
D10:	106.7 nm
D50:	158.8 nm
D90:	279.7 nm

Stats: Mean +/- Standard Error

Mean:	178.6 +/- 9.5 nm
Mode:	124.2 +/- 9.1 nm
SD:	68.3 +/- 4.2 nm
D10:	111.0 +/- 4.9 nm
D50:	160.9 +/- 2.7 nm
D90:	276.4 +/- 18.4 nm
Concentration:	5.24e+008 +/- 1.32e+008 particles/ml
	26.6 +/- 6.7 particles/frame
	37.0 +/- 9.5 centres/frame

NANOSIGHT

Caco2_U 2019-11-19 13-07-30

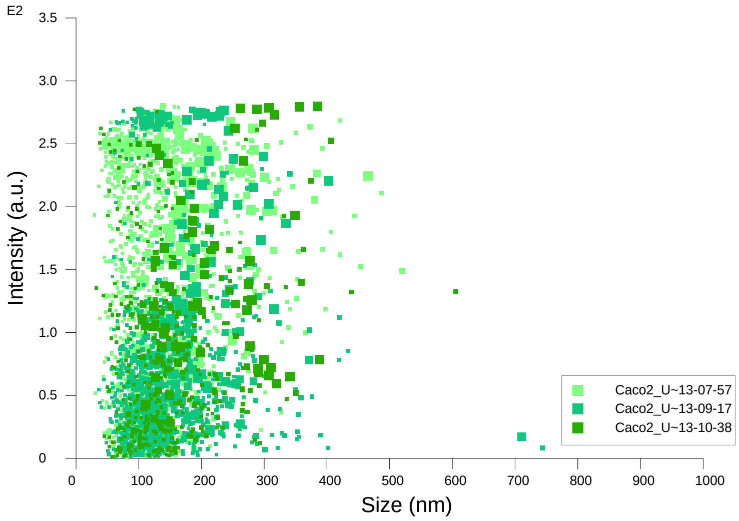

Intensity / Size graph for Experiment:
Caco2_U 2019-11-19 13-07-30

Script Used: (Full Text):

SOP Standard Measurement 01-07-30PM 19Nov2019.txt
