# Supplementary material for: Salting-Out Approach Is Worthy of Comparison with Ultracentrifugation for Extracellular Vesicle Isolation from Tumor and Healthy Models
Source: Biomolecules. 2021 Dec 10;11(12):1857. doi: 10.3390/biom11121857 (PMC8699204; doi:10.3390/biom11121857)
Supplement: Supplementary file 1 [file biomolecules-11-01857-s001.zip › Figure S36.pdf]

NANOSIGHT

Colo205_NaAC 2019-10-23 10-15-51

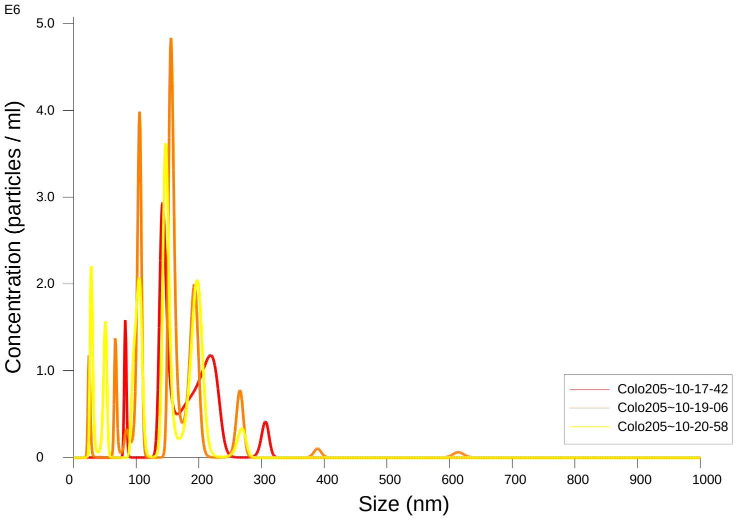

FTLA Concentration / Size graph for Experiment:
Colo205_NaAC 2019-10-23 10-15-51

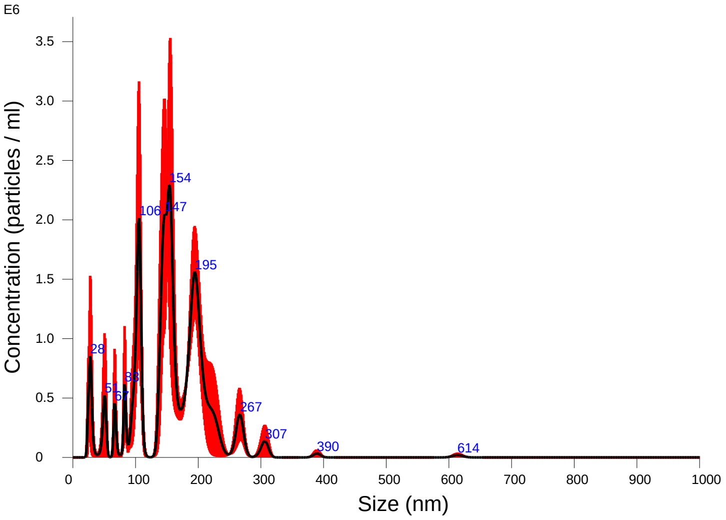

Averaged FTLA Concentration / Size for Experiment:
Colo205_NaAC 2019-10-23 10-15-51
Error bars indicate + / -1 standard error of the mean

Included Files

Colo205_NaAC 2019-10-23 10-17-42
Colo205_NaAC 2019-10-23 10-19-06
Colo205_NaAC 2019-10-23 10-20-58

Details

NTA Version:	NTA 3.2 Dev Build 3.2.16
Script Used:	SOP Standard Measurement 10-15-51AM 23O~
Time Captured:	10:15:51 23/10/2019
Operator:	Antonio
Pre-treatment:	
Sample Name:	Colo205_NaAC
Diluent:	
Remarks:	

Capture Settings

Camera Type:	sCMOS
Laser Type:	Green
Camera Level:	15
Slider Shutter:	1206
Slider Gain:	366
FPS	25.0
Number of Frames:	1498
Temperature:	21.7 - 21.8 ^{o}C
Viscosity:	(Water) 0.957 - 0.961 cP
Dilution factor:	Dilution not recorded

Analysis Settings

Detect Threshold:	9
Blur Size:	Auto
Max Jump Distance:	Auto: 13.3 - 19.5 pix

Results

Stats: Merged Data

Mean:	158.2 nm
Mode:	154.0 nm
SD:	62.7 nm
D10:	84.1 nm
D50:	154.3 nm
D90:	218.6 nm

Stats: Mean +/- Standard Error

Mean:	159.8 +/- 11.2 nm
Mode:	148.2 +/- 3.9 nm
SD:	59.3 +/- 6.4 nm
D10:	91.8 +/- 25.6 nm
D50:	159.5 +/- 8.8 nm
D90:	211.3 +/- 9.0 nm
Concentration:	1.44e+008 +/- 1.16e+007 particles/ml
	7.3 +/- 0.6 particles/frame
	11.6 +/- 2.6 centres/frame

NANOSIGHT

Colo205_NaAC 2019-10-23 10-15-51

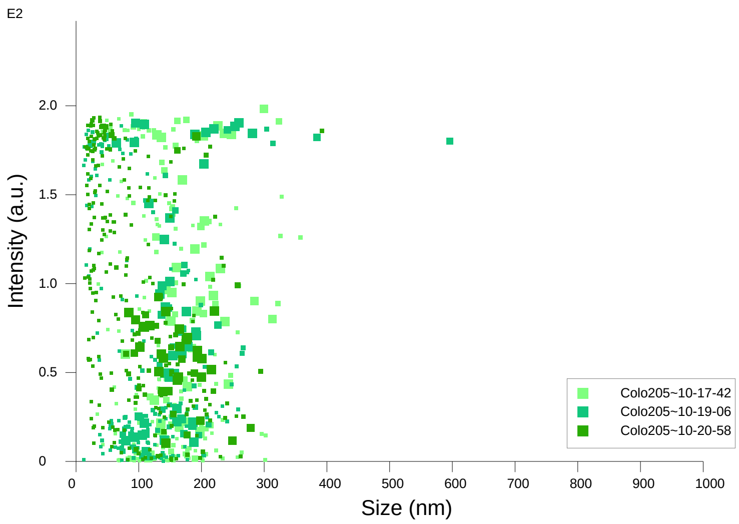

Intensity / Size graph for Experiment:
Colo205_NaAC 2019-10-23 10-15-51

Script Used: (Full Text):

SOP Standard Measurement 10-15-51AM 23Oct2019.txt
