# Supplementary material for: Salting-Out Approach Is Worthy of Comparison with Ultracentrifugation for Extracellular Vesicle Isolation from Tumor and Healthy Models
Source: Biomolecules. 2021 Dec 10;11(12):1857. doi: 10.3390/biom11121857 (PMC8699204; doi:10.3390/biom11121857)
Supplement: Supplementary file 1 [file biomolecules-11-01857-s001.zip › Figure S37.pdf]

NANOSIGHT

COLO205_u 2019-11-21 11-46-02

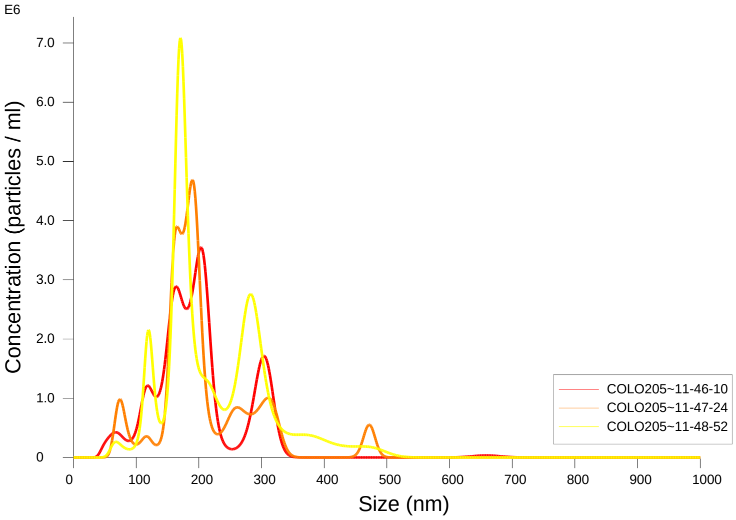

FTLA Concentration / Size graph for Experiment:
COLO205_u 2019-11-21 11-46-02

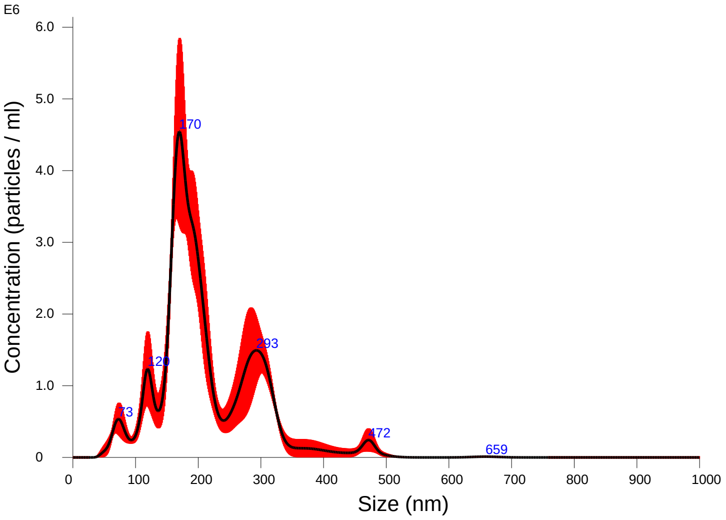

Averaged FTLA Concentration / Size for Experiment:
COLO205_u 2019-11-21 11-46-02
Error bars indicate + / -1 standard error of the mean

Included Files

COLO205_u 2019-11-21 11-46-10
COLO205_u 2019-11-21 11-47-24
COLO205_u 2019-11-21 11-48-52

Details

NTA Version:	NTA 3.2 Dev Build 3.2.16
Script Used:	SOP Standard Measurement 11-46-02AM 21~
Time Captured:	11:46:02 21/11/2019
Operator:	Antonio
Pre-treatment:	
Sample Name:	COLO205_u
Diluent:	
Remarks:	

Capture Settings

Camera Type:	sCMOS
Laser Type:	Green
Camera Level:	12
Slider Shutter:	1200
Slider Gain:	146
FPS	25.0
Number of Frames:	1498
Temperature:	22.4 - 22.6 ^{o}C
Viscosity:	(Water) 0.941 - 0.943 cP
Dilution factor:	Dilution not recorded

Analysis Settings

Detect Threshold:	7
Blur Size:	Auto
Max Jump Distance:	Auto: 11.5 - 13.5 pix

Results

Stats: Merged Data

Mean:	210.6 nm
Mode:	169.9 nm
SD:	79.1 nm
D10:	126.1 nm
D50:	188.6 nm
D90:	308.3 nm

Stats: Mean +/- Standard Error

Mean:	209.0 +/- 7.6 nm
Mode:	187.9 +/- 9.5 nm
SD:	77.6 +/- 3.2 nm
D10:	130.4 +/- 7.3 nm
D50:	189.1 +/- 1.3 nm
D90:	310.9 +/- 7.2 nm
Concentration:	4.03e+008 +/- 4.42e+007 particles/ml
	20.5 +/- 2.2 particles/frame
	27.8 +/- 1.8 centres/frame

NANOSIGHT

COLO205_u 2019-11-21 11-46-02

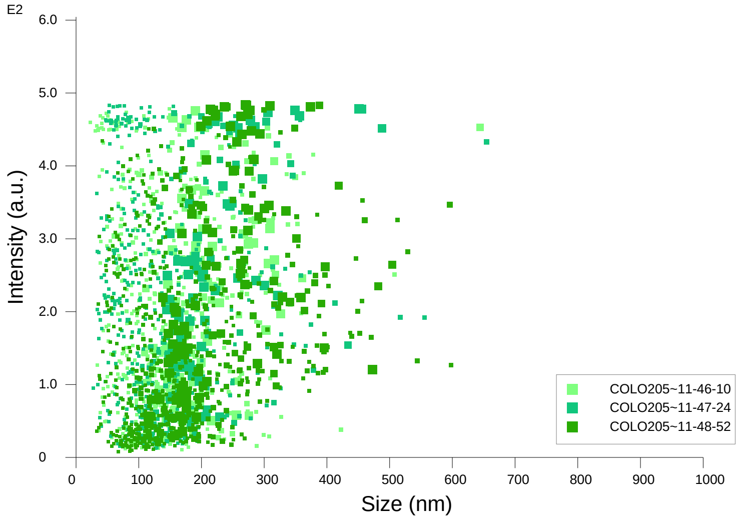

Intensity / Size graph for Experiment:
COLO205_u 2019-11-21 11-46-02

Script Used: (Full Text):

SOP Standard Measurement 11-46-02AM 21Nov2019.txt
