# Supplementary material for: Salting-Out Approach Is Worthy of Comparison with Ultracentrifugation for Extracellular Vesicle Isolation from Tumor and Healthy Models
Source: Biomolecules. 2021 Dec 10;11(12):1857. doi: 10.3390/biom11121857 (PMC8699204; doi:10.3390/biom11121857)
Supplement: Supplementary file 1 [file biomolecules-11-01857-s001.zip › Figure S38.pdf]

NANOSIGHT

Batteri LE392 NaAc 2020-02-17 16-00-24

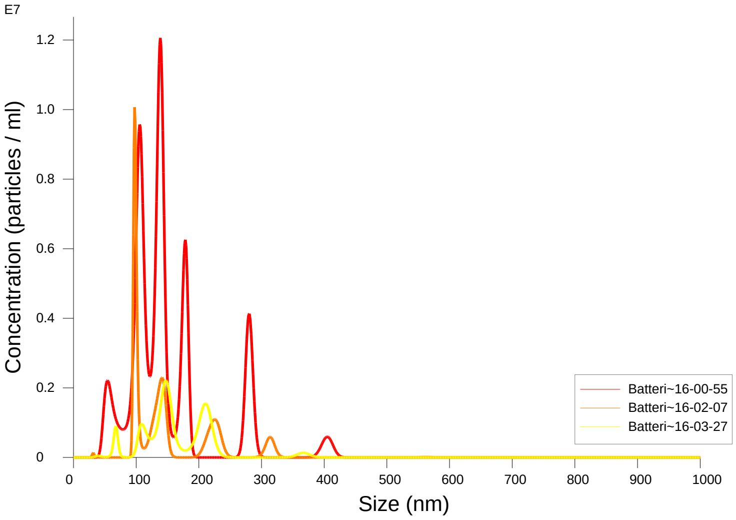

FTLA Concentration / Size graph for Experiment:
Batteri LE392 NaAc 2020-02-17 16-00-24

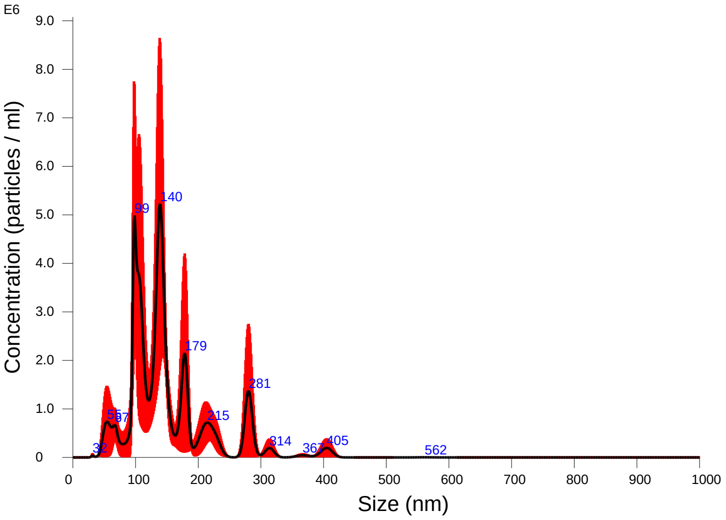

Averaged FTLA Concentration / Size for Experiment:
Batteri LE392 NaAc 2020-02-17 16-00-24
Error bars indicate + / -1 standard error of the mean

Included Files

Batteri LE392 NaAc 2020-02-17 16-00-55
Batteri LE392 NaAc 2020-02-17 16-02-07
Batteri LE392 NaAc 2020-02-17 16-03-27

Details

NTA Version:	NTA 3.2 Dev Build 3.2.16
Script Used:	SOP Standard Measurement 04-00-24PM 17~
Time Captured:	16:00:24 17/02/2020
Operator:	Antonio
Pre-treatment:	
Sample Name:	Batteri LE392 NaAc
Diluent:	
Remarks:	

Capture Settings

Camera Type:	sCMOS
Laser Type:	Green
Camera Level:	13
Slider Shutter:	1232
Slider Gain:	219
FPS	25.0
Number of Frames:	1498
Temperature:	22.0 ^{o}C
Viscosity:	(Water) 1.0 cP
Dilution factor:	Dilution not recorded

Analysis Settings

Detect Threshold:	10
Blur Size:	Auto
Max Jump Distance:	Auto: 12.0 - 15.9 pix

Results

Stats: Merged Data

Mean:	150.7 nm
Mode:	139.0 nm
SD:	67.0 nm
D10:	94.3 nm
D50:	135.8 nm
D90:	268.9 nm

Stats: Mean +/- Standard Error

Mean:	153.5 +/- 5.0 nm
Mode:	127.9 +/- 15.3 nm
SD:	63.4 +/- 4.4 nm
D10:	96.1 +/- 4.9 nm
D50:	138.1 +/- 6.7 nm
D90:	241.8 +/- 17.6 nm
Concentration:	2.92e+008 +/- 1.47e+008 particles/ml
	14.8 +/- 7.5 particles/frame
	25.1 +/- 15.7 centres/frame

Batteri LE392 NaAc 2020-02-17 16-00-24

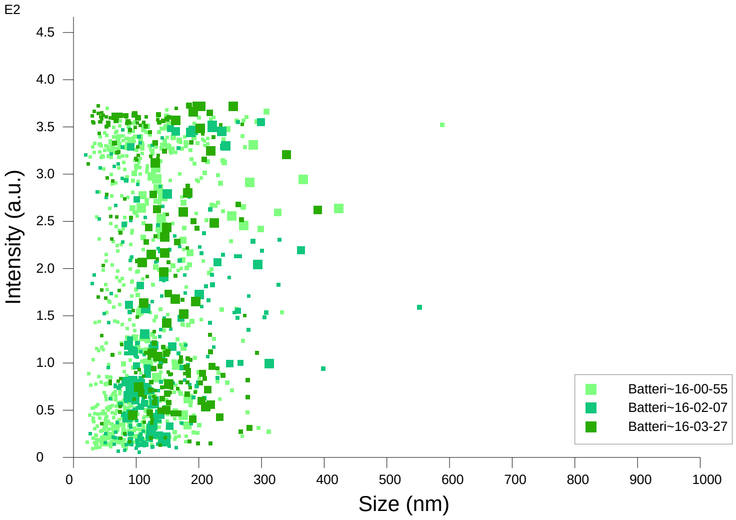

Intensity / Size graph for Experiment:
Batteri LE392 NaAc 2020-02-17 16-00-24

Script Used: (Full Text):

SOP Standard Measurement 04-00-24PM 17Feb2020.txt
