# Supplementary material for: Salting-Out Approach Is Worthy of Comparison with Ultracentrifugation for Extracellular Vesicle Isolation from Tumor and Healthy Models
Source: Biomolecules. 2021 Dec 10;11(12):1857. doi: 10.3390/biom11121857 (PMC8699204; doi:10.3390/biom11121857)
Supplement: Supplementary file 1 [file biomolecules-11-01857-s001.zip › Figure S39.pdf]

# NANOSIGHT

# Drosophila_NaAc 2019-11-15 17-04-26

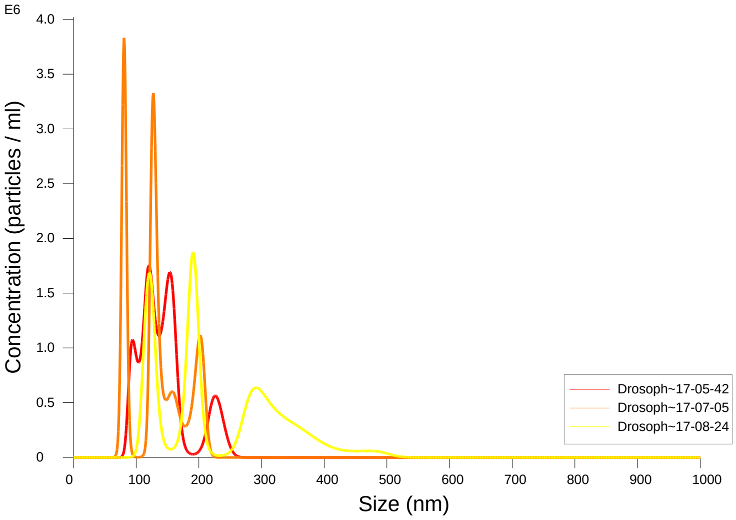


FTLA Concentration / Size graph for Experiment:
Drosophila_NaAc 2019-11-15 17-04-26

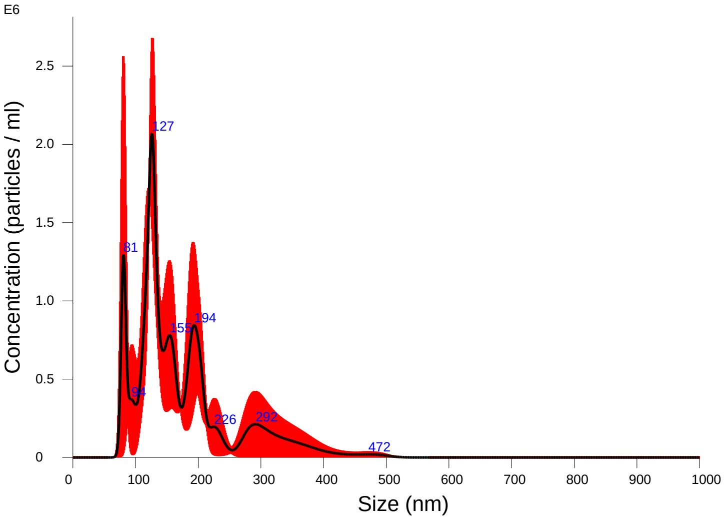


Averaged FTLA Concentration / Size for Experiment:
Drosophila_NaAc 2019-11-15 17-04-26
Error bars indicate + / -1 standard error of the mean

## Included Files

Drosophila_NaAc 2019-11-15 17-05-42
Drosophila_NaAc 2019-11-15 17-07-05
Drosophila_NaAc 2019-11-15 17-08-24

## Details

| | |
|---|---|
| NTA Version: | NTA 3.2 Dev Build 3.2.16 |
| Script Used: | SOP Standard Measurement 05-04-26PM 15~ |
| Time Captured: | 17:04:26 15/11/2019 |
| Operator: | Antonio |
| Pre-treatment: | |
| Sample Name: | Drosophila_NaAc |
| Diluent: | |
| Remarks: | |

## Capture Settings

| | |
|---|---|
| Camera Type: | sCMOS |
| Laser Type: | Green |
| Camera Level: | 14 |
| Slider Shutter: | 1259 |
| Slider Gain: | 366 |
| FPS | 25.0 |
| Number of Frames: | 1498 |
| Temperature: | 25.0 - 25.0 $^{o}$C |
| Viscosity: | (Water) 0.9 cP |
| Dilution factor: | Dilution not recorded |

## Analysis Settings

| | |
|---|---|
| Detect Threshold: | 5 |
| Blur Size: | Auto |
| Max Jump Distance: | Auto: 9.6 - 14.0 pix |

## Results

Stats: Merged Data

| | |
|---|---|
| Mean: | 172.3 nm |
| Mode: | 126.1 nm |
| SD: | 80.8 nm |
| D10: | 88.1 nm |
| D50: | 145.2 nm |
| D90: | 299.6 nm |

Stats: Mean +/- Standard Error

| | |
|---|---|
| Mean: | 169.1 +/- 31.1 nm |
| Mode: | 130.7 +/- 32.2 nm |
| SD: | 59.7 +/- 18.4 nm |
| D10: | 97.7 +/- 11.4 nm |
| D50: | 153.2 +/- 21.3 nm |
| D90: | 259.6 +/- 51.1 nm |
| Concentration: | 1.32e+008 +/- 7.05e+006 particles/ml |
| | 6.7 +/- 0.4 particles/frame |
| | 7.7 +/- 0.2 centres/frame |

NANOSIGHT

# Drosophila_NaAc 2019-11-15 17-04-26

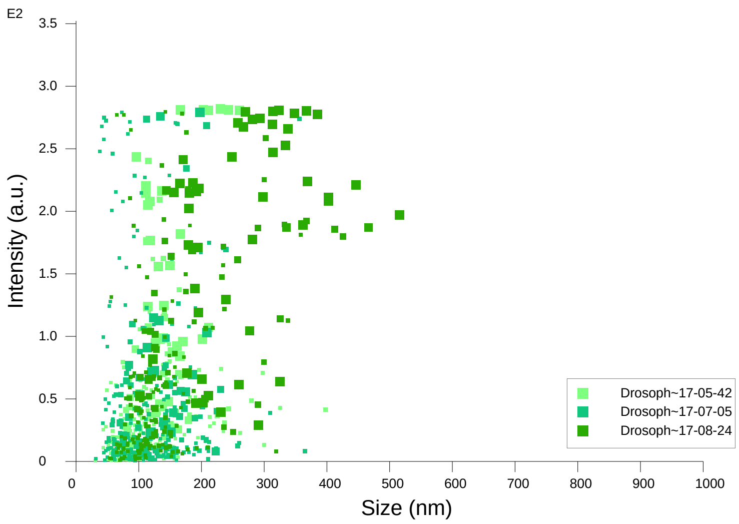


Intensity / Size graph for Experiment:
Drosophila_NaAc 2019-11-15 17-04-26

**Script Used: (Full Text):**

SOP Standard Measurement 05-04-26PM 15Nov2019.txt
