# Supplementary material for: Salting-Out Approach Is Worthy of Comparison with Ultracentrifugation for Extracellular Vesicle Isolation from Tumor and Healthy Models
Source: Biomolecules. 2021 Dec 10;11(12):1857. doi: 10.3390/biom11121857 (PMC8699204; doi:10.3390/biom11121857)
Supplement: Supplementary file 1 [file biomolecules-11-01857-s001.zip › Figure S40.pdf]

**NANO**SIGHT

# Drosophila_U 2019-11-15 16-55-00

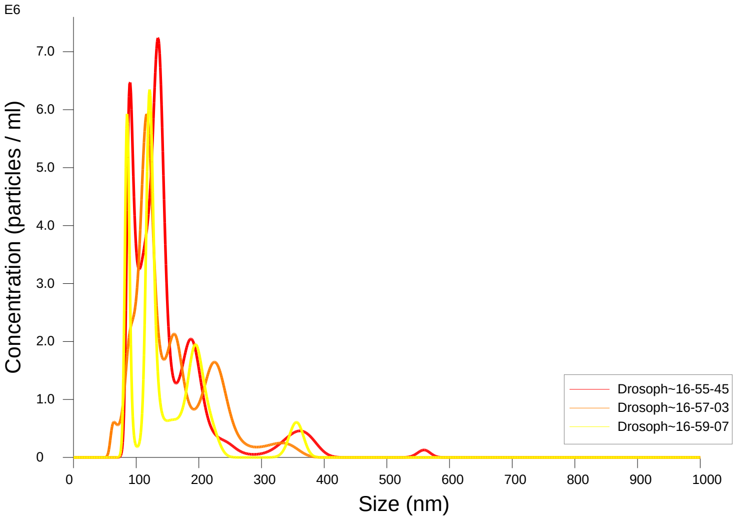


FTLA Concentration / Size graph for Experiment:
Drosophila_U 2019-11-15 16-55-00

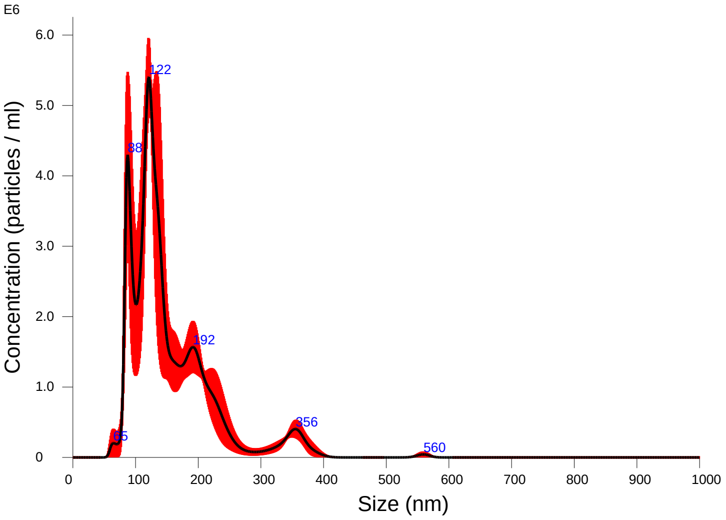


Averaged FTLA Concentration / Size for Experiment:
Drosophila_U 2019-11-15 16-55-00
Error bars indicate + / -1 standard error of the mean

## Included Files

Drosophila_U 2019-11-15 16-55-45
Drosophila_U 2019-11-15 16-57-03
Drosophila_U 2019-11-15 16-59-07

## Details

| | |
|---|---|
| NTA Version: | NTA 3.2 Dev Build 3.2.16 |
| Script Used: | SOP Standard Measurement 04-55-00PM 15~ |
| Time Captured: | 16:55:00 15/11/2019 |
| Operator: | Antonio |
| Pre-treatment: | |
| Sample Name: | Drosophila_U |
| Diluent: | |
| Remarks: | |

## Capture Settings

| | |
|---|---|
| Camera Type: | sCMOS |
| Laser Type: | Green |
| Camera Level: | 14 |
| Slider Shutter: | 1259 |
| Slider Gain: | 366 |
| FPS | 25.0 |
| Number of Frames: | 1498 |
| Temperature: | 25.0 - 25.0 $^{o}$C |
| Viscosity: | (Water) 0.9 cP |
| Dilution factor: | Dilution not recorded |

## Analysis Settings

| | |
|---|---|
| Detect Threshold: | 5 |
| Blur Size: | Auto |
| Max Jump Distance: | Auto: 12.1 - 12.7 pix |

## Results

Stats: Merged Data

| | |
|---|---|
| Mean: | 153.7 nm |
| Mode: | 121.2 nm |
| SD: | 67.9 nm |
| D10: | 88.9 nm |
| D50: | 131.1 nm |
| D90: | 229.6 nm |

Stats: Mean +/- Standard Error

| | |
|---|---|
| Mean: | 153.7 +/- 0.8 nm |
| Mode: | 124.4 +/- 5.4 nm |
| SD: | 67.4 +/- 3.3 nm |
| D10: | 89.5 +/- 2.5 nm |
| D50: | 130.3 +/- 1.9 nm |
| D90: | 224.0 +/- 7.3 nm |
| Concentration: | 3.83e+008 +/- 5.95e+007 particles/ml |
| | 19.4 +/- 3.0 particles/frame |
| | 20.8 +/- 3.1 centres/frame |

# Drosophila_U 2019-11-15 16-55-00

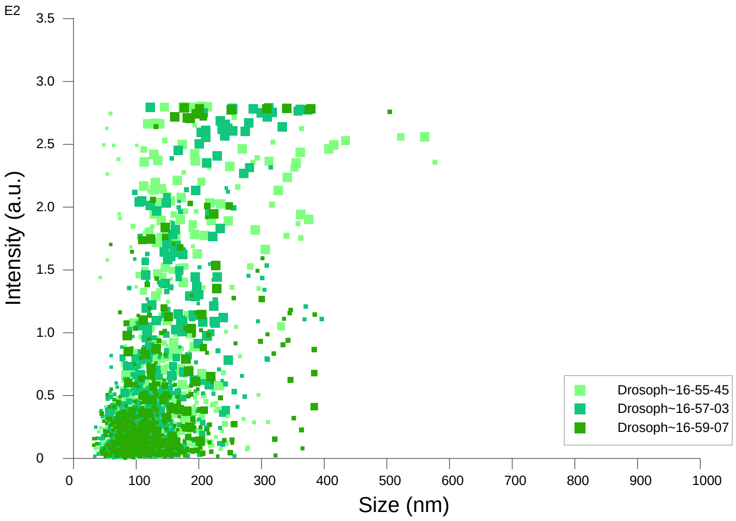


Intensity / Size graph for Experiment:
Drosophila_U 2019-11-15 16-55-00

**Script Used: (Full Text):**

SOP Standard Measurement 04-55-00PM 15Nov2019.txt
