# Supplementary material for: Salting-Out Approach Is Worthy of Comparison with Ultracentrifugation for Extracellular Vesicle Isolation from Tumor and Healthy Models
Source: Biomolecules. 2021 Dec 10;11(12):1857. doi: 10.3390/biom11121857 (PMC8699204; doi:10.3390/biom11121857)
Supplement: Supplementary file 1 [file biomolecules-11-01857-s001.zip › Figure S41.pdf]

NANOSIGHT

Lievito By4742 NaAc 2020-02-14 14-45-33

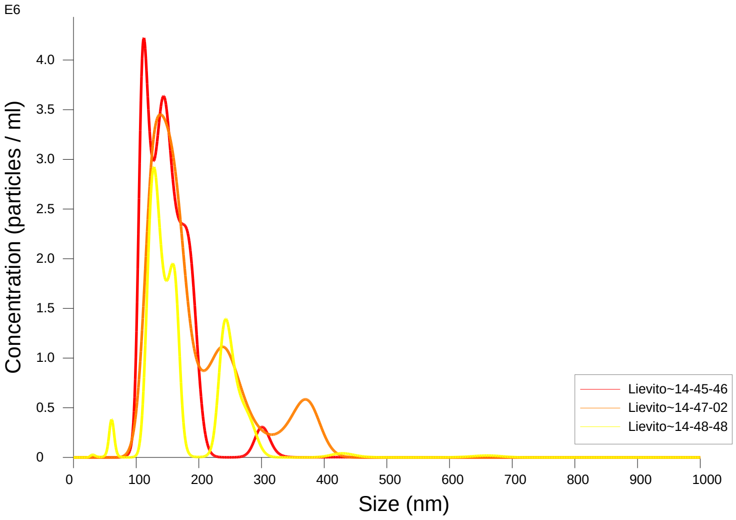

FTLA Concentration / Size graph for Experiment:
Lievito By4742 NaAc 2020-02-14 14-45-33

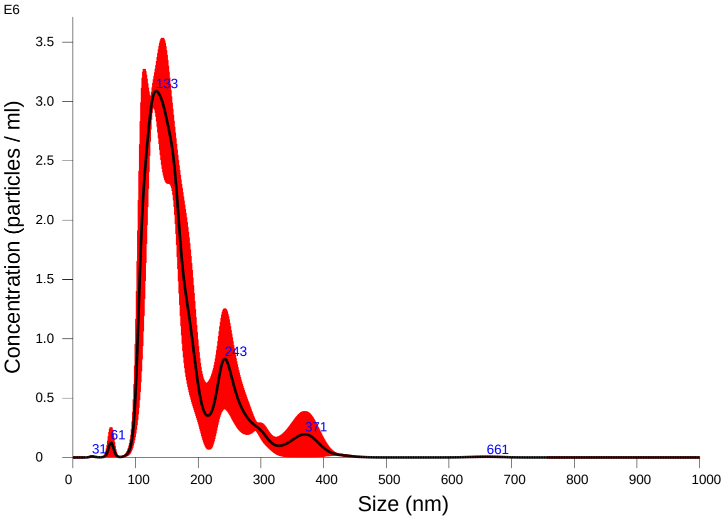

Averaged FTLA Concentration / Size for Experiment:
Lievito By4742 NaAc 2020-02-14 14-45-33
Error bars indicate + / -1 standard error of the mean

Included Files

Lievito By4742 NaAc 2020-02-14 14-45-46
Lievito By4742 NaAc 2020-02-14 14-47-02
Lievito By4742 NaAc 2020-02-14 14-48-48

Details

NTA Version:	NTA 3.2 Dev Build 3.2.16
Script Used:	SOP Standard Measurement 02-45-33PM 14~
Time Captured:	14:45:33 14/02/2020
Operator:	Antonio
Pre-treatment:	
Sample Name:	Lievito By4742 NaAc
Diluent:	
Remarks:	

Capture Settings

Camera Type:	sCMOS
Laser Type:	Green
Camera Level:	12
Slider Shutter:	1200
Slider Gain:	146
FPS	25.0
Number of Frames:	1498
Temperature:	23.8 - 23.9 ^{o}C
Viscosity:	(Water) 0.912 - 0.915 cP
Dilution factor:	Dilution not recorded

Analysis Settings

Detect Threshold:	5
Blur Size:	Auto
Max Jump Distance:	Auto: 9.6 - 13.0 pix

Results

Stats: Merged Data

Mean:	174.5 nm
Mode:	132.8 nm
SD:	67.2 nm
D10:	114.3 nm
D50:	152.8 nm
D90:	263.8 nm

Stats: Mean +/- Standard Error

Mean:	173.8 +/- 12.6 nm
Mode:	126.4 +/- 7.7 nm
SD:	62.8 +/- 11.6 nm
D10:	116.1 +/- 3.8 nm
D50:	153.0 +/- 6.2 nm
D90:	257.3 +/- 38.2 nm
Concentration:	2.81e+008 +/- 4.95e+007 particles/ml
	14.3 +/- 2.5 particles/frame
	18.8 +/- 2.8 centres/frame

NANOSIGHT

Lievito By4742 NaAc 2020-02-14 14-45-33

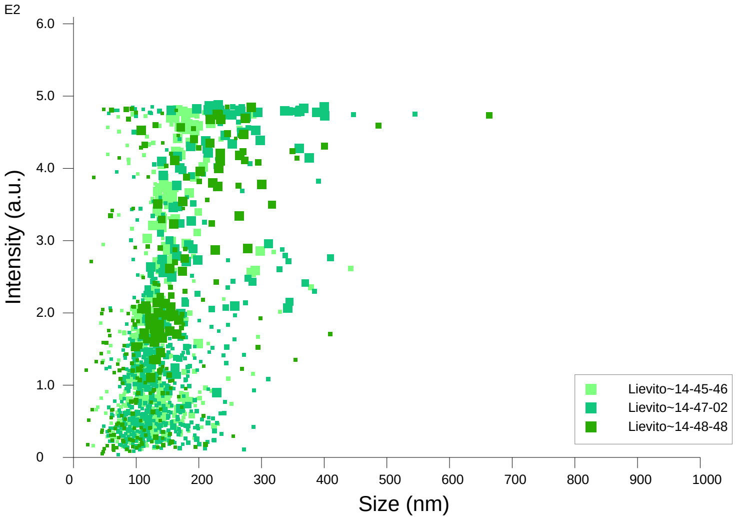

Intensity / Size graph for Experiment:
Lievito By4742 NaAc 2020-02-14 14-45-33

Script Used: (Full Text):

SOP Standard Measurement 02-45-33PM 14Feb2020.txt
