# Supplementary material for: Salting-Out Approach Is Worthy of Comparison with Ultracentrifugation for Extracellular Vesicle Isolation from Tumor and Healthy Models
Source: Biomolecules. 2021 Dec 10;11(12):1857. doi: 10.3390/biom11121857 (PMC8699204; doi:10.3390/biom11121857)
Supplement: Supplementary file 1 [file biomolecules-11-01857-s001.zip › Figure S42.pdf]

NANOSIGHT

Lievito By4742 Ultra 2020-02-14 14-31-50

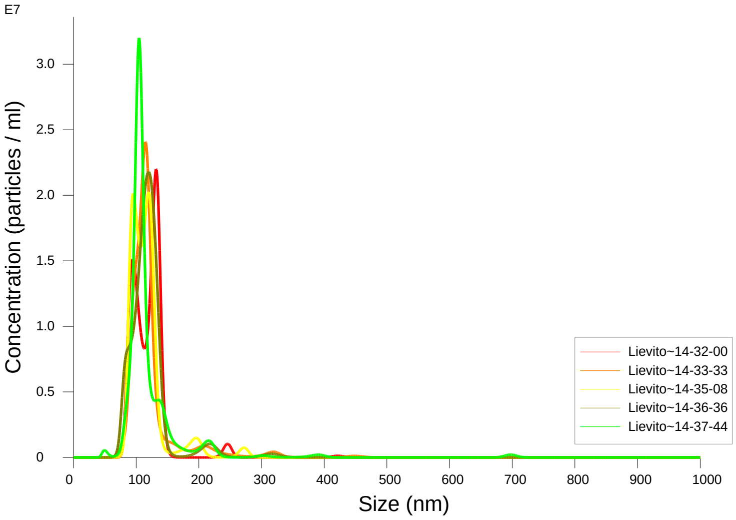

FTLA Concentration / Size graph for Experiment:
Lievito By4742 Ultra 2020-02-14 14-31-50

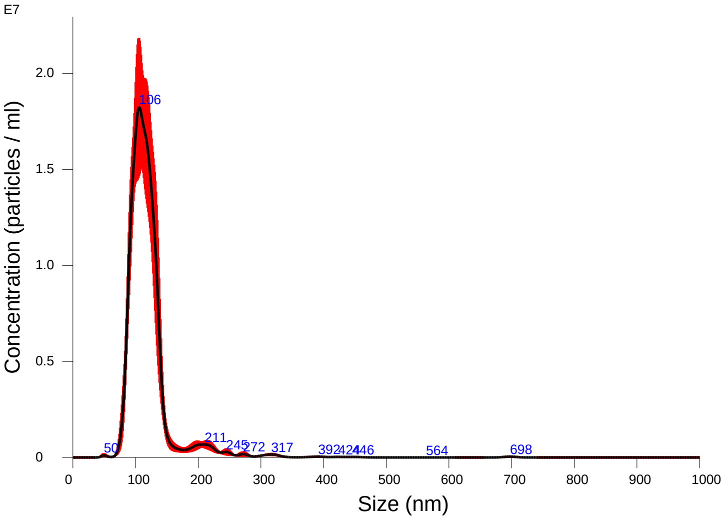

Averaged FTLA Concentration / Size for Experiment:
Lievito By4742 Ultra 2020-02-14 14-31-50
Error bars indicate + / -1 standard error of the mean

Included Files

Lievito By4742 Ultra 2020-02-14 14-32-00
Lievito By4742 Ultra 2020-02-14 14-33-33
Lievito By4742 Ultra 2020-02-14 14-35-08
Lievito By4742 Ultra 2020-02-14 14-36-36
Lievito By4742 Ultra 2020-02-14 14-37-44

Details

NTA Version:	NTA 3.2 Dev Build 3.2.16
Script Used:	SOP Standard Measurement 02-31-50PM 14~
Time Captured:	14:31:50 14/02/2020
Operator:	Antonio
Pre-treatment:	
Sample Name:	Lievito By4742 Ultra
Diluent:	
Remarks:	

Capture Settings

Camera Type:	sCMOS
Laser Type:	Green
Camera Level:	14
Slider Shutter:	1259
Slider Gain:	366
FPS	25.0
Number of Frames:	1498
Temperature:	23.3 - 23.6 ^{o}C
Viscosity:	(Water) 0.918 - 0.924 cP
Dilution factor:	Dilution not recorded

Analysis Settings

Detect Threshold:	5
Blur Size:	Auto
Max Jump Distance:	Auto: 12.8 - 13.2 pix

Results

Stats: Merged Data

Mean:	119.8 nm
Mode:	105.9 nm
SD:	41.0 nm
D10:	90.3 nm
D50:	111.7 nm
D90:	139.6 nm

Stats: Mean +/- Standard Error

Mean:	119.9 +/- 1.1 nm
Mode:	118.2 +/- 4.4 nm
SD:	39.7 +/- 5.4 nm
D10:	90.3 +/- 1.0 nm
D50:	112.6 +/- 2.1 nm
D90:	142.6 +/- 4.1 nm
Concentration:	8.78e+008 +/- 2.55e+007 particles/ml
	44.6 +/- 1.3 particles/frame
	51.5 +/- 2.0 centres/frame

NANOSIGHT

Lievito By4742 Ultra 2020-02-14 14-31-50

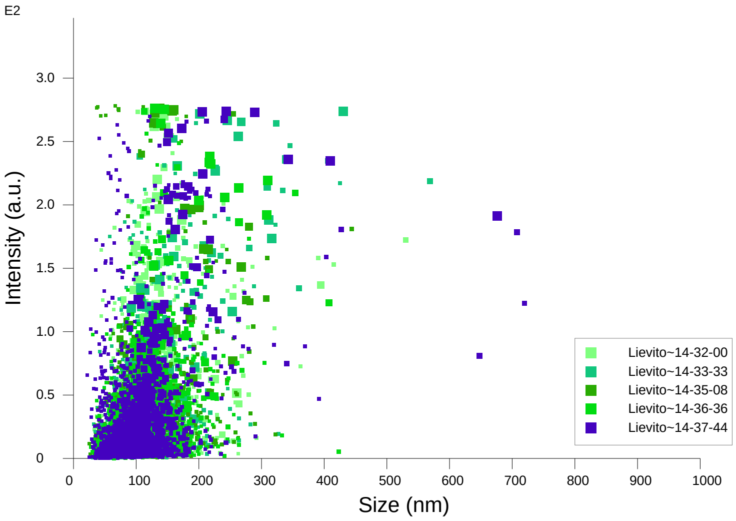

Intensity / Size graph for Experiment:
Lievito By4742 Ultra 2020-02-14 14-31-50

Script Used: (Full Text):

SOP Standard Measurement 02-31-50PM 14Feb2020.txt
