# Supplementary material for: Salting-Out Approach Is Worthy of Comparison with Ultracentrifugation for Extracellular Vesicle Isolation from Tumor and Healthy Models
Source: Biomolecules. 2021 Dec 10;11(12):1857. doi: 10.3390/biom11121857 (PMC8699204; doi:10.3390/biom11121857)
Supplement: Supplementary file 1 [file biomolecules-11-01857-s001.zip › Figure S9.pdf]

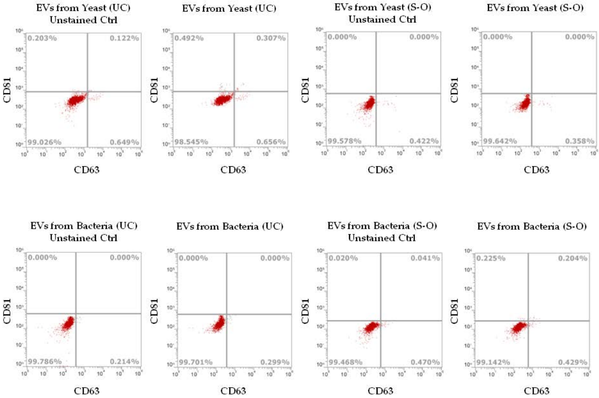
EVs from Yeast (UC)
Unstained Ctrl
0.203%
0.122%
99.026%
0.649%
CD81
CD63
EVs from Yeast (UC)
0.492%
0.307%
98.545%
0.656%
CD81
CD63
EVs from Yeast (S-O)
Unstained Ctrl
0.000%
0.000%
99.578%
0.422%
CD81
CD63
EVs from Yeast (S-O)
0.000%
0.000%
99.642%
0.358%
CD81
CD63
EVs from Bacteria (UC)
Unstained Ctrl
0.000%
0.000%
99.786%
0.214%
CD81
CD63
EVs from Bacteria (UC)
0.000%
0.000%
99.701%
0.299%
CD81
CD63
EVs from Bacteria (S-O)
Unstained Ctrl
0.020%
0.041%
99.468%
0.470%
CD81
CD63
EVs from Bacteria (S-O)
0.225%
0.204%
99.142%
0.429%
CD81
CD63
