# Supplementary material for: Salting-Out Approach Is Worthy of Comparison with Ultracentrifugation for Extracellular Vesicle Isolation from Tumor and Healthy Models
Source: Biomolecules. 2021 Dec 10;11(12):1857. doi: 10.3390/biom11121857 (PMC8699204; doi:10.3390/biom11121857)
Supplement: Supplementary file 1 [file biomolecules-11-01857-s001.zip › Supplementary File S1.pdf]

M&M S1. EVs analysis by Flow Cytometry.

To prepare and setup the instrument Attune NxT Flow Cytometer (Thermo Fisher) configured with 4 spatially separated lasers, red, green, yellow and violet and with the specific filter for small particles (Attune™ NxT OD2-488/10 Filter) we utilised Gorgun's protocol [51].

All solutions utilised in the experiments were filtered with 0.22-µm filter.

For the setup of EV's size, we utilised two different mix of fluorescent beads of varied diameters (0.1 to 1 µm): A)Megamix-Plus SSC (Biocytex) and B) a Mix (1:1) of Megamix-Plus SSC (Biocytex) and Megamix-Plus FCS (Biocytex) .

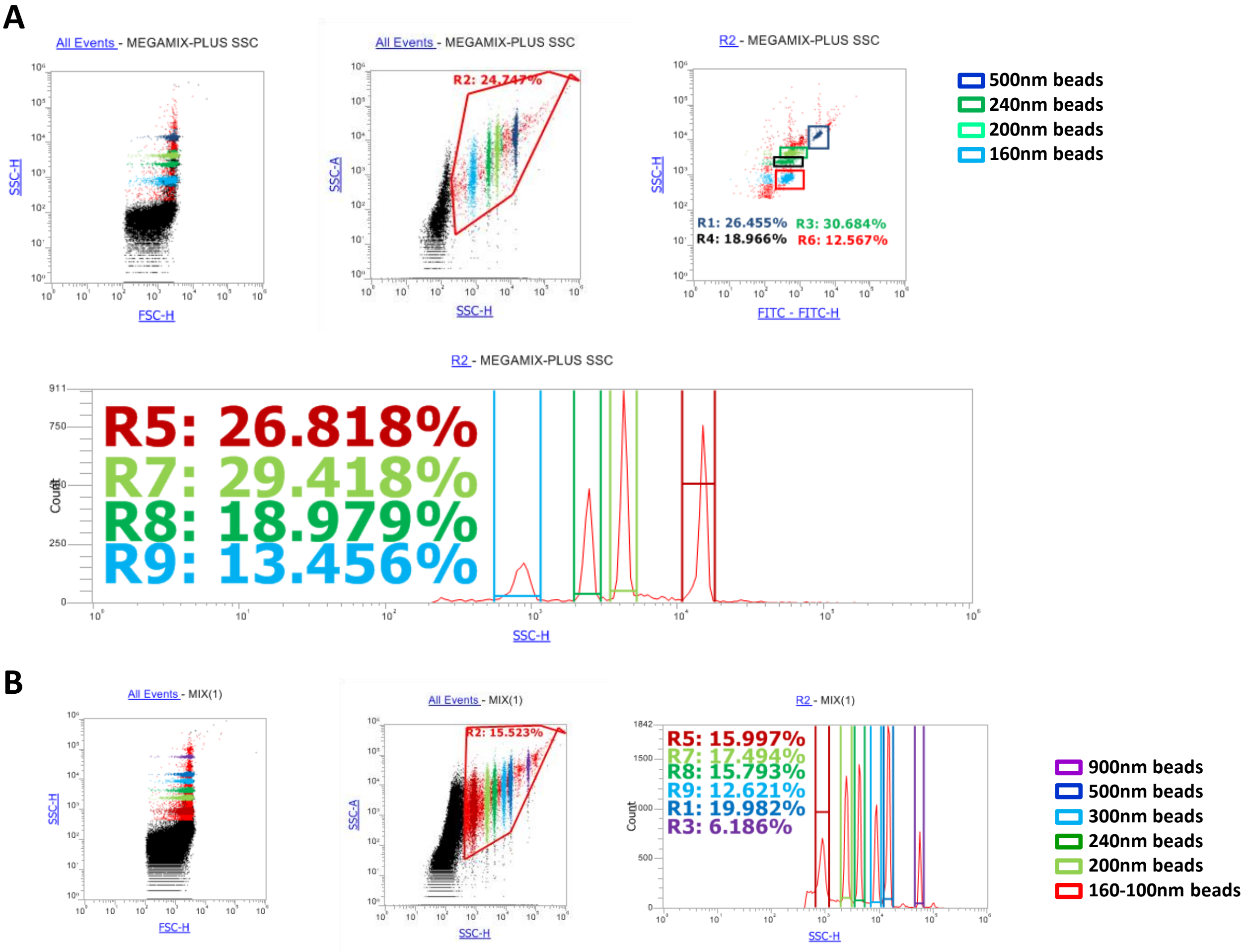

The physical parameters utilised were: FSC: 80 and SSC: 220. The setup of the all fluorescences were made during the compensation procedure with the Fluorescence Minus One (FMO) control, following the instrument instruction. CD63-CD63-PE-Cy7 was excited with laser yellow (561nm) and retrieved with the channel YL4 (PMT: 400) and CD81-APC was excited with laser red (637nm) and retrieved with the channel YL3 (PMT: 400). The threshold was on FSC (1.0 x 1000).

The analysis of each EV sample was carried out by incubating EVs with or without the specific fluorescent primary antibodies (reported in M&M section). For each sample, we collected 10.000 events of the population gated in the dot plot SSC-A vs SSC-H. In the following figure, the analysis for the unstained EVs from plasma, isolated by Salting-out, is reported, in which the positivity of CD63, CD81 and ALIX were investigated by comparing results obtained with the unstained samples with the same stained with Abs.

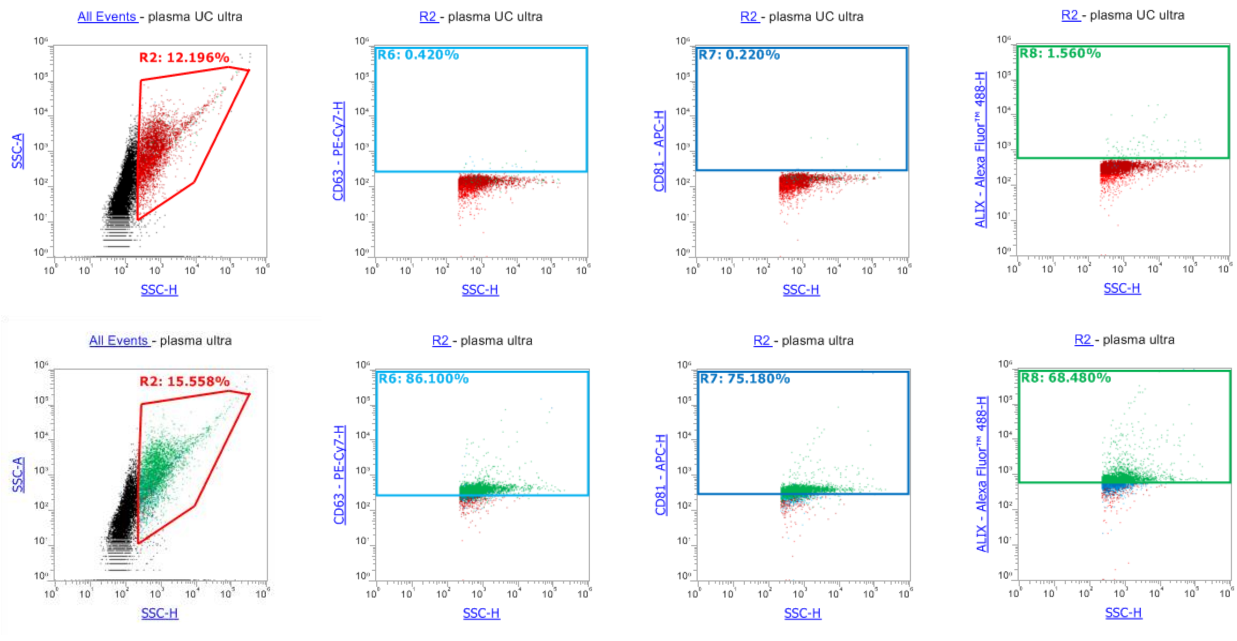
All Events - plasma UC ultra
R2: 12.196%
SSC-A
SSC-H
R2 - plasma UC ultra
R6: 0.420%
CD63 - PE-Cy7-H
SSC-H
R2 - plasma UC ultra
R7: 0.220%
CD81 - APC-H
SSC-H
R2 - plasma UC ultra
R8: 1.560%
ALIX - Alexa Fluor™ 488-H
SSC-H
All Events - plasma ultra
R2: 15.558%
SSC-A
SSC-H
R2 - plasma ultra
R6: 86.100%
CD63 - PE-Cy7-H
SSC-H
R2 - plasma ultra
R7: 75.180%
CD81 - APC-H
SSC-H
R2 - plasma ultra
R8: 68.480%
ALIX - Alexa Fluor™ 488-H
SSC-H
