# Supplementary material for: Salting-Out Approach Is Worthy of Comparison with Ultracentrifugation for Extracellular Vesicle Isolation from Tumor and Healthy Models
Source: Biomolecules. 2021 Dec 10;11(12):1857. doi: 10.3390/biom11121857 (PMC8699204; doi:10.3390/biom11121857)
Supplement: Supplementary file 1 [file biomolecules-11-01857-s001.zip › Supplementary Table S1.pdf]

Table S1. EVs isolation characteristics

Samples	Supports for Cell Cultures	Cells (10^6)	EV Isolation Volume	EVs Isolated by UC (100 µl)	UC EVs Yield (µg)	EVs Isolated by S-O (in 100 µl)	S-O EVs Yields (µg)
Saliva_UC (1)				$(6.7 \pm 0.3) \times 10^{10}$	$(8.3 \pm 2.1) \times 10^{2}$	$(2.9 \pm 0.4) \times 10^{10}$	$(3.6 \pm 1.9) \times 10^{2}$
Saliva_UC (2)			2ml	$(9.80 \pm 0.9) \times 10^{9}$	$(1.17 \pm 0.3) \times 10^{2}$	$(4.10 \pm 0.2) \times 10^{9}$	$(0.56 \pm 0.1) \times 10^{2}$
Saliva_UC (3)				$(3.74 \pm 0.2) \times 10^{10}$	$(4.35 \pm 0.2) \times 10^{2}$	$(2.13 \pm 0.2) \times 10^{10}$	$(2.35 \pm 0.5) \times 10^{2}$
Plasma_UC (1)				$(1.7 \pm 0.1) \times 10^{11}$	$(16.5 \pm 4.6) \times 10^{2}$	$(2.3 \pm 0.3) \times 10^{10}$	$(2.8 \pm 1.7) \times 10^{2}$
Plasma_UC (2)			4ml	$(2.71 \pm 0.2) \times 10^{10}$	$(3.3 \pm 0.01) \times 10^{2}$	$(5.24 \pm 1.1) \times 10^{10}$	$(6.7 \pm 0.01) \times 10^{2}$
Plasma_UC (3)				$(1.93 \pm 0.1) \times 10^{10}$	$(2.9 \pm 0.01) \times 10^{2}$	$(2.82 \pm 0.3) \times 10^{10}$	$(3.5 \pm 0.01) \times 10^{2}$
Urine_UC (1)				$(2.2 \pm 0.1) \times 10^{11}$	$(28.7 \pm 35.8) \times 10^{2}$	$(3.5 \pm 0.1) \times 10^{10}$	$(4.1 \pm 1.6) \times 10^{2}$
Urine_UC (2)			50ml	$(6.65 \pm 0.1) \times 10^{10}$	$(7.4 \pm 0.1) \times 10^{2}$	$(6.49 \pm 0.6) \times 10^{9}$	$(0.95 \pm 0.02) \times 10^{2}$
Urine_UC (3)				$(2.59 \pm 0.2) \times 10^{10}$	$(3 \pm 0.08) \times 10^{2}$	$(2.13 \pm 0.2) \times 10^{10}$	$(2.5 \pm 0.05) \times 10^{2}$
Human cancer cells	flasks 75cm^2	24± 0.7	30ml	$(4.2 \pm 0.6) \times 10^{10}$	$(3.3 \pm 0.8) \times 10^{2}$	$(2.5 \pm 0.3) \times 10^{10}$	$(2.5 \pm 0.6) \times 10^{2}$
Human non cancer cells	flasks 75 cm^2	22± 0.3	30ml	$(6.8 \pm 0.4) \times 10^{10}$	$(3.1 \pm 0.4) \times 10^{2}$	$(3.3 \pm 0.1) \times 10^{10}$	$(2.6 \pm 0.5) \times 10^{2}$
Insect cells	flasks 75 cm^2	61± 1	30ml	$(6.3 \pm 0.3) \times 10^{10}$	$(2.8 \pm 0.4) \times 10^{2}$	$(2.1 \pm 0.2) \times 10^{10}$	$(1.3 \pm 0.2) \times 10^{2}$
Unicellular organisms	flasks 75 cm^2	0,6±0,1 (OD)	50ml	$(3.3 \pm 0.1) \times 10^{10}$	$(6.2 \pm 0.7) \times 10^{2}$	$(2.9 \pm 1.5) \times 10^{10}$	$(2.9 \pm 0.5) \times 10^{2}$
