# Supplementary figures and images for: Salting-Out Approach Is Worthy of Comparison with Ultracentrifugation for Extracellular Vesicle Isolation from Tumor and Healthy Models
Source: Biomolecules. 2021 Dec 10;11(12):1857. doi: 10.3390/biom11121857 (PMC8699204; doi:10.3390/biom11121857)

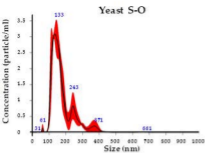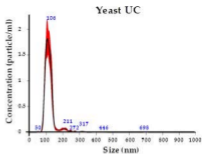

**a**

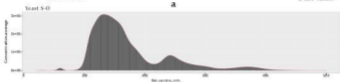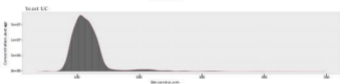

**b**

**HB2 UC**

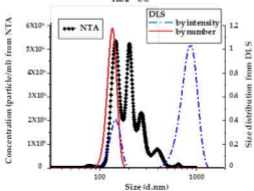

**c**

Supplement: Supplementary file 1 [file biomolecules-11-01857-s001.zip › Figure S1.pdf]

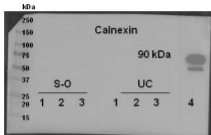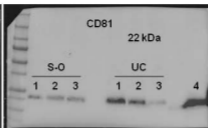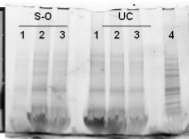

STAIN FREE GEL

1. Plasma EV
2. Colo205 EV
3. 8701BC EV
4. Colo205 cell lysate

Supplement: Supplementary file 1 [file biomolecules-11-01857-s001.zip › Figure S10.pdf]

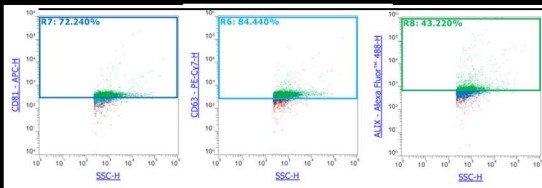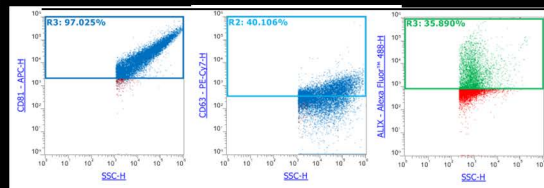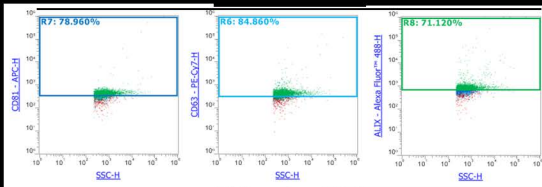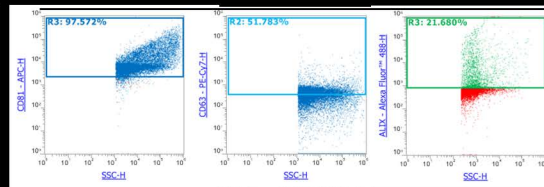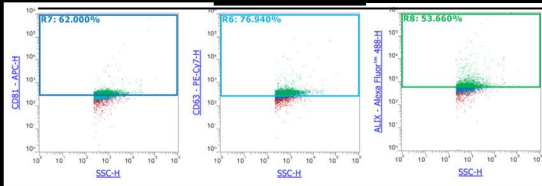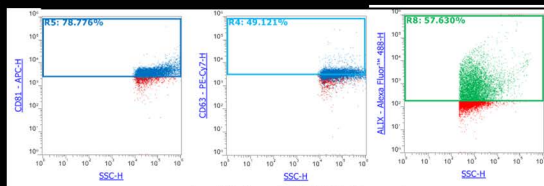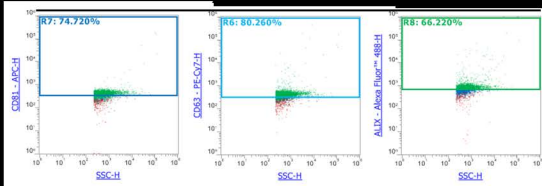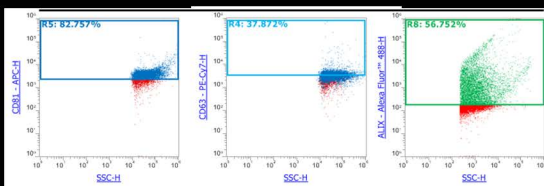

Supplement: Supplementary file 1 [file biomolecules-11-01857-s001.zip › Figure S3.pdf]

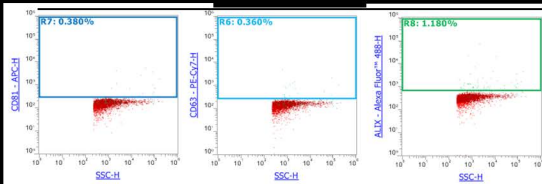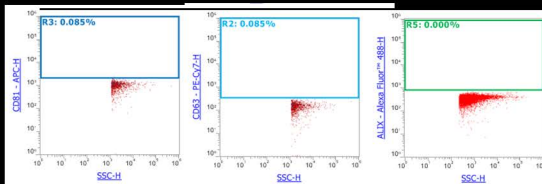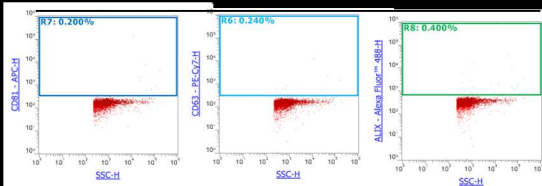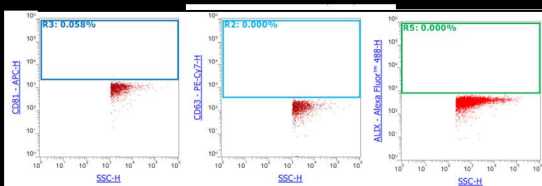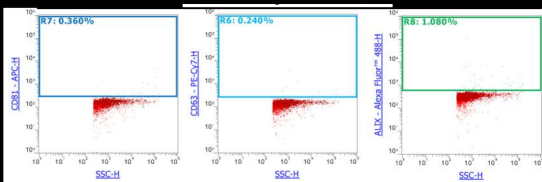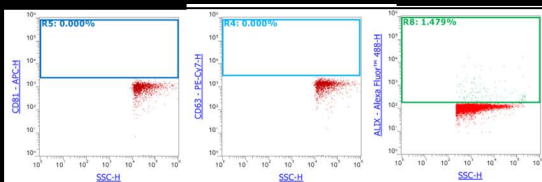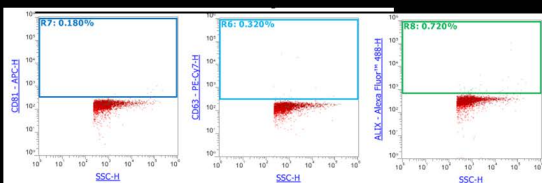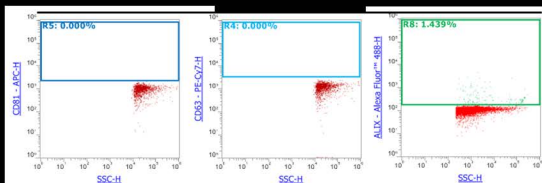

Supplement: Supplementary file 1 [file biomolecules-11-01857-s001.zip › Figure S4.pdf]

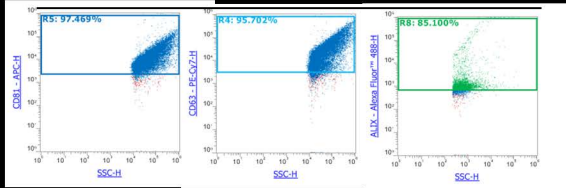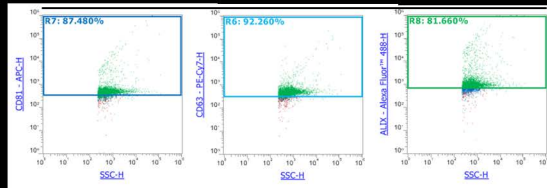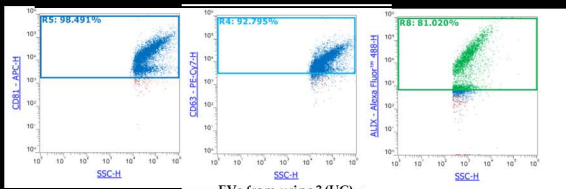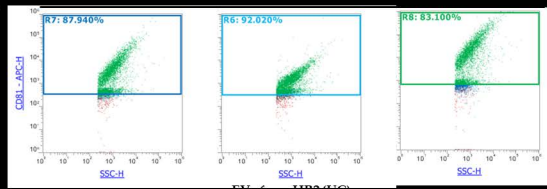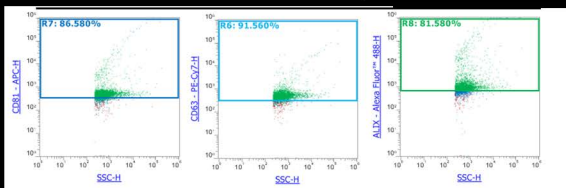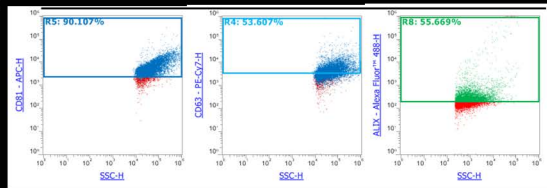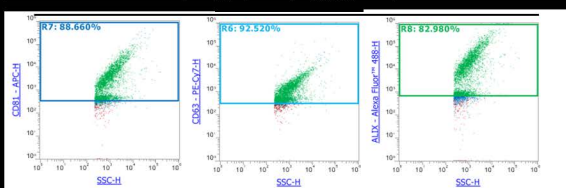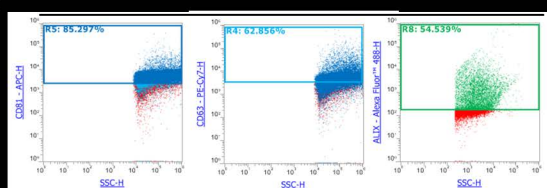

Supplement: Supplementary file 1 [file biomolecules-11-01857-s001.zip › Figure S5.pdf]

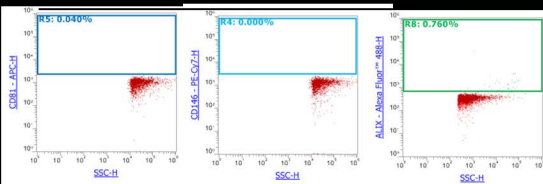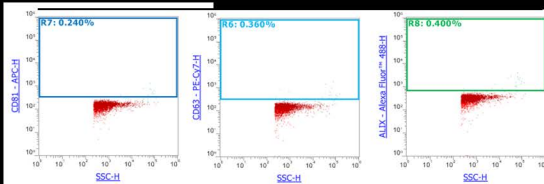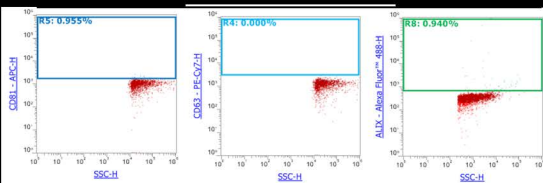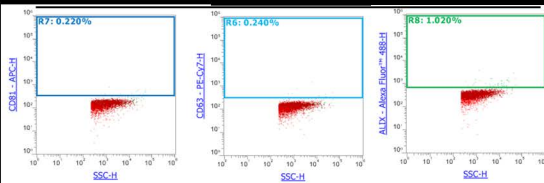

EVs from urine3 (UC)

EVs from MP3 (UC)

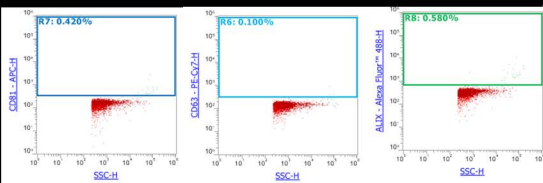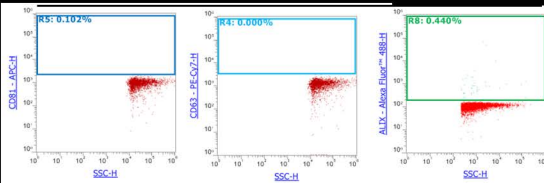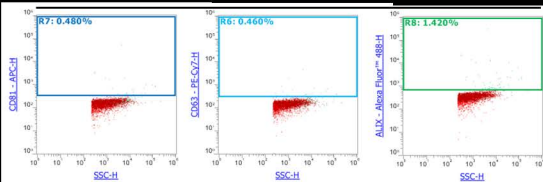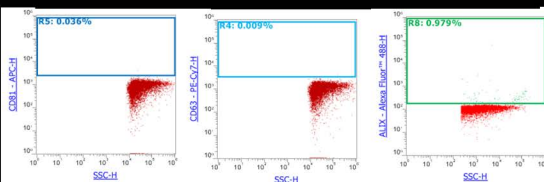

Supplement: Supplementary file 1 [file biomolecules-11-01857-s001.zip › Figure S6.pdf]

EVs from SRA1 (C5)

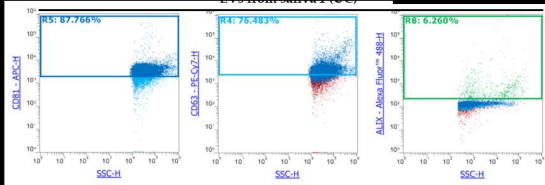

EVs from SRA1 (C5)

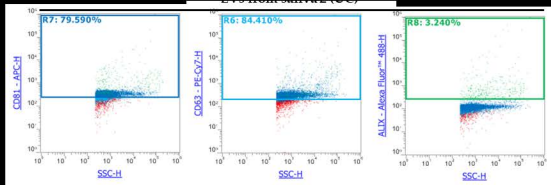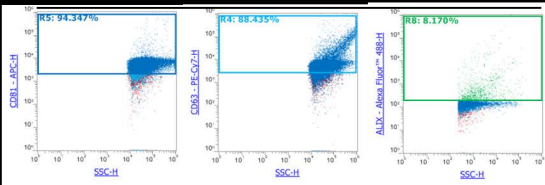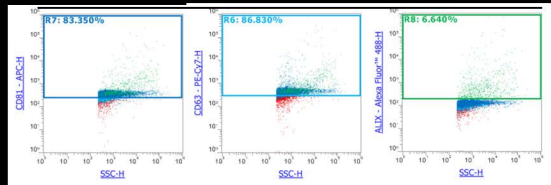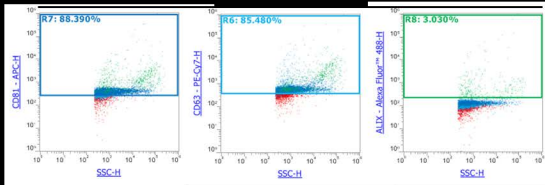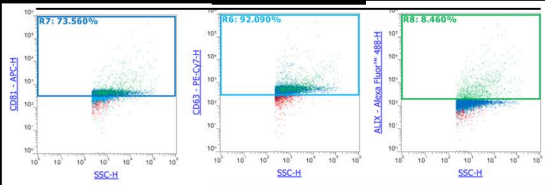

Supplement: Supplementary file 1 [file biomolecules-11-01857-s001.zip › Figure S7.pdf]

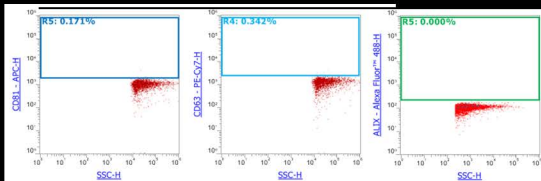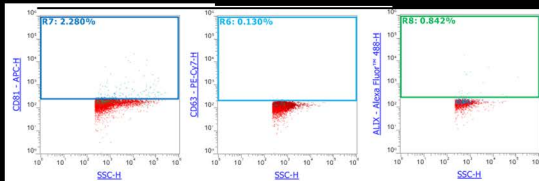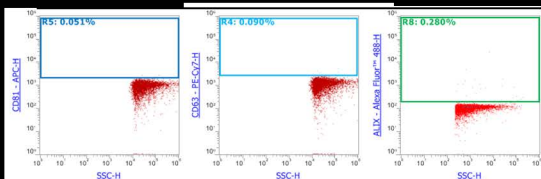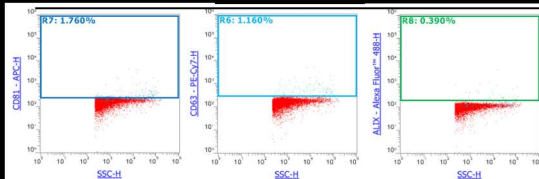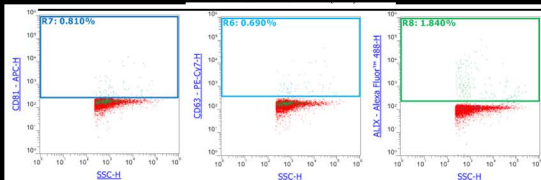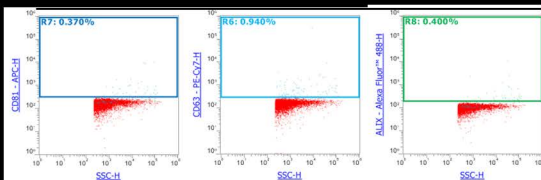

Supplement: Supplementary file 1 [file biomolecules-11-01857-s001.zip › Figure S8.pdf]
